# Patient-Reported Outcome Measures in the Emergency Department: A Scoping Review

**DOI:** 10.1016/j.acepjo.2026.100486

**Published:** 2026-08-17

**Authors:** Ninna R.H. Daugaard, Liv M.V. Schougaard, Morten B. Søvsø, Isabelle M. Larsen, Kristine B. Øby, Mettine T. Madsen, Peter D.C. Leutscher, Mona K. Pedersen

**Affiliations:** 1 Centre for Clinical Research, North Denmark Regional Hospital, Hjoerring, Denmark; 2 Department of Emergency Medicine, North Denmark Regional Hospital, Hjoerring, Denmark; 3 Department of Clinical Medicine, Aalborg University, Aalborg, Denmark; 4 AmbuFlex, Centre for Patient-reported Outcomes, Gødstrup Hospital, Herning, Denmark; 5 Department of Clinical Medicine, Aarhus University, Aarhus, Denmark; 6 Centre for Prehospital and Emergency Research, Danish Centre for Health Services Research, Aalborg University Hospital, Aalborg, Denmark; 7 Mech-Sense, Department of Gastroenterology and Hepatology, Aalborg University Hospital, Aalborg, Denmark

**Keywords:** patient-reported outcome measures, scoping review, emergency department, adult patients

## Abstract

**Objectives:**

Patient-reported outcome measures (PROMs) are increasingly utilized to support clinical care by capturing health status from the patient’s perspective. Although the application of PROMs in an emergency department (ED) context is expanding, their scope remains poorly mapped. This scoping review aims to identify and characterize the PROMs used to assess the health status of adult patients in the ED in the initial clinical evaluation, with focus on supporting clinical decision-making and developing individual care and treatment plans.

**Methods:**

A systematic literature search was conducted using the MEDLINE, Embase, CINAHL, and PsychINFO databases to identify studies utilizing PROMs in ED settings published between January 2000 and February 2025. The review was performed in accordance with the Preferred Reporting Items for Systematic Reviews and Meta-Analyses (PRISMA) Extension for Scoping Reviews and the Joanna Briggs Institute guidelines.

**Results:**

The review identified 173 unique PROMs, within the literature utilizing measurement properties. The most prevalent themes were mental health, substance use, and older people; whereas, PROMs for general conditions remained relatively scarce. Twenty-three studies detailed the development of new PROMs, whereas 22 studies described the adaptation of existing PROMs for the ED setting. Notably, most of the identified studies and PROMs were employed primarily for research purposes, with limited emphasis on providing direct clinical feedback.

**Conclusion:**

The predominance of research-oriented PROMs underscores a potential gap in translating these measures into clinical practice. Furthermore, the findings suggest that most existing PROMs focus on identifying specific patient vulnerabilities, pointing toward a relative scarcity of tools designed to assess general medical conditions in the ED.

## 1 Introduction

### 1.1 Background

The emergency department (ED) is a complex health care setting, characterized by high patient volumes, a fast-paced environment, limited staffing resources, and a heterogeneous patient population.^1^^,^^2^ This multifaceted environment poses significant challenges for both patients and health care professionals (HCPs), potentially leading to insufficient information sharing and a disconnect between the patient’s lived experience and the HCPs clinical assessment. Patient-reported outcome (PRO) data may bridge this gap, supporting the development of personalized care plans.^3^ PRO data encompass patient’s health status, including physical and mental health, symptoms, functional level, and health-related quality of life,^4^ and are reported directly by the patient without interpretation by a HCP.^5^ To ensure the valid and reliable measurement of data, patient-reported outcome measures (PROMs) are employed, typically via self-administered or interview-administered questionnaires.^6^ Notably, although PROM is the standardized term, these tools are frequently referred to in the literature as questionnaires, scales, tools or instruments.^7^^,^^8^

Currently, PROMs are predominantly utilized in research settings at an aggregated level to evaluate health care quality and performance.^9^ Nevertheless, there is increasing interest in integrating PROMs with direct patient feedback into clinical workflows to enhance clinical decision-making.^9^^,^^10^ Although the implementation of PROMs has been shown to improve clinician-patient communication and support patient involvement and shared decision-making,^11^^,^^12^ existing evidence is largely limited to nonemergency settings. However, PROM can reveal physical or psychological issues that might otherwise be overlooked by HCPs during rapid clinical assessment.^13^ In this regard, PROMs can complement existing biological measures and physical examinations by providing standardized assessments of how patients experience their health, quality of life, and mental well-being.^10^

### 1.2 Importance

PROMs are increasingly being integrated into clinical practice, particularly for the management of patients with chronic and malignant diseases in outpatient settings.^14^^,^^15^ Consequently, the ED has emerged as a novel area of interest. However, the clinical application of PROMs in the ED, encompassing the entire patient population rather than specific subgroups, remains limited. Barriers to implementation include the inherent heterogeneity of the ED population, diverse assessment and treatment protocols, and the administrative and practical constraints of a high-acuity environment. Collectively, these factors present challenges for the development and implementation of general, broadly applicable PROMs.^16^ Some measurement tools have been developed for the ED population, eg, based on medical history taking.^17^^,^^18^ Additionally, generic PROMs have been tested in an ED population and in specific patient groups, such as older people.^19^^,^^20^

A comprehensive overview of PROMs utilized prospectively within the ED context is currently lacking. A preliminary search conducted in July 2020, and updated in February 2025, identified 2 scoping reviews investigating PROMs in acute care settings. However, one review focused on evaluating clinical care delivery, rather than clinical PROM application,^21^ whereas the other did not report on PROM data utilized for individual symptom or disease management.^22^

Prospectively, a comprehensive mapping of PROMs within EDs could facilitate the identification of tools suitable for clinical integration. Consequently, this scoping review serves as a significant resource for clinicians or researchers aiming to implement PROMs into clinical practice or research settings. Given the absence of a standardized definition for PROMs, the anticipated heterogeneity of the available literature, and preliminary search results indicating a substantial volume of literature, a scoping review methodology was selected. Scoping reviews are particularly well-suited for mapping the extent and characteristics of a specific research landscape.^23^

### 1.3 Goals of This Investigation

The aim of this scoping review was to identify and characterize PROMs used to assess the health status of adult patients in the ED. Specifically, the review focused on PROMs utilized during the initial clinical evaluation to support clinical decision-making and the development of individualized care and treatment plans.

More specifically, the research questions were:

I. Which PROMs have been used to assess the health status of adult patients in the ED setting in the initial evaluation prior to diagnosis?
II. For what purposes have the PROMs been developed and/or applied in the ED?
III. To which patient groups have the PROMs been developed and/or applied?
IV. What is the content of the PROMs?
V. How are the PROMs reported and utilized in the ED?

## 2 Methods

### 2.1 Protocol and Design

This scoping review follows a prespecified protocol, published in 2021,^24^ and adheres to the Preferred Reporting Items for Systematic Reviews and Meta-Analyses (PRISMA) extension for Scoping Reviews (PRISMA-ScR) guideline,^25^ and the Joanna Briggs Institute methodology. No formal quality assessment or evidence synthesis was performed.^26^

### 2.2 Inclusion and Exclusion Criteria

**Figure 1:**
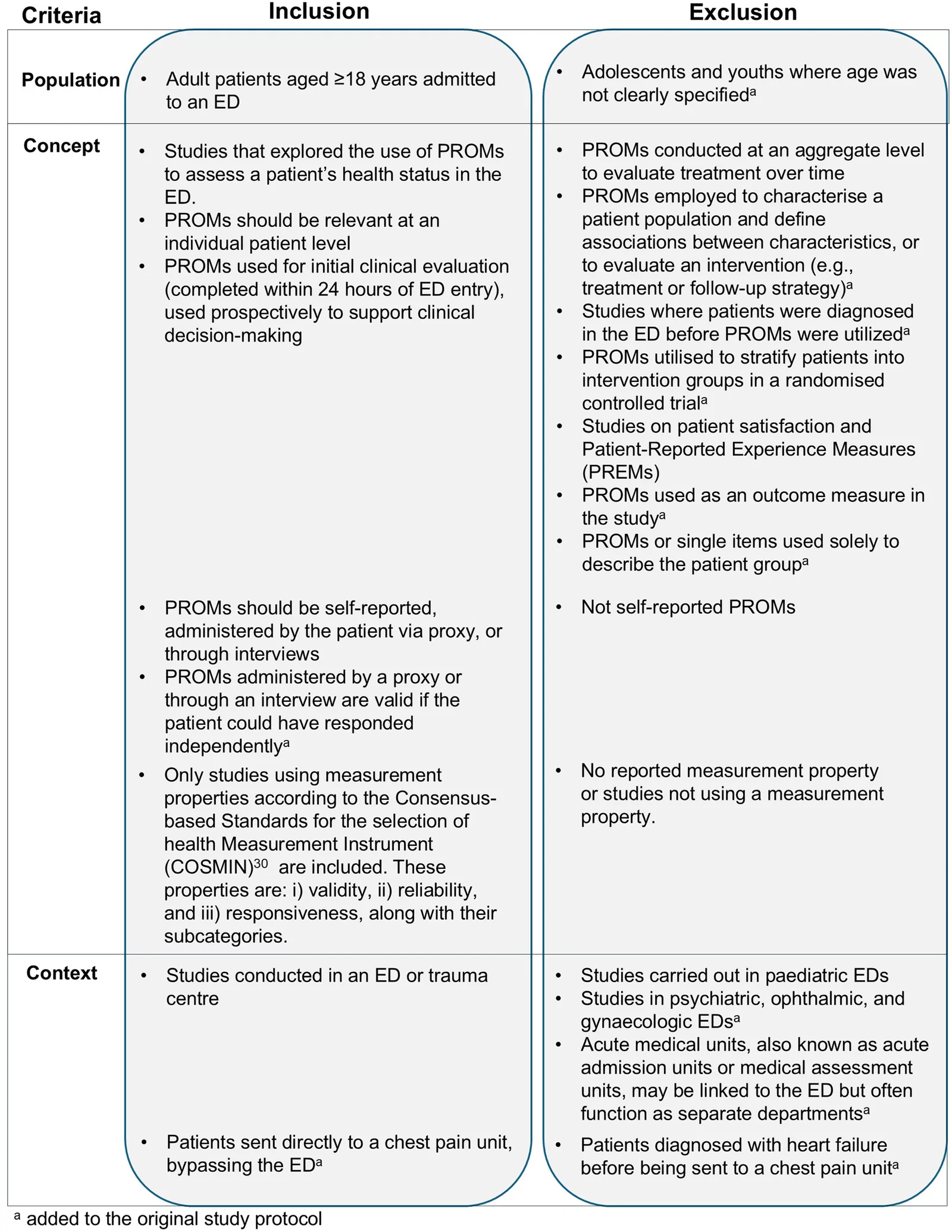
Inclusion and exclusion criteria.

The inclusion criteria were developed using the Population, Concept, and Context framework. Throughout the iterative review process, these criteria were refined and documented. The finalized criteria are depicted in Figure 1. Specifically, this review encompasses studies that utilize PROMs to assess the health status of adult patients during the initial clinical evaluation within the ED setting.

### 2.3 Search Strategy

To address terminological variability and the late introduction of “PROM” as a MeSH term (2017),^27^ an expansive strategy was employed. This included utilizing the COSMIN filter for PROMs,^28^ supplemented by searching for specific individual PROMs, and a COSMIN filter for measurement properties that predates the introduction of the MeSH term.^29^ Additionally, a more targeted search was performed using the MeSH term “Patient-Reported Outcome Measure” and its acronyms. The initial search was conducted in October 2020, with a subsequent update conducted in February 2025. The revised search strategy in Embase (Ovid) is provided in Table S1, whereas the original strategy has been previously documented.^24^ Studies published in Danish, Swedish, Norwegian, German, and English were considered for inclusion due to the authors’ linguistic proficiency in these languages. The search process was conducted in collaboration with an expert research librarian.

The databases searched comprised MEDLINE (Ovid), Embase (Ovid), CINAHL (EBSCOhost), PsycINFO (Ovid), the Cochrane Library (Wiley), and PROSPERO (University of New York). To identify unpublished studies and gray literature, we examined sources, including the National Institute for Health and Care Excellence (NICE), the World Health Organization (WHO), the Centers for Disease Control and Prevention, Google Scholar, and official websites of identified PROMs.

**Figure 2:**
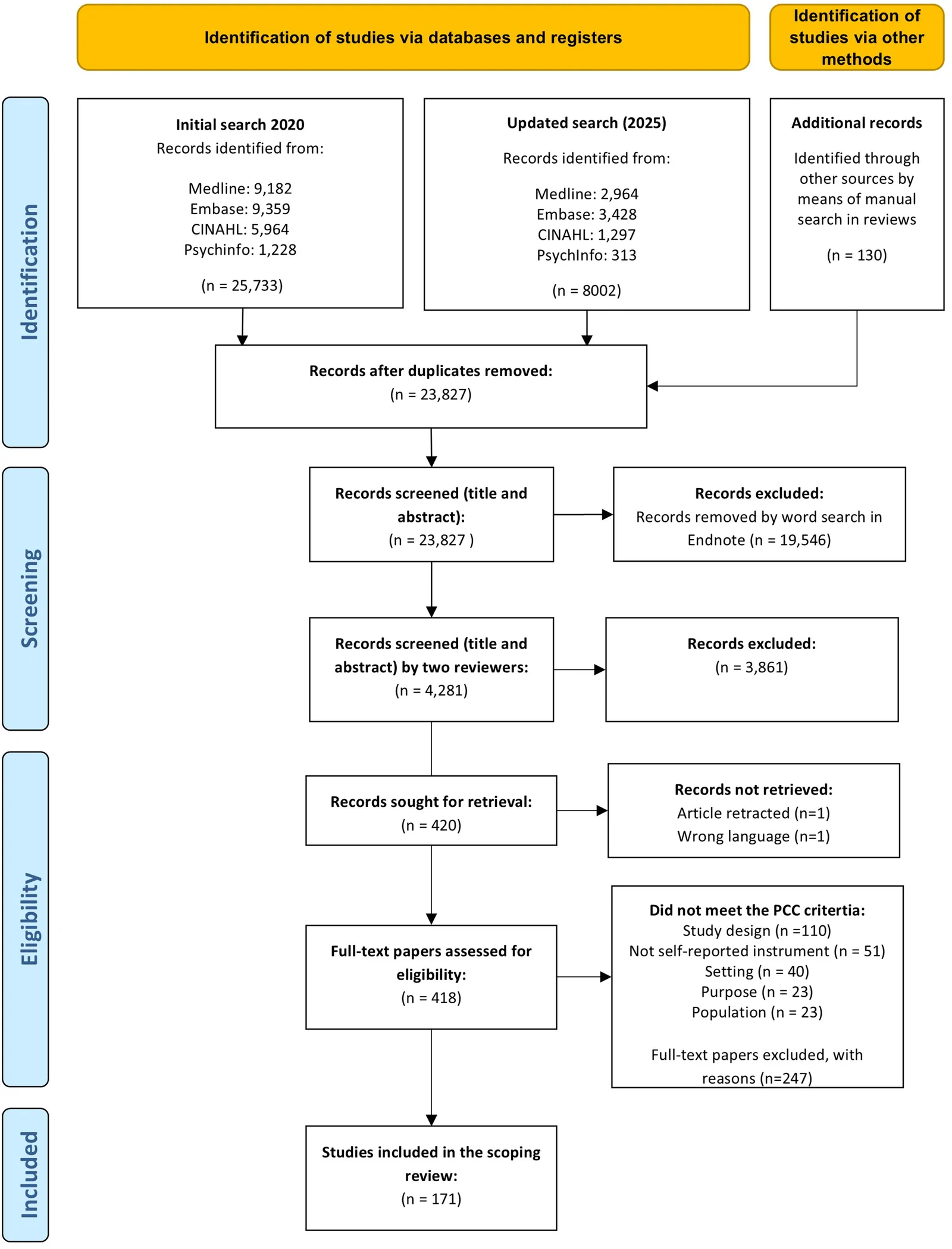
Search results, study selection and inclusion process.

This scoping review included quantitative, qualitative, and mixed-method studies. Feasibility studies were excluded if their sole focus was on feasibility and did not report on any measurement properties, as such studies are not considered a core component of measurement properties according to the COSMIN guidelines.^30^ We omitted book chapters, statements, case studies, conference abstracts, dissertations, opinion papers, case reports, policy statements, protocols, short reports, cost-effectiveness studies, and studies lacking abstracts. To avoid double counting, reviews, guidelines, and overviews were excluded,^31^ extracting only primary studies. Ultimately, 130 primary studies were identified from 65 reviews (Fig 2).

### 2.4 Study Selection

The PRISMA flow diagram was used to illustrate the search decision process.^32^ (Fig 2) All identified records were imported into EndNote 20.0.1 (Clarivate Analytics, PA, USA), and duplicates were removed.^33^ All studies were initially screened by the first author with a word search in EndNote to exclude studies with clear topical irrelevance. Three authors verified a subset of 863 studies (3.6%), with no disagreements observed. Remaining titles and abstracts were screened by 2 reviewers in Covidence following a pilot screening by 4 authors. To manage the high record volume, exclusion criteria were refined to require mention of ED, trauma center, ED patients, trauma patients, or acute patients in the title or abstract. Full texts were retrieved and assessed for eligibility in Covidence. Additionally, all PROMs utilized in these studies were evaluated against the inclusion criteria; specifically PROMs had to be self-reported by the patient (eg., not an evaluation by a proxy but administered by the patient or via interview). Any disagreements were resolved through consensus-based discussion or by involving a third reviewer. A supplementary document was maintained to record all discussions and decisions made throughout the process.^34^

### 2.5 Data Extraction

A pretested data extraction tool was used. Following confirmed concordance between 2 independent reviewers, 1 reviewer performed the primary extraction, whereas a second reviewer conducted random pilot checks on 25% of the data.

Two data extraction forms were employed (Table S2). The first form captured study characteristics (eg, author, title, publication year, country, and journal), study features (eg, aim and study design), context, patient demographics (eg, population and sample size), and the core concept (eg, PROM utilized, reporting method, feedback, and the development or refinement of a PROM). The second form focused on specific details about the PROM used (eg, content and classification of purpose). All data were reviewed by the first author, with ambiguities resolved through discussion.

### 2.6 Synthesis

The results are presented in a tabular form accompanied by a narrative summary. The data analysis was structured according to the research questions. We categorized the identified PROMs into 3 categories: generic, disease-specific, and symptom-specific. Generic PROMs are intended for broad applications regardless of the patient’s specific condition, whereas disease-specific PROMs address issues pertinent to a particular illness. Symptom-specific PROMs focus on assessing symptoms that may manifest across diverse patient groups.^35^ Furthermore, PROMs were classified by topics and subtopics, which emerged through an iterative development process. To determine their purpose, PROMs were categorized into discriminative (distinguishing varying levels of impairment), evaluative (assessing changes over time), or predictive (identifying probable outcomes).^36^

## 3 Results

### 3.1 Study Selection and Characteristics

A total of 4281 studies were screened by 2 reviewers, followed by 418 full text studies (Fig. 2). In all, 171 studies were included in the review. A complete list of references is provided in Supplementary Appendix 3.

**Figure 3:**
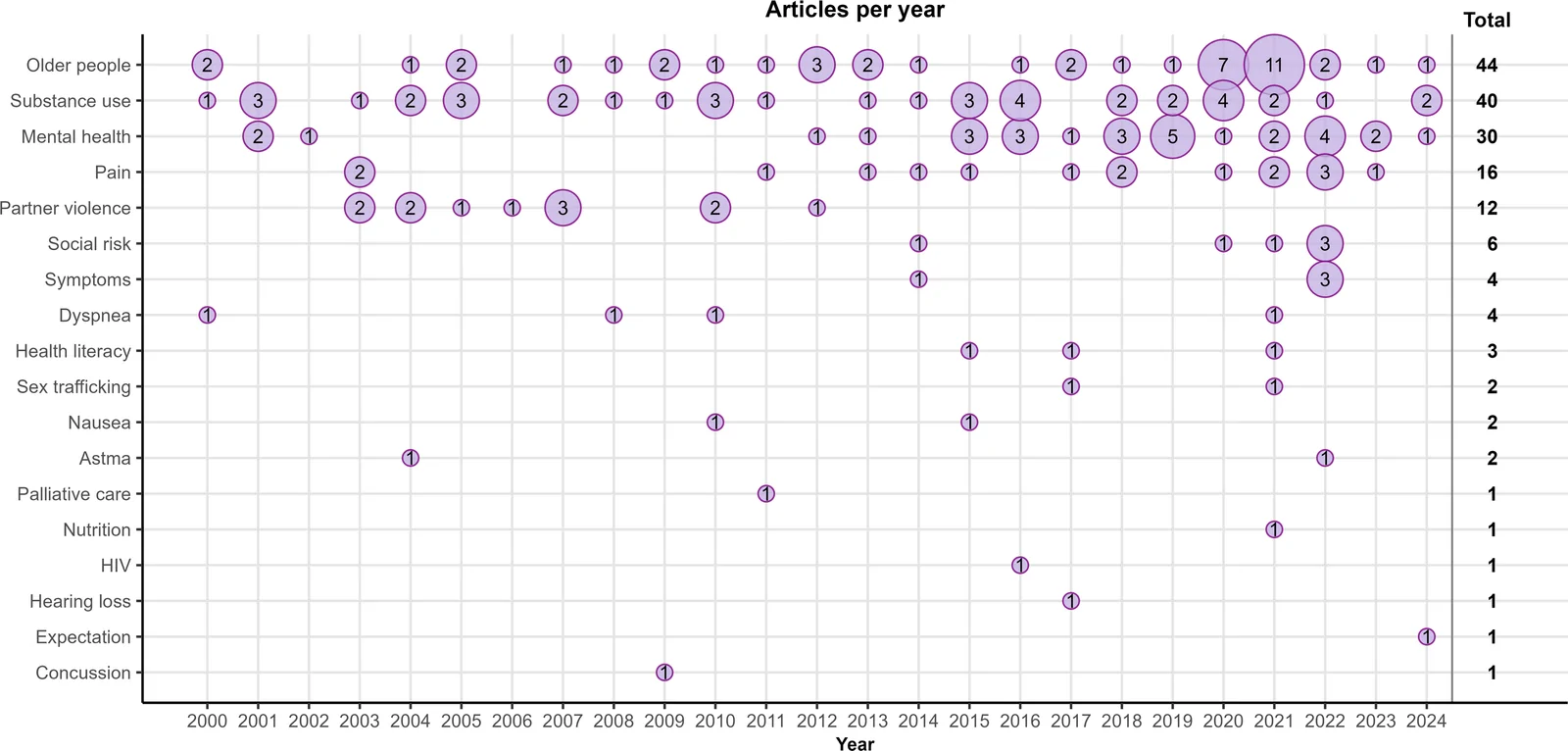
Study theme by year (N = 171).

The studies were published between 2000 and 2024 (Fig 3), with the majority originating from North America (52.0%), followed by Europe (22.2%) (Table 1). The majority of articles employed cohort studies (41.1%) or cross-sectional studies (18.1%), whereas one-fourth of the articles did not specify a study design (Table 1). Other methodologies included test-retest studies,^37^ prognostic,^38^ or pilot studies,^39^ with no explicitly defined design. Regarding measurement properties, validity was most frequently reported (80.7%), followed by both validity and reliability (11.1%) (Table 1). Sample sizes ranged from 16 to 12,983 participants. All studies were categorized by their primary central theme; the most prevalent theme was *older people* (n = 44), followed by *substance use* (n = 40), and *mental health* (n = 30) (Fig 3). Although publications concerning *older people* and s*ubstance use* remained consistently represented throughout the review period, studies related to *mental health* demonstrated a growing trend, with a higher volume of studies emerging from 2015 onwards (Fig 3).

**Table 1:** Characteristics of the reviewed studies (N = 171).

	N = 171, n (%)
Continental reference origin	
North America	89 (52.0)
Europe	38 (22.2)
Asia (incl. Turkey)	26 (15.2)
Australia	8 (4.7)
South America	5 (2.9)
Africa	2 (1.2)
Multiple continents	3 (1.8)
Study design	
Cohort studies	72 (41.1)
Cross-sectional	31 (18.1)
Randomized controlled trial	5 (2.9)
Quasi-experimental	2 (1.2)
Case-control	1 (0.6)
Mixed method	1 (0.6)
Qualitative	1 (0.6)
Other	18 (10.5)
Not described	40 (23.4)
Measurement properties	
Validity	138 (80.7)
Validity and reliability	19 (11.1)
Reliability	11 (6.4)
Validity, reliability and responsiveness	2 (1.2)
Reliability and responsiveness	1 (0.6)

### 3.2 Categorization of PROMs Used in EDs

**Figure 4:**
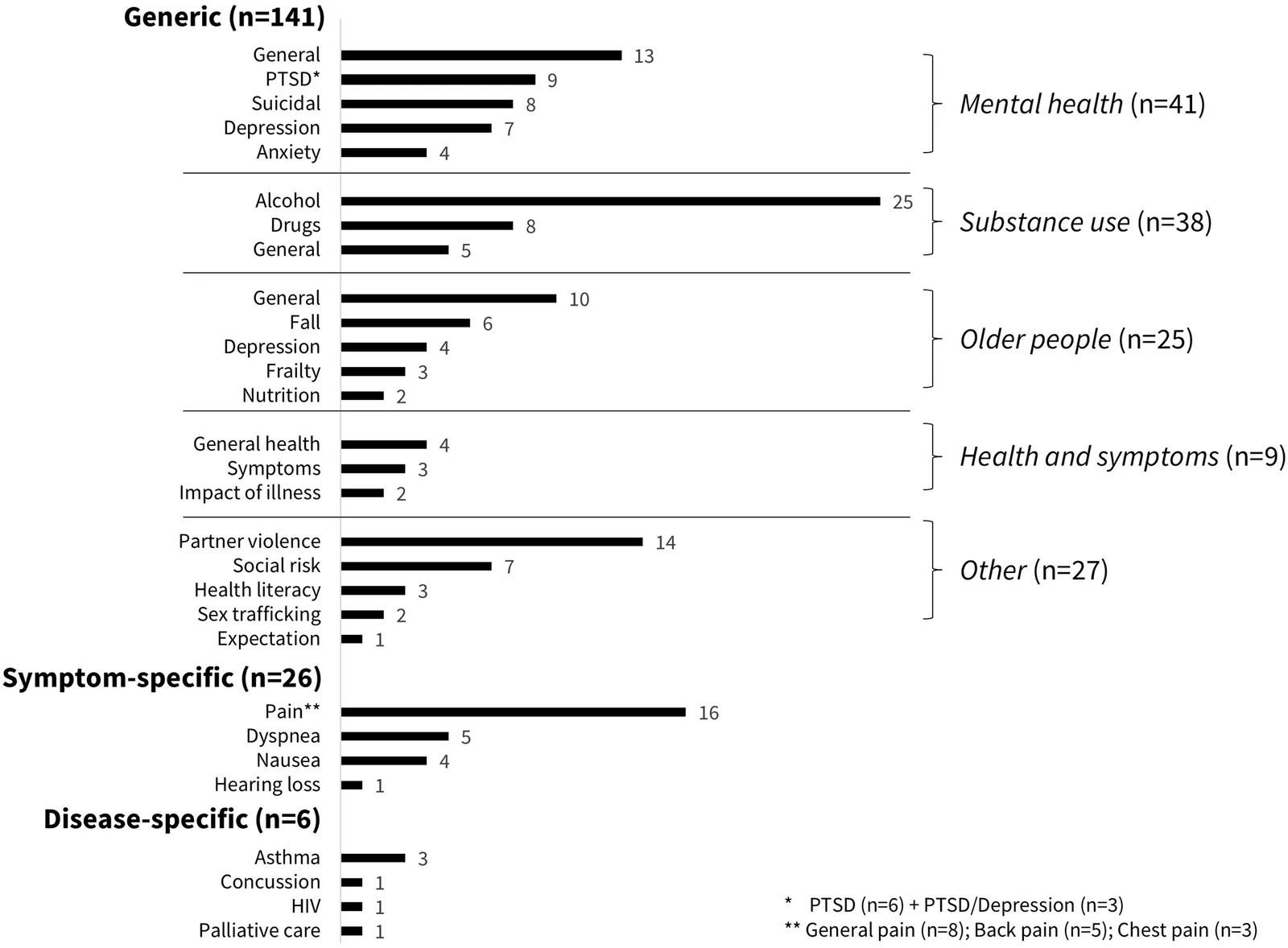
Distribution of the identified PROMs in accordance with topics (N = 173).

A total of 173 PROMs was identified, comprising 153 with distinct titles, and 20 PROMs that lacked a designated title. Additionally, 12 of the identified PROMs were designed for, or were amenable to Computer Adaptive Testing (CAT) methodology (Table S4 for all identified PROMs). The distribution of the identified PROM is depicted in Figure 4, with the majority falling in the generic category. Within the generic category, 9 central topics were identified with the most frequent being *mental health* (n = 41), *substance use* (n = 38), and *older people* (n = 25). Although a large number of generic PROMs were identified, only 9 were specifically designed for the undifferentiated general ED population (covering health and symptoms). Several topics included subtopics, such as *older people*, which encompassed 5 subtopics. The s*ubstance use* topic, specifically the *alcohol* subtopic, represented the greatest variety of PROMs (n = 25) (Fig 4 and Table S4). The application of PROMs across different patient groups is detailed in Table S4.

Twenty-three studies described the development of novel PROMs, specifically designed for use in the ED, resulting in 23 distinct instruments.^38^, ^39^, ^40^, ^41^, ^42^, ^43^, ^44^, ^45^, ^46^, ^47^, ^48^, ^49^, ^50^, ^51^, ^52^, ^53^, ^54^, ^55^, ^56^, ^57^, ^58^, ^59^, ^60^ An additional 22 studies focused on the further refinement of established PROMs.^61^, ^62^, ^63^, ^64^, ^65^, ^66^, ^67^, ^68^, ^69^, ^70^, ^71^, ^72^, ^73^, ^74^, ^75^, ^76^, ^77^, ^78^, ^79^, ^80^, ^81^, ^82^ These efforts included attempts to improve or derive the PROM for the ED,^72^ translation and adaptation of existing tools,^81^ and the exploration of additional items to enhance sensitivity and specificity in an ED context.^80^

### 3.3 Content and Purpose of PROMs

The content and purpose of the identified PROMs are detailed in full in Table S5 and with 3 representative instrument examples from the most frequent topics in Table 2,^20^^,^^37^^,^^56^^,^^70^^,^^74^^,^^83^, ^84^, ^85^, ^86^, ^87^, ^88^, ^89^, ^90^, ^91^, ^92^, ^93^, ^94^, ^95^, ^96^, ^97^, ^98^, ^99^, ^100^, ^101^, ^102^, ^103^, ^104^, ^105^, ^106^, ^107^, ^108^, ^109^, ^110^, ^111^ including *substance use, mental health* and *older people*. The number of items per PROM ranged from a single item^80^ to 53 items.^112^ PROMs utilizing CAT featured item banks, ranged from 4 items^113^ to 1500 items.^114^ Completion time for CAT instruments varied from 1 minute^37^^,^^91^ to 10 to 15 minutes^115^ (Table S5). The most frequently cited PROMs were Identification of Seniors At Risk (ISAR), appearing in 21 studies and Alcohol Use Disorder Identification Test (AUDIT), utilized in 11 studies (Table 2 and Table S5). Finally, the majority of PROMs (n = 117, 67.6%) were employed for discriminative purposes, followed by predictive purposes (n = 24, 13.9%), combined discriminative and predictive purposes (n = 18, 10.4%), combined discriminative and evaluative (n = 12, 6.9%), and purely evaluative purposes (n = 2, 1.2%) (Table 2 and Table S5).

**Table 2:** Examples of characteristics of the included PROM by topic, subtopic, content, purpose, and number of items.

PROM	Topic	Subtopic	Content	Purpose	Number of items	Study (references)
Alcohol use disorder identification test (AUDIT)	Substance use	Alcohol	AUDIT was developed by World Health Organization to identify problem drinkers in primary care settings. AUDIT covers the 3 conceptual domains of consumption levels, dependence symptoms, and alcohol–related consequences.	Discriminative	10	Borges et al^83^ Brousse et al^84^ Cherpitel et al^85^ Cherpitel et al^86^ Chung et al^87^ Cremonte et al^70^ Fulbrook et al^74^ Lee et al^88^ Meneses-Gaya et al^89^ Neumann et al^90^ Shenvi et al^56^
Computerized Adaptive Test-Depression Inventory (CAT-DI)	Mental health	Depression	CAT-DI is a computerized adaptive dimensional severity measure for depression that utilizes a bank of 389 depression items whose response patterns are fitted to a multidimensional item response theory model.	Discriminative	389 The average is 12 items Completion time:2 minutes	Beiser et al^37^ Beiser et al^91^
Identification of Seniors at Risk (ISAR)	Older people	General	ISAR identifies older people (≥65 years) at risk of functional decline, mortality, readmission and institutionalization. ISAR consists of 6 assessment items: presence of home help, increased dependency, history of hospital admissions, visual problems, memory problems, and polypharmacy (≥3 drugs).	Discriminative and predictive	6	Gentile et al^92^ Graf et al^93^ Bahadirli et al^94^ Braes et al^95^ Braes et al^96^ de Almeida Tavares et al^97^ Demir Akça et al^98^ Dendukuri et al^99^ Heeren et al^100^ Loddo et al^101^ McCusker et al^102^ McCusker et al^103^ Moons et al^104^ Rizka et al^105^ Rizka et al^106^ Salvi et al^20^ Salvi et al^107^ Salvi et al^108^ Singler et al^109^ Suffoletto et al^110^ Tavares et al^111^

PROM, patient-reported outcome measures.

### 3.4 PROM Administration, Utilization and Clinical Feedback

In the majority of studies (n = 96; 56.1%), PROMs were administered by the clinician or researcher conducting the patient interview. In 29.2% of the studies (n = 50), PROMs were self-administered by patients. A further 9.4% employed a combined approach, offering patients the option of self-administration with supplementary support from HCP, researchers or next-of-kin when necessary. In 5.3% of the studies (n = 9) the administration method was not disclosed. Regarding clinical application, PROMs were primarily utilized for research purposes (n = 141; 82.5%), with no reported clinical feedback to patients or HCPs. However, in 30 studies (17.5%), PROMs were also integrated into clinical practice, where feedback was provided to HCP and/or patients, intended to enhance patient care.

The diverse methods for utilizing clinical feedback from collected PROMs and their application in clinical practice are specified in Table 3.^39^^,^^40^^,^^43^^,^^47^^,^^49^^,^^51^^,^^54^^,^^64^^,^^70^^,^^88^^,^^116^, ^117^, ^118^, ^119^, ^120^, ^121^, ^122^, ^123^, ^124^, ^125^, ^126^, ^127^, ^128^, ^129^, ^130^, ^131^, ^132^ Based on the utilization of feedback, the content was categorized into 4 distinct types—ED care and treatment, referral to other departments/clinics, community referrals, and mental health referrals. The most frequent application involved consultation with an ED social worker for patient assessment (n = 5). Among studies employing feedback, mental health (n = 10) was the most common, followed by substance use (n = 7), and partner violence (n = 5) (Table 3).

**Table 3:** PROMs used with clinical feedback.

Study theme *Name of PROM* (topic in bracket)	Feedback	Author (references)
ED care and treatment		
Expectation ***Expectation questionnaire***	Clinicians could respond as they felt appropriate to the patient’s questionnaire on their expectations for the ED visit.	Chapman et al^43^
HIV ***HIV RE&IC***	All people with HIV positive results were informed and counseled about the result.	Pérez Elías et al^54^
Mental Health ***PERC screener*** (Suicide) Mental Health ***PSS*** (Suicide) Mental Health ***CAT-MH*** (General)	Treating physician/clinician were informed about the positive screening results and patients were managed by usual care.	Allen et al^39^ Boudreaux et al^64^ O'Reilly et al^114^
Mental Health ***"Short two questions scale"*** (Depression) Sex trafficking ***Human trafficking screening tool*** Sex trafficking ***Screening survey*** Partner violence ***PVS***	Patients referred to an ED social worker.	Haughey et al^116^ Kalitso et al^77^ Mumma et al^51^ Trautman et al^117^
Mental Health ***PRIME-MD*** (General)	Patients referred to an ED psychiatric social worker.	Schriger et al^118^
Mental Health ***NIDA, PHQ-2, GAD-2, HARK, HITS*** (General)	Patients were encouraged to discuss affirmative results (depression, anxiety, alcohol use, drug use, sleep, IPV, and chronic pain) with their ED care team. ED physician was notified, and the care team addressed the concerns based on clinical judgment.	Kene et al^119^
Substance Use ***AUDIT*** (Alcohol)	Patients were informed about the AUDIT score.	Lee et al^88^
**Referral to other departments/clinics**		
Older people ***BRIGHT*** (General)	Older people with significant identified needs were referred to appropriate interdisciplinary and multidisciplinary team for further assessment and intervention.	Boyd et al^120^
Substance use ***AUDIT, CAGE, BMAST, RAPS-4, RAPS-QF, TWEAK*** (Alcohol)	Participants with an alcohol–related problem were referred to the hospital’s Alcoholism and Liver Department and to self-help groups.	Cremonte et al^70^
Substance use ***NIDA*** (General)	A positive response to at-risk substance use in pregnancy, followed a targeted brief intervention and education on risks. Individuals reporting use of drugs were either referred to a specialized clinic or evaluated immediately in an emergency care setting by a specialized multidisciplinary team.	White et al^121^
**Community referrals**		
Nutrition ***HVS, MST***	Patients screening positive for food insecurity or malnutrition received a handout, providing contact information for local community resources.	Aylward et al^122^
Partner violence ***OVAT***	Following data collection about IPV, patients were provided with information regarding local referral agencies as needed or upon request. If participants expressed an immediate need for assistance, this was documented and social services were engaged, in accordance with mandatory reporting requirements.	Ernst et al^123^
Partner Violence ***SAFE-T***	All interested patients received 5-10 minutes of verbal education about intimate partner violence after completing the surveys. Each patient was offered an emergency packing list with a confidential contact number. Those disclosing partner violence and requesting support were referred to an ED clinician or local shelter.	Fulfer et al^47^
Partner violence ***PVS***	Patients exposed to partner violence were offered resources on domestic violence. ED physician was notified, due to mandatory reporting.	Houry et al^124^
Social risk ***SINCERE***	All patients were asked if they wanted to be contacted by an information specialist. All patients were introduced to community resources (free services providing referrals to low- and no-cost community resources for needs such as transportation, food, housing, and medications)	Guo et al^125^
Substance use ***CASI*** (Alcohol)	Patients with an alcohol consumption above the recommended received a personalized alcohol-reduction plan and local counseling resources within their community.	Lotifipour et al^126^
**Mental health referrals**		
Mental Health ***ITSS, PDI*** (PTSD and depression)	Patients exhibiting signs of depression or PTSD were referred to mental health treatment.	Ennis et al^127^
Mental Health ***C-SSRS*** (Suicide)	Patients with moderate to high risk of suicide were referred for specialty mental health treatment; patients with low risk received further risk assessment by either an emergency medicine or a mental health clinician to determine the need for further psychiatric intervention.	Simpson et al^128^
Mental Health ***CARS*** (General)	Patients received their PROM report (identifies up to 16 major psychiatric disorders), as well as a list of mental health resources tailored to their local area of residency, before being discharged from the ED. All patients had time to bring up their screening results with their ED physicians.	Thompson et al^115^
Older people ***GDS-15*** (Depression)	Patients who screened positive for depression were counseled to attend psychiatry clinic at the hospital.	Shrestha et al^129^
Partner violence ***Brief mental health screen, BDI-II, BSS, PDS, UVPSP***	Women disclosing moderate to severe depressive or PTSD symptoms were provided with local mental health referrals. Those reporting suicidal ideation underwent immediate medical clearance in the ED followed by transfer to psychiatric emergency services.	Houry et al^49^
Substance use ***TICS, ASSIST*** (General)	Patients identified as at risk for alcohol and other drug (AOD) during the initial screening were offered more comprehensive assessment. Subsequent options included eg, discharge to an inpatient psychiatric unit, referral for AOD treatment, consultation with a mental health or AOD practitioner, or integrated care. Information regarding harmful substance use was provided to approximately half of the patients.	McCabe et al^130^
Substance use ***ASSIST*** (General) Mental health ***SRQ*** (General)	In both studies, if patients required referral for mental health or social services, it was provided. Urgent attention was handled by emergency care medical staff.	van der Westhuizen^131^ van der Westhuizen^132^
Substance use ***DARSSA*** (General)	The report generator produced 3 reports using the data collected through the assessment: (i) the Health Care Provider Report, (ii) the Patient Feedback Report, and (iii the Referral Provider Report.	Boudreaux et al ^40^

ASSIST, alcohol, smoking and substance involvement screening test; AUDIT, alcohol use disorders identification test; BDI-II, Beck Depression Inventory-II; BMAST, brief Michigan Alcohol Screening Test; BRIGHT, brief risk identification for geriatric health tool; BSS, Beck Scale for Suicide Ideation; CAGE, cut down, annoyed, guilty, eye-opener; CARS, computerized assessment and referral system; CASI, computerized alcohol screening and brief intervention; CAT-MH, computer adaptive test – mental health; C-SSRS, Columbia Suicide Severity Rating Scale; DARSSA, dynamic assessment and referral system for substance abuse; GAD-2, generalized anxiety disorder questionnaire (2-item); GDS-15, Geriatric Depression Scale (15-item); HARK, humiliation, afraid, rape, kick; HITS, hurt, insulted, threatened with harm, screamed at; HVS, hunger vital sign; ITSS, injured trauma survivor screen; MST, malnutrition screening tool; NIDA, National Institute on Drug Abuse Quick Screen Questionnaire; OVAT, ongoing violence assessment tool; PDI, peritraumatic distress inventory; PDS, posttraumatic stress diagnostic scale; PERC screener, Psychiatric Emergency Research Collaboration; PHQ-2, Patient Health Questionnaire (2-item); PRIME-MD, primary care evaluation of mental disorders; PSS, patient safety screener; PVS, partner violence screen; RAPS-4, rapid alcohol problems screen; RAPS-QF, rapid alcohol problems screen – quantity frequency; SINCERE, screener for intensifying community referrals for health; SRQ, self-reporting questionnaire; TICS, two-item conjoint screen for alcohol and other drug problems; TWEAK, tolerance, worried, eye-opener, amnesia, K/cut down; UVPSP, universal violence prevention screening protocol.

Levels of feedback ranged from simply informing patients of their PROM score (eg, an AUDIT score^88^) to more comprehensive implementations by Boudreaux et al^40^ regarding *substance use*. The collected data generated 3 distinct report types: (1) the Health care Provider Report, (2) Patient Feedback Report, and (3) Referral Provider Report^40^ (Table 3). This structured approach facilitates the identification of substance abuse and enables targeted referrals that might not be initiated in clinical practice.

Selecting appropriate PROMs for the ED population presents challenges due to the diversity of clinical needs. This scoping review provides a comprehensive overview of PROMs that may be utilized, adapted, or validated for individual clinical application within the ED setting. To facilitate ease of navigation, Box 1 serves as a practical guide for utilizing this review as a clinical reference tool.

Box 1 **How to use this reference tool**

Step 1: Select the clinical area of interest (eg, asthma or substance use).

Step 2: Identify the corresponding PROMs in Table S4.

Step 3: Consult Table 2 (or Table S5) for detailed information regarding the content and intended purpose of the identified instruments.

Step 4: Assess whether the PROM has been used with clinical feedback in Table 3.

Step 5: Evaluate the psychometric properties of the selected PROMs prior to clinical implementation.

## 4 Limitations

Given the challenges associated with indexing PROMs, exacerbated by the variability in terminology used (eg, “instruments”,”questionnaires”), a broad search approach was adopted. Although manual screening process carries an inherent risk of inadvertently excluding relevant studies, the stringent exclusion criteria outlined by the COSMIN methodology,^29^ were not applied to avoid unduly restricting the review’s scope. Furthermore, due to the vast volume of literature, a snowball search (ie, reviewing reference lists or forward citations), was not conducted. The inclusion criteria were restricted to studies explicitly mentioning specific populations, such as ED patients or trauma patients, within the title or abstract; consequently, relevant studies where ED context was not explicitly stated may have been excluded. Additionally, we opted to exclude feasibility studies. We recognize, however, that feasibility is a fundamental prerequisite. If an instrument is not viable for implementation within a clinical setting, assessments of other measurement properties become largely irrelevant. Recently, there have been calls to reconceptualize the validation of PROMs as an ongoing, iterative process of evidence accumulation, with consideration of how the data will be used to make decisions about individuals and populations, instead of a static designation that a PROM is valid.^133^ Finally, in accordance with the scoping review methodology, a formal quality assessment of the included studies and PROMs was not performed,^134^ a factor that should be considered when utilizing these instruments.

## 5 Discussion

PROMs can be implemented at different time-points in the ED, ranging from initial evaluation to post-ED care^16^ This scoping review has mapped 173 PROMs utilized during the initial evaluation phase in the ED setting, offering a valuable resource for researchers, HCPs, and policymakers dedicated to enhancing the quality and patient-centeredness of emergency care. Notably, we identified 23 PROMs specifically developed for the ED, alongside 22 studies focused on adapting established instruments for this unique clinical environment.

Our findings reveal a significant concentration of PROMs focused on vulnerable populations, a distribution that likely reflect both global health care challenges and evolving research trends in clinical and emergency medicine research. Notably, studies addressing older populations were consistently represented throughout the study period, with the ISAR being the most frequently utilized instrument. This trend aligns with the global demographic shift toward an aging population, which necessitates continuous adaptations in healthcare delivery.^135^ Regarding the topic mental health, the number of publications has increased since 2015, with this theme emerging as the most prevalent area in our review. This trend may be linked to the launch of the WHO Mental Health Action Plan in 2013, which emphasized the need for universal access to mental health care^136^ and subsequently catalyzed increased research attention within the ED context. In contrast, substance use remained consistently represented throughout the study period, with AUDIT being the most frequently used instrument. This prevalence likely reflects the significant global public health burden associated with substance use,^137^ where the necessity for targeted prevention, intervention, and policy has likely driven the proliferation of screening measures.

However, a notable misalignment exists between the availability of PROMs and the broader clinical workload of the ED. Although mental health and substance use are critical priority areas, they represent specific subsets of the patient population; for instance, mental health presentations account for only a small percentage of total ED visits in the US,^138^ although they may be underrepresented in existing datasets. Conversely, there remains a relative scarcity of PROMs designed for general medical conditions, which constitute the vast majority of ED volume.^138^^,^^139^ Our study design specifically focused on PROMs intended for use during the initial evaluation phase prior to definitive diagnosis, consequently PROMs utilized after a diagnosis had been established were excluded. This exclusion might have included PROMs used for general medical conditions. Nevertheless, this gap suggests that a significant segment of the ED population may lack access to standardized, validated PROMs capable of capturing PROM during routine, undifferentiated medical encounters. It should be noted, however, that PROMs for the general ED population following treatment and for post-ED care have been developed, such as PROM-ED.^140^

Majority of the studies reported PROM findings at an aggregate level, with limited focus on providing individual patient feedback. Only 17.4% of the studies described how patients were informed of their PROM results highlighting a potential lack of transparency in integration of PROMs into clinical practice. This prevalence may be partially attributed to our inclusion criteria, which required studies to utilize measurement properties. Nevertheless, the identified PROMs possess significant potential for the individual-level relevance, as they can inform both patients and HCP regarding an individual’s health status. However, the utility lacks formal validation at individual level, highlighting a critical gap in the current evidence base. Notably, 30 studies reported using PROMs to provide clinical feedback, most of which involved referrals to other departments. This aligns with findings by Dainty et al,^141^ who observed that ED physicians perceive their primary role as assessing symptom severity to facilitate appropriate treatment or referral. Feedback-oriented studies primarily addressed the topics mental health, substance use, and partner violence; areas where timely intervention for vulnerable populations is paramount, since failure to act upon PROM measurements can have serious consequences. Ensuring that patients are informed about their PROM result is essential for the clinical utility at the individual level, as it fosters patient engagement and supports shared decision-making^10^ To bridge this potential translational gap, future research must shift its focus from PROM development toward implementation science.^16^^,^^142^

In conclusion, this scoping review identified 173 unique PROMs, within the literature reporting on measurement properties, providing a comprehensive overview of instruments utilized during the initial evaluation in the ED setting. We identified 23 PROMs specifically developed for the ED setting and 22 studies dedicated to adapting on established instruments for this environment. The most frequently addressed themes were mental health, substance use, and older people. Although the majority of identified PROMs were generic and employed for discriminative purposes, the relative scarcity of instruments designed for general medical conditions suggests a significant segment of the ED population may lack access to standardized validated PROMs during initial evaluation. Furthermore, the current predominance of research-focused use of PROMs underscores an opportunity to better translate PROMs into routine clinical practice, thereby supporting real-time clinical decision-making and individualized care planning.

## Author Contributions

NRHD, MKP, LMVS, MBS, PDCL conceptualized and designed the study. NRHD, MKP, LMVS, MBS, IML, KBØ, MTM and PDCL performed the title and abstract screening, the full text screening and the data extraction. NRHD interpreted the data and drafted the manuscript. All authors critically edited, reviewed, and approved the manuscript.

## Funding and Support

By *JACEP Open* policy, all authors are required to disclose any and all commercial, financial, and other relationships in any way related to the subject of this article as per ICMJE conflict of interest guidelines (see www.icmje.org). This work was supported by funding from the Health Science Research Foundation of North Denmark Region (Region Nordjyllands Sundhedsvidenskabelige Forskningsfond), and North Denmark Regional Health Innovations Foundation.

## Declaration of Generative AI and AI-Assisted Technologies in the Writing Process

During the preparation of this work, the authors used Grammarly 2026 for minor language refinement, including grammar and clarity. After using this tool the authors reviewed and edited the content as needed and take full responsibility for the content of the publication.

## Data Sharing Statement

Partial or complete datasets are available upon request, from the date of article publication by contacting Ninna R. H Daugaard at nrp.dcm@aau.dk.

## Conflict of Interest

All authors have affirmed they have no conflicts of interest to declare.

## Supplementary Materials

Supplementary Table 5

Supplementary Appendix Index
