# Supplementary material for: Patient-Reported Outcome Measures in the Emergency Department: A Scoping Review
Source: J Am Coll Emerg Physicians Open. 2026 Aug 17;7(5):100486. doi: 10.1016/j.acepjo.2026.100486 (PMC13520099; doi:10.1016/j.acepjo.2026.100486)
Supplement: Supplementary file 1 [file mmc1.pdf]

Table S1: Search strategy

Embase (Ovid) search conducted February 2025.

#	Query	Results
1	emergency ward/ or emergency nursing/	251,394
2	hospital emergency service/	11,328
3	((emergency or acute or trauma) adj2 (ward* or department* or unit* or center* or room*)).ti,ab.	312,411
4	1 or 2 or 3	379,422
5	(HRPRO or ((patient or self) adj (report or reported or reporting or rated or rating* or assessed or assessment* or administer*)) or (self adj screen*)).ti,ab,kw.	532,647
6	patient-reported outcome/ or (prom or pro or proms).ti,ab,kw.	489,133
7	5 or 6	959,743
8	(unreliab* or homogeneity or homogeneous).ti,ab. or intermethod comparison/ or exp data collection method/ or validation study/ or feasibility study/ or pilot study/ or exp psychometry/ or reproducibility/ or reproducib*.ab,ti. or "audit".ab,ti. or psychometr*.ab,ti. or clinimetr*.ab,ti. or clinometr*.ab,ti. or observer variation/ or "observer variation".ab,ti. or exp discriminant analysis/ or exp validity/ or reliab*.ab,ti. or valid*.ab,ti. or "coefficient".ab,ti. or "internal consistency".ab,ti. or (cronbach* and ("alpha" or "alphas")).ab,ti. or "item correlation".ab,ti. or "item correlations".ab,ti. or "item selection".ab,ti. or "item selections".ab,ti. or "item reduction".ab,ti. or "item reductions".ab,ti. or "agreement".ab,ti. or "precision".ab,ti. or "imprecision".ab,ti. or "precise values".ab,ti. or "test-retest".ab,ti. or ("test" and "retest").ab,ti. or (reliab* and ("test" or "retest")).ab,ti. or "stability".ab,ti. or "interrater".ab,ti. or "inter-rater".ab,ti. or "intrarater".ab,ti. or "intra-rater".ab,ti. or "intertester".ab,ti. or "inter-tester".ab,ti. or "intratester".ab,ti. or "intratester".ab,ti. or "interobeserver".ab,ti. or "inter-observer".ab,ti. or "intraobserver".ab,ti. or "intraobserver".ab,ti. or "intertechnician".ab,ti. or "inter-technician".ab,ti. or "intratechnician".ab,ti. or "intratechnician".ab,ti. or "interexaminer".ab,ti. or "inter-examiner".ab,ti. or "intraexaminer".ab,ti. or "intraexaminer".ab,ti. or "interassay".ab,ti. or "inter-assay".ab,ti. or "intraassay".ab,ti. or "intra-assay".ab,ti. or "interindividual".ab,ti. or "inter-individual".ab,ti. or "intraindividual".ab,ti. or "intra-individual".ab,ti. or "interparticipant".ab,ti. or "inter-participant".ab,ti. or "intraparticipant".ab,ti. or "intraparticipant".ab,ti. or "kappa".ab,ti. or "kappas".ab,ti. or "coefficient of variation".ab,ti. or repeatab*.ab,ti. or ((replicab* or "repeated") and ("measure" or "measures" or "findings" or "result" or "results" or "test" or "tests")).ab,ti. or generaliza*.ab,ti. or generalisa*.ab,ti. or "concordance".ab,ti. or ("intraclass" and	8,774,128

	correlation*).ab,ti. or "discriminative".ab,ti. or "known group".ab,ti. or "factor analysis".ab,ti. or "factor analyses".ab,ti. or "factor structure".ab,ti. or "factor structures".ab,ti. or "dimensionality".ab,ti. or subscale*.ab,ti. or "multitrait scaling analysis".ab,ti. or "multitrait scaling analyses".ab,ti. or "item discriminant".ab,ti. or "interscale correlation".ab,ti. or "interscale correlations".ab,ti. or (("error" or "errors") and (measure* or correlat* or evaluat* or "accuracy" or "accurate" or "precision" or "mean")).ab,ti. or "individual variability".ab,ti. or "interval variability".ab,ti. or "rate variability".ab,ti. or "variability analysis".ab,ti. or ("uncertainty" and ("measurement" or "measuring")).ab,ti. or "standard error of measurement".ab,ti. or sensitiv*.ab,ti. or responsive*.ab,ti. or ("limit" and "detection").ab,ti. or "minimal detectable concentration".ab,ti. or interpretab*.ab,ti. or (small* and ("real" or "detectable") and ("change" or "difference")).ab,ti. or "meaningful change".ab,ti. or "minimal important change".ab,ti. or "minimal important difference".ab,ti. or "minimally important change".ab,ti. or "minimally important difference".ab,ti. or "minimal detectable change".ab,ti. or "minimal detectable difference".ab,ti. or "minimally detectable change".ab,ti. or "minimally detectable difference".ab,ti. or "minimal real change".ab,ti. or "minimal real difference".ab,ti. or "minimally real change".ab,ti. or "minimally real difference".ab,ti. or "ceiling effect".ab,ti. or "floor effect".ab,ti. or "item response model".ab,ti. or "irt".ab,ti. or "rasch".ab,ti. or "differential item functioning".ab,ti. or "dif".ab,ti. or "computer adaptive testing".ab,ti. or "item bank".ab,ti. or "cross-cultural equivalence".ab,ti.	
9	4 and 7 and 8	5,567
10	patient-reported outcome/ or (prom or proms or pros).ti,ab,kw.	97,267
11	4 and 10	1,488
12	9 or 11	6,376
13	12 not conference abstract/	4,900
14	limit 13 to dc=20220913-20250227	1,124
