# Supplementary material for: Patient-Reported Outcome Measures in the Emergency Department: A Scoping Review
Source: J Am Coll Emerg Physicians Open. 2026 Aug 17;7(5):100486. doi: 10.1016/j.acepjo.2026.100486 (PMC13520099; doi:10.1016/j.acepjo.2026.100486)
Supplement: Supplementary file 2 [file mmc2.pdf]

Table S2: Data extraction instrument

First data extraction form: Study information

**Data extractions**	
Date of data extraction	
**Study characteristics**	
Author(s)	
Year of publication	
Journal	
Origin/country of origin	
Article title	
**Study features**	
Aim/purpose	
Study design, method(s) and outcome measures	
Measurement property	
**Context**	
Clinical setting	
**Population**	
Population and sample size	
Diagnostic group; developed and applied to	
**Concept**	
PROM used in the article	
Purpose of the PROM	
Development of a new PROM or further development of known PROM	

Reporting method (e.g. self-reported or interview)	
Feedback (feedback recipients)	
Content of feedback	

Second data extraction form: PROM used in the study

**PROM name**	
Categorization of PROM (Topic, subtopic)	
Content of the PROM	
Classification of purpose	
Number of items in PROM	
Expected time to fill in the PROM	
Summed into a score?	
