# Supplementary material for: Patient-Reported Outcome Measures in the Emergency Department: A Scoping Review
Source: J Am Coll Emerg Physicians Open. 2026 Aug 17;7(5):100486. doi: 10.1016/j.acepjo.2026.100486 (PMC13520099; doi:10.1016/j.acepjo.2026.100486)
Supplement: Supplementary file 3 [file mmc3.pdf]

Appendix 3 All the included studies in the scoping review

1. Ahmed A, Bushra A, Nisar N, et al. Adapting Health Literacy Tool for Use in Hospital: Experience of Holy Family Hospital Rawalpindi, Pakistan. Medical Forum Monthly 2021;32(10).
2. Airagnes G, Le Faou AL, Duviviers E, et al. Does a standardized screening for alcohol use disorder at the Emergency Department inform the risk of repeated ED visit? Gen Hosp Psychiatry 2021;69:115–116.
3. Al-Kalaldeh M, Al-Zaidaneen H. Congruency of nurse–patient pain scoring in the emergency department: a cross-sectional observational study. Pain Management 2022;12(5):635–643.
4. Allen MH, Abar BW, McCormick M, et al. Screening for suicidal ideation and attempts among emergency department medical patients: instrument and results from the Psychiatric Emergency Research Collaboration. Suicide Life Threat Behav 2013;43(3):313–23.
5. Aylward AF, Engelberg Anderson J, Morris A, et al. Using malnutrition and food insecurity screening to identify broader health-related social needs amongst older adults receiving emergency department care in the Southeastern United States: A cross-sectional study. Health Soc Care Community 2021;29(6):e420–e430.
6. Azizoddin DR, Beck M, Flowers KM, et al. Psychological Evaluation of Patients With Cancer Presenting to the Emergency Department With Pain: Independent Predictors of Worse Pain Severity, Interference, and Higher Hourly Opioid Administration. JCO Oncol Pract 2022;18(10).
7. Bae SJ, Kim E, Lee JH. Validation of the screening test for at-risk drinking in an emergency department using a tablet computer. Drug Alcohol Depend 2022;230:109181.
8. Bahadirli S, Kurt E, Rohat A, et al. Evaluation and comparison of screening tools used to predict the adverse outcomes of elderly patients in the emergency department. Acta Med Mediter 2021;37:1133–39.
9. Bahreini M, Jalili M, Moradi-Lakeh M. A comparison of three self-report pain scales in adults with acute pain. J Emerg Med 2015;48(1):10–8.
10. Beauchet O, Galery K, Vilcocq C, et al. PRISMA-7 and risk for short-term adverse events in older patients visiting the emergency department: results of a large observational and prospective cohort study. The Journal of nutrition, health and aging 2021;25(1):94–99.
11. Beiser D, Vu M, Gibbons R. Test-Retest Reliability of a Computerized Adaptive Depression Screener. Psychiatr Serv 2016;67(9):1039–41.
12. Beiser DG, Ward CE, Vu M, et al. Depression in Emergency Department Patients and Association With Health Care Utilization. Acad Emerg Med 2019;26(8):878–888.
13. Bijur PE, Latimer CT, Gallagher EJ. Validation of a verbally administered numerical rating scale of acute pain for use in the emergency department. Academic emergency medicine 2003;10(4):390–392.
14. Birmes P, Carreras D, Charlet J-P, et al. Peritraumatic dissociation and posttraumatic stress disorder in victims of violent assault. The Journal of nervous and mental disease 2001;189(11):796–798.
15. Bonaccorsi G, Grazzini M, Pieri L, et al. Assessment of Health Literacy and validation of single-item literacy screener (SILS) in a sample of Italian people. Ann Ist Super Sanita 2017;53(3):205–212.
16. Borges G, Cherpitel CJ. Selection of screening items for alcohol abuse and alcohol dependence among Mexicans and Mexican Americans in the emergency department. J Stud Alcohol 2001;62(3):277–85.
17. Boucher V, Lamontagne ME, Lee J, et al. Self-assessment of functional status in older emergency department patients: a cross-over randomized pilot trial. CJEM 2021;23(3):337–341.

18. Boudreaux ED, Bedek KL, Gilles D, et al. The Dynamic Assessment and Referral System for Substance Abuse (DARSSA): development, functionality, and end-user satisfaction. Drug Alcohol Depend 2009;99(1-3):37–46.
19. Boudreaux ED, De Beurs DP, Nguyen TH, et al. Applying Computer Adaptive Testing Methods to Suicide Risk Screening in the Emergency Department. Suicide Life Threat Behav 2019;49(4):917–927.
20. Boudreaux ED, Jaques ML, Brady KM, et al. The patient safety screener: validation of a brief suicide risk screener for emergency department settings. Archives of suicide research 2015;19(2):151–160.
21. Boyd M, Koziol-McLain J, Yates K, et al. Emergency department case-finding for high-risk older adults: the Brief Risk Identification for Geriatric Health Tool (BRIGHT). Acad Emerg Med 2008;15(7):598–606.
22. Boyer L, Baumstarck K, Iordanova T, et al. A poverty-related quality of life questionnaire can help to detect health inequalities in emergency departments. J Clin Epidemiol 2014;67(3):285–95.
23. Braes T, Flamaing J, Sterckx W, et al. Predicting the risk of functional decline in older patients admitted to the hospital: a comparison of three screening instruments. Age and ageing 2009;38(5):600–603.
24. Braes T, Moons P, Lipkens P, et al. Screening for risk of unplanned readmission in older patients admitted to hospital: predictive accuracy of three instruments. Aging clinical and experimental research 2010;22(4):345–351.
25. Broderick KB, Richmond MK, Fagan J, et al. Pilot Validation of a Brief Screen Tool for Substance Use Detection in Emergency Care. J Emerg Med 2015;49(3):369–74.
26. Brousse G, Arnaud B, Geneste J, et al. How CAGE, RAPS4-QF, and AUDIT Can Help Practitioners for Patients Admitted with Acute Alcohol Intoxication in Emergency Departments? Front Psychiatry 2014;5:72.
27. Brucker K, Duggan C, Niezer J, et al. Assessing Risk of Future Suicidality in Emergency Department Patients. Acad Emerg Med 2019;26(4):376–383.
28. Byrne T, Hoang M, Montgomery AE, et al. Performance of 2 Single-Item Screening Questions to Identify Future Homelessness Among Emergency Department Patients. JAMA Netw Open 2022;5(8):e2226691.
29. Canagasaby A, Vinson DC. Screening for hazardous or harmful drinking using one or two quantity-frequency questions. Alcohol Alcohol 2005;40(3):208–13.
30. Chalmers CE, Mullinax S, Brennan J, et al. Screening Tools Validated in the Outpatient Pain Management Setting Poorly Predict Opioid Misuse in the Emergency Department: A Pilot Study. J Emerg Med 2019;56(6):601–610.
31. Chan C, Chan Y, Au A, et al. Reliability and validity of the "Extended-Hurt, Insult, Threaten, Scream"(E-Hits) screening tool in detecting intimate partner violence in hospital emergency departments in Hong Kong. Hong Kong Journal of Emergency Medicine 2010;17(2):109–117.
32. Chang BP, Tan TM. Suicide screening tools and their association with near-term adverse events in the ED. Am J Emerg Med 2015;33(11):1680–1683.
33. Chapman J, Wakely M, Leonard K, et al. Great expectations: A multisite, randomized controlled open-label trial of a novel patient expectations communication tool. J Am Coll Emerg Physicians Open 2024;5(4):e13229.
34. Cherpitel CJ. A brief screening instrument for problem drinking in the emergency room: the RAPS4. Rapid Alcohol Problems Screen. Journal of Studies on Alcohol 2000;61(3):447–449.
35. Cherpitel CJ, Ye Y, Bond J, et al. Cross-national performance of the RAPS4/RAPS4-QF for tolerance and heavy drinking: data from 13 countries. J Stud Alcohol 2005;66(3):428–32.

36. Cherpitel CJ, Bazargan S. Screening for alcohol problems: comparison of the audit, RAPS4 and RAPS4-QF among African American and Hispanic patients in an inner city emergency department. Drug and Alcohol Dependence 2003;71(3):275–280.
37. Cherpitel CJ, Borges G. Screening for drug use disorders in the emergency department: performance of the rapid drug problems screen (RDPS). Drug Alcohol Depend 2004;74(2):171–5.
38. Cherpitel CJ, Ye Y, Moskalewicz J, et al. Screening for alcohol problems in two emergency service samples in Poland: Comparison of the RAPS4, CAGE and AUDIT. Drug and Alcohol Dependence 2005;80(2):201–207.
39. Cherpitel CJ, Ye Y, Bond J, et al. Validity of self-reported drinking before injury compared with a physiological measure: cross-national analysis of emergency-department data from 16 countries. J Stud Alcohol Drugs 2007;68(2):296–302.
40. Chong E, Bao M, Goh EF, et al. SARC-F at the Emergency Department: Diagnostic Performance for Frailty and Predictive Performance for Reattendances and Acute Hospitalizations. J Nutr Health Aging 2021;25(9):1084–1089.
41. Chung H, Lee JH. Clinical validation of screening test for at-risk drinking for young and middle-aged adults in an emergency department. Hong Kong Journal of Emergency Medicine 2020;30(4):203–209.
42. Ciccolo G, Curt A, Camargo CA, Jr., et al. Improving Understanding of Screening Questions for Social Risk and Social Need Among Emergency Department Patients. West J Emerg Med 2020;21(5):1170–1174.
43. Clemens V, Saller MM, Meller R, et al. Clinical Acuity in the Emergency Department and Injury Severity Determine Hospital Admission of Older Patients with Low Energy Falls: Outcomes from a Prospective Feasibility Study. J Clin Med 2023;12(9).
44. Cloutier RL, Fuller BE, Hendrickson RG, et al. Measuring nausea in emergency department patients via the use of exploratory factor analysis. Acad Emerg Med 2010;17(6):e33–9.
45. Cotte F, Mueller T, Gilbert S, et al. Safety of triage self-assessment using a symptom assessment app for walk-in patients in the emergency care setting: observational prospective cross-sectional study. JMIR mHealth and uHealth 2022;10(3):e32340.
46. Cremonte M, Cherpitel CJ. Performance of screening instruments for alcohol use disorders in emergency department patients in Argentina. Subst Use Misuse 2008;43(1):125–38.
47. Curiati PK, Arruda MDS, Carpenter CR, et al. Predicting posthospitalization falls in Brazilian older adults: External validation of the Carpenter instrument. Acad Emerg Med 2024;31(7):688–695.
48. Dasgupta P, Frisch A, Huber J, et al. Predicting falls within 3 months of emergency department discharge among community-dwelling older adults using self-report tools versus a brief functional assessment. Am J Emerg Med 2022;53:245–249.
49. de Almeida Tavares JP, Sa-Couto P, Boltz M, et al. Portuguese older adults presenting at the emergency department: predictive validity of the Identification of Seniors at Risk (ISAR) tool. Journal of gerontological nursing 2017;43(8):18–24.
50. Demir Akça AS, Kafadar D, Kahveci FO, et al. Performance of Identification of Seniors at Risk tool to predict unplanned revisits to the emergency department in 6 months was low in predictive accuracy: A single-center study. Hong Kong Journal of Emergency Medicine 2021;28(2):72–78.
51. Dendukuri N, McCusker J, Belzile E. The identification of seniors at risk screening tool: further evidence of concurrent and predictive validity. Journal of the American Geriatrics Society 2004;52(2):290–296.
52. Desjardins I, Cats-Baril W, Maruti S, et al. Suicide Risk Assessment in Hospitals: An Expert System-Based Triage Tool. J Clin Psychiatry 2016;77(7):e874–82.

53. Devon HA, Rosenfeld A, Steffen AD, et al. Sensitivity, specificity, and sex differences in symptoms reported on the 13-item acute coronary syndrome checklist. J Am Heart Assoc 2014;3(2):e000586.
54. Dionne CE, Le Sage N, Franche RL, et al. Five questions predicted long-term, severe, back-related functional limitations: evidence from three large prospective studies. J Clin Epidemiol 2011;64(1):54–66.
55. Doran KM, Johns E, Zuiderveen S, et al. Development of a homelessness risk screening tool for emergency department patients. Health Serv Res 2022;57(2):285–293.
56. Dresden SM, Platts-Mills TF, Kandasamy D, et al. Patient Versus Physician Perceptions of Frailty: A Comparison of Clinical Frailty Scale Scores of Older Adults in the Emergency Department. Acad Emerg Med 2019;26(9):1089–1092.
57. Ennis N, Anton M, Bravoco O, et al. Prediction of posttraumatic stress and depression one-month post-injury: A comparison of two screening instruments. Health psychology 2021;40(10):702.
58. Ernst AA, Weiss SJ, Cham E, et al. Detecting ongoing intimate partner violence in the emergency department using a simple 4-question screen: the OVAT. Violence Vict 2004;19(3):375–84.
59. Ernst AA, Weiss SJ, Morgan-Edwards S, et al. Derivation and validation of a short emergency department screening tool for perpetrators of intimate partner violence: the PErpetrator RaPid Scale (PERPS). The Journal of emergency medicine 2012;42(2):206–217.
60. Fabacher DA, Raccio-Robak N, McErlean MA, et al. Validation of a brief screening tool to detect depression in elderly ED patients. Am J Emerg Med 2002;20(2):99–102.
61. Faqar-Uz-Zaman SF, Anantharajah L, Baumartz P, et al. The Diagnostic Efficacy of an App-based Diagnostic Health Care Application in the Emergency Room: eRadaR-Trial. A prospective, Double-blinded, Observational Study. Ann Surg 2022;276(5):935–942.
62. Flynn SB, Gordee A, Kuchibhatla M, et al. Moving toward patient-centered care in the emergency department: Patient-reported expectations, definitions of success, and importance of improvement in pain-related outcomes. Acad Emerg Med 2021;28(11):1286–1298.
63. Fox GWC, Rodriguez S, Rivera-Reyes L, et al. PROMIS Physical Function 10-Item Short Form for Older Adults in an Emergency Setting. J Gerontol A Biol Sci Med Sci 2020;75(7):1418–1423.
64. Fraser HSF, Cohan G, Koehler C, et al. Evaluation of Diagnostic and Triage Accuracy and Usability of a Symptom Checker in an Emergency Department: Observational Study. JMIR Mhealth Uhealth 2022;10(9):e38364.
65. Friedman BW, Schechter CB, Mulvey L, et al. Derivation of an abbreviated instrument for use in emergency department low back pain research: the five-item Roland Morris Questionnaire. Acad Emerg Med 2013;20(10):1013–21.
66. Friedmann PD, Saitz R, Gogineni A, et al. Validation of the screening strategy in the NIAAA "Physicians' Guide to Helping Patients with Alcohol Problems". J Stud Alcohol 2001;62(2):234–8.
67. Fulbrook P, Lawrence P, Watt K. Validity of the Paddington Alcohol Test in an Australian Emergency Departmentdagger. Alcohol Alcohol 2015;50(4):407–12.
68. Fulfer JL, Tyler JJ, Choi NJ, et al. Using indirect questions to detect intimate partner violence: the SAFE-T questionnaire. Journal of interpersonal violence 2007;22(2):238–249.
69. Gamst-Jensen H, Trondarson T, Kallemose T, et al. How well do nurses know their patients? Agreement between patients' degree-of-worry and nurses' estimation of patients' degree-of-worry-An observational study. Scand J Caring Sci 2023;37(3):654–661.
70. Gentile S, Lacroix O, Durand A, et al. Malnutrition: a highly predictive risk factor of short-term mortality in elderly presenting to the emergency department. The Journal of nutrition, health and aging 2013;17(4):290–294.

71. Gibbons RD, Alegria M, Markle S, et al. Development of a computerized adaptive substance use disorder scale for screening and measurement: the CAT-SUD. Addiction 2020;115(7):1382–1394.
72. Graf CE, Giannelli SV, Chevalley T, et al. Identification of older patients at risk of unplanned readmission after discharge from the emergency department. Swiss medical weekly 2012;142(0102):w13327–w13327.
73. Greenberg M, Jacoby J, Barraco RD, et al. Analysis of Falls Efficacy Scale and Vulnerable Elders Survey as Predictors of Falls. Cureus 2021;13(4):e14471.
74. Greene SJ, Mentz RJ, Limkakeng AT, et al. Comparison of Dyspnea Measurement Instruments in Acute Heart Failure: The DYSPNEA-AHF Pilot Study. J Card Fail 2021;27(5):607–609.
75. Guo JW, Wallace AS, Luther BL, et al. Psychometric Evaluation of the Screener for Intensifying Community Referrals for Health. Eval Health Prof 2022;45(3):270–276.
76. Halpern LR, Susarla SM, Dodson TB. Injury location and screening questionnaires as markers for intimate partner violence. J Oral Maxillofac Surg 2005;63(9):1255–61.
77. Haughey MT, Calderon Y, Torres S, et al. Identification of depression in an inner-city population using a simple screen. Acad Emerg Med 2005;12(12):1221–6.
78. Heeren P, Devriendt E, Wellens NIH, et al. Old and New Geriatric Screening Tools in a Belgian Emergency Department: A Diagnostic Accuracy Study. J Am Geriatr Soc 2020;68(7):1454–1461.
79. Holdgate A, Asha S, Craig J, et al. Comparison of a verbal numeric rating scale with the visual analogue scale for the measurement of acute pain. Emerg Med (Fremantle) 2003;15(5-6):441–6.
80. Houry D, Feldhaus K, Peery B, et al. A positive domestic violence screen predicts future domestic violence. Journal of Interpersonal Violence 2004;19(9):955–966.
81. Houry D, Kemball RS, Click LA, et al. Development of a brief mental health screen for intimate partner violence victims in the emergency department. Academic Emergency Medicine 2007;14(3):202–209.
82. Hsu HP, Cheng MT, Lu TC, et al. Pain Assessment in the Emergency Department: A Prospective Videotaped Study. West J Emerg Med 2022;23(5):716–723.
83. Hudon C, Bisson M, Dubois MF, et al. CONECT-6: a case-finding tool to identify patients with complex health needs. BMC Health Serv Res 2021;21(1):157.
84. Hustey FM. The use of a brief depression screen in older emergency department patients. Acad Emerg Med 2005;12(9):905–8.
85. Hyphantis T, Kotsis K, Kroenke K, et al. Lower PHQ-9 cutpoint accurately diagnosed depression in people with long-term conditions attending the Accident and Emergency Department. J Affect Disord 2015;176:155–63.
86. Hyphantis T, Kroenke K, Papatheodorou E, et al. Validity of the Greek version of the PHQ 15-item Somatic Symptom Severity Scale in patients with chronic medical conditions and correlations with emergency department use and illness perceptions. Compr Psychiatry 2014;55(8):1950–9.
87. Jaramillo S, Suffoletto B, Callaway C, et al. Early Screening for Posttraumatic Stress Disorder and Depression Among Injured Emergency Department Patients: A Feasibility Study. Acad Emerg Med 2019;26(11):1232–1244.
88. Jarman H, Crouch R, Baxter M, et al. Feasibility and accuracy of ED frailty identification in older trauma patients: a prospective multi-centre study. Scand J Trauma Resusc Emerg Med 2021;29(1):54.
89. Jensen AN, Kallemose T, Frostholm L, et al. Correlation between illness perceptions and self-reported degree-of-worry in somatic ill patients in emergency care: a Danish cross-sectional study. BMJ open 2024;14(10):e089595.
90. Juniper EF, Svensson K, Mörk AC, et al. Measuring health-related quality of life in adults during an acute asthma exacerbation. Chest 2004;125(1):93–97.

91. Kaltiso SO, Greenbaum VJ, Moran TP, et al. Feasibility of a screening tool for sex trafficking in an adult emergency department. Acad Emerg Med 2021;28(12):1399–1408.
92. Kendrick KR, Baxi SC, Smith RM. Usefulness of the modified 0-10 Borg scale in assessing the degree of dyspnea in patients with COPD and asthma. Journal of Emergency Nursing 2000;26(3):0216–0222.
93. Kene M, Miller Rosales C, Wood S, et al. Feasibility of expanded emergency department screening for behavioral health problems. Am J Manag Care 2018;24(12):585–591.
94. Kiechle ES, Hnat AT, Norman KE, et al. Comparison of brief health literacy screens in the emergency department. J Health Commun 2015;20(5):539–45.
95. Knightly R, Tadros G, Sharma J, et al. Alcohol screening for older adults in an acute general hospital: FAST v. MAST-G assessments. BJPsych Bull 2016;40(2):72–6.
96. Ku NW, Cheng MT, Liew CQ, et al. Prospective study of pain and patient outcomes in the emergency department: a tale of two pain assessment methods. Scand J Trauma Resusc Emerg Med 2023;31(1):56.
97. Kumodzi T, Kassam-Adams N, Vargas L, et al. Comparison of two screeners predicting the future development of depression and posttraumatic stress disorder in Black men after serious injury. Injury 2022;53(5):1678–1683.
98. Lee J, Min S, Ahn JS, et al. Identifying alcohol problems among suicide attempters visiting the emergency department. BMC Psychiatry 2019;19(1):350.
99. Lee KW, Choi YH, Lee JH. Cut-off points for screening at-risk drinking by AUDIT-C Korean version at emergency department. Turk J Emerg Med 2018;18(2):57–61.
100. Liu H, Shang N, Chhetri J, et al. A frailty screening questionnaire (FSQ) to rapidly predict negative health outcomes of older adults in emergency care settings. The journal of nutrition, health & aging 2020;24(6):627–633.
101. Loddo S, Costaggiu D, Palimodde A, et al. Emergency department: risk stratification in the elderly. Journal of Gerontology and Geriatrics 2021;69(3):164–170.
102. Lotfipour S, Howard J, Roumani S, et al. Increased detection of alcohol consumption and at-risk drinking with computerized alcohol screening. J Emerg Med 2013;44(4):861–6.
103. Masoudi Alavi N, Aboutalebi MS, Sadat Z. Verbal and Non-verbal Rating Scales in the Determination of Pain Severity in Trauma Patients in the Emergency Department. Trauma Monthly 2017;22(1).
104. McCabe MP, Staiger PK, Thomas AC, et al. Screening for comorbid substance use disorders among people with a mental health diagnosis who present to emergency departments. Australasian Emergency Nursing Journal 2011;14(3):163–171.
105. McCusker J, Cardin S, Bellavance Fo, et al. Return to the emergency department among elders: patterns and predictors. Academic Emergency Medicine 2000;7(3):249–259.
106. McCusker J, Bellavance F, Cardin S, et al. Prediction of hospital utilization among elderly patients during the 6 months after an emergency department visit. Ann Emerg Med 2000;36(5):438–45.
107. McLean SA, Kirsch NL, Tan-Schriner CU, et al. Health status, not head injury, predicts concussion symptoms after minor injury. Am J Emerg Med 2009;27(2):182–90.
108. Medeiros FC, Costa LOP, Oliveira IS, et al. The use of STarT BACK Screening Tool in emergency departments for patients with acute low back pain: a prospective inception cohort study. Eur Spine J 2018;27(11):2823–2830.
109. Meek R, Egerton-Warburton D, Mee MJ, et al. Measurement and monitoring of nausea severity in emergency department patients: a comparison of scales and exploration of treatment efficacy outcome measures. Acad Emerg Med 2015;22(6):685–93.
110. Meneses-Gaya C, Crippa JA, Zuardi AW, et al. The fast alcohol screening test (FAST) is as good as the AUDIT to screen alcohol use disorders. Subst Use Misuse 2010;45(10):1542–57.

111. Meneses-Gaya C, Zuardi AW, Loureiro SR, et al. Is the full version of the AUDIT really necessary? Study of the validity and internal construct of its abbreviated versions. Alcohol Clin Exp Res 2010;34(8):1417–24.
112. Mills TJ, Avegno JL, Haydel MJ. Male victims of partner violence: prevalence and accuracy of screening tools. J Emerg Med 2006;31(4):447–52.
113. Moons P, De Ridder K, Geyskens K, et al. Screening for risk of readmission of patients aged 65 years and above after discharge from the emergency department: predictive value of four instruments. European Journal of Emergency Medicine 2007;14(6):315–323.
114. Mumma BE, Scofield ME, Mendoza LP, et al. Screening for Victims of Sex Trafficking in the Emergency Department: A Pilot Program. West J Emerg Med 2017;18(4):616–620.
115. Neumann T, Neuner B, Gentilello LM, et al. Gender differences in the performance of a computerized version of the alcohol use disorders identification test in subcritically injured patients who are admitted to the emergency department. Alcohol Clin Exp Res 2004;28(11):1693–701.
116. Ng M, Liu Z, Tan JSL, et al. Comparison of self-reported EDACS versus physician-reported EDACS for the triage of chest pain patients in the emergency department. J Am Coll Emerg Physicians Open 2021;2(2):e12393.
117. Nock MK, Millner AJ, Ross EL, et al. Prediction of Suicide Attempts Using Clinician Assessment, Patient Self-report, and Electronic Health Records. JAMA Netw Open 2022;5(1):e2144373.
118. O'Connor L, Larkin C, Ibrahim AF, et al. Development and pilot study of simple suicide risk rulers for use in the emergency department. Gen Hosp Psychiatry 2020;63:97–102.
119. O'Reilly LM, Dalal AI, Maag S, et al. Computer adaptive testing to assess impairing behavioral health problems in emergency department patients with somatic complaints. J Am Coll Emerg Physicians Open 2022;3(5):e12804.
120. Pacella-LaBarbara M, Larsen SE, Jaramillo S, et al. Event centrality following treatment for physical injury in the emergency department: Associations with posttraumatic outcomes. Gen Hosp Psychiatry 2020;67:77–82.
121. Paranjape A, Liebschutz J. STaT: a three-question screen for intimate partner violence. Journal of Women's Health 2003;12(3):233–239.
122. Perez Elias MJ, Gomez-Ayerbe C, Perez Elias P, et al. Development and Validation of an HIV Risk Exposure and Indicator Conditions Questionnaire to Support Targeted HIV Screening. Medicine (Baltimore) 2016;95(5):e2612.
123. Platts-Mills TF, Nebolisa BC, Flannigan SA, et al. Post-Traumatic Stress Disorder among Older Adults Experiencing Motor Vehicle Collision: A Multicenter Prospective Cohort Study. Am J Geriatr Psychiatry 2017;25(9):953–963.
124. Poudel M, Bhandari R, Malla G, et al. Rose Angina Questionnaire: Validation in emergency department to detect myocardial infarction in a tertiary hospital of eastern Nepal. Hong Kong Journal of Emergency Medicine 2019;27(5):257–261.
125. Punches BE, Freiermuth CE, Sprague JE, et al. Screening for problematic opioid use in the emergency department: Comparison of two screening measures. J Am Coll Emerg Physicians Open 2024;5(1):e13106.
126. Randall JR, Rowe BH, Colman I. Emergency department assessment of self-harm risk using psychometric questionnaires. The Canadian Journal of Psychiatry 2012;57(1):21–28.
127. Reyes-Gibby CC, Anderson KO, Todd KH. Risk for Opioid Misuse Among Emergency Department Cancer Patients. Acad Emerg Med 2016;23(2):151–8.
128. Richards CT, Gisondi MA, Chang C-H, et al. Palliative care symptom assessment for patients with cancer in the emergency department: validation of the screen for palliative and end-of-life care needs in the emergency department instrument. Journal of palliative medicine 2011;14(6):757–764.

129. Richards DK, Morera OF, Cabriales JA, et al. Factor, Concurrent and Predictive Validity of the Readiness to Change Questionnaire [Treatment Version] Among Non-Treatment-Seeking Individuals. Alcohol Alcohol 2020;55(4):409–415.
130. Rizka A, Ambarwati RA, Mansjoer A, et al. Performance of Identification of the Seniors At Risk (ISAR) tool and Triage Risk-Screening Tool (TRST) for frail elderly emergency room patients. Infection 2021;70:S42–S45.
131. Rizka A, Harimurti K, Pitoyo CW, et al. Comparison between the Identification of Seniors at Risk and Triage Risk Screening Tool in predicting mortality of older adults visiting the emergency department: Results from Indonesia. Geriatr Gerontol Int 2020;20(1):47–51.
132. Rodriguez-Martos A, Santamarina E. Does the short form of the Alcohol Use Disorders Identification Test (AUDIT-C) work at a trauma emergency department? Subst Use Misuse 2007;42(6):923–32.
133. Salvi F, Morichi V, Grilli A, et al. Screening for frailty in elderly emergency department patients by using the Identification of Seniors At Risk (ISAR). The journal of nutrition, health & aging 2012;16(4):313–318.
134. Salvi F, Morichi V, Grilli A, et al. Predictive validity of the Identification of Seniors At Risk (ISAR) screening tool in elderly patients presenting to two Italian Emergency Departments. Aging clinical and experimental research 2009;21(1):69–75.
135. Salvi F, Morichi V, Lorenzetti B, et al. Risk stratification of older patients in the emergency department: comparison between the Identification of Seniors at Risk and Triage Risk Screening Tool. Rejuvenation research 2012;15(3):288–294.
136. Saracino A, Weiland T, Dent A, et al. Validation of a verbal dyspnoea rating scale in the emergency department. Emerg Med Australas 2008;20(6):475–81.
137. Saracino A, Weiland TJ, Jolly B, et al. Verbal dyspnoea score predicts emergency department departure status in patients with shortness of breath. Emerg Med Australas 2010;22(1):21–9.
138. Sattler AF, Bentley JP, Young J. Validation of a brief screening instrument for adult psychopathology. Professional Psychology: Research and Practice 2019;50(5):315–322.
139. Schriger DL, Gibbons PS, Langone CA, et al. Enabling the diagnosis of occult psychiatric illness in the emergency department: a randomized, controlled trial of the computerized, self-administered PRIME-MD diagnostic system. Annals of emergency medicine 2001;37(2):132–140.
140. Schuijt H, Oud F, Bruns E, et al. Does the Dutch Safety Management Program predict adverse outcomes for older patients in the emergency department? Neth J Med 2020;78(5):244–50.
141. Seale JP, Johnson JA, Cline N, et al. Drug screening and changing marijuana policy: Validation of new single question drug screening tools. Drug Alcohol Depend 2018;193:104–109.
142. Sen P, Barnicot K, Podder P, et al. Exploring the prevalence of personality disorder and the feasibility of using the SAPAS as a screening tool for personality disorder in an emergency department in India. Medicine, Science and the Law 2022;62(1):8–16.
143. Shah MN, Richardson TM, Jones CM, et al. Depression and cognitive impairment in older adult emergency department patients: changes over 2 weeks. J Am Geriatr Soc 2011;59(2):321–6.
144. Shang N, Liu H, Wang N, et al. Comparison of three frailty screening instruments for prediction of adverse outcomes among older adults in the emergency department. Geriatr Gerontol Int 2022;22(10):851–856.
145. Shenvi CL, Weaver MA, Biese KJ, et al. Identification and characterization of older emergency department patients with high-risk alcohol use. J Am Coll Emerg Physicians Open 2020;1(5):804–811.
146. Shrestha R, Shrestha AP, Shrestha A, et al. Unrecognized geriatric depression in the emergency Department of a Teaching Hospital in Nepal: prevalence, contributing factors, and metric properties of 5 item geriatric depression scale in this population. BMC Psychiatry 2020;20(1):533.

147. Simpson SA, Goans C, Loh R, et al. Suicidal ideation is insensitive to suicide risk after emergency department discharge: Performance characteristics of the Columbia-Suicide Severity Rating Scale Screener. Acad Emerg Med 2021;28(6):621–629.
148. Singh SM, Bhalla A, Giri OP, et al. Development of Screening Questionnaire for Detection of Alcohol Dependence. J Clin Diagn Res 2015;9(9):VC07–VC10.
149. Singler K, Heppner HJ, Skutetzky A, et al. Predictive validity of the identification of seniors at risk screening tool in a German emergency department setting. Gerontology 2014;60(5):413–9.
150. Snijders BMG, Emmelot-Vonk MH, Souwer ETD, et al. Prognostic value of screening instrument based on the Dutch national VMS guidelines for older patients in the emergency department. Eur Geriatr Med 2021;12(1):143–150.
151. Solie CJ, Swanson MB, Harland K, et al. Two-Item Fall Screening Tool Identifies Older Adults at Increased Risk of Falling after Emergency Department Visit. West J Emerg Med 2020;21(5):1275–1282.
152. Stewart SH, Doscher A, Miles S, et al. Identification and risk-stratification of problem alcohol drinkers with minor trauma in the emergency department. West J Emerg Med 2010;11(2):133–7.
153. Suffoletto B, Miller T, Shah R, et al. Predicting older adults who return to the hospital or die within 30 days of emergency department care using the ISAR tool: subjective versus objective risk factors. Emergency Medicine Journal 2016;33(1):4–9.
154. Sung SC, Ma J, Earnest A, et al. Screening for panic-related anxiety in emergency department patients with cardiopulmonary complaints: A comparison of two self-report instruments. Psychiatry Res 2018;263:7–14.
155. Svavarsdottir EK. Detecting intimate partner abuse within clinical settings: self-report or an interview. Scand J Caring Sci 2010;24(2):224–32.
156. Tan CIC, Liaw JSC, Jiang B, et al. Predicting outcomes of acute low back pain patients in emergency department: A prospective observational cohort study. Medicine (Baltimore) 2018;97(26):e11247.
157. Tavares JPA, Sa-Couto P, Boltz M, et al. Identification of Seniors at Risk (ISAR) in the emergency room: A prospective study. Int Emerg Nurs 2017;35:19–24.
158. Thompson RG, Jr., Mullinax S, De Monte R, et al. Effectiveness of a Self-Administered Computerized Mental Health Screening Tool in the Emergency Department. Psychiatr Serv 2023;74(11):1180–1184.
159. Tiedemann A, Sherrington C, Orr T, et al. Identifying older people at high risk of future falls: development and validation of a screening tool for use in emergency departments. Emerg Med J 2013;30(11):918–22.
160. Trautman DE, McCarthy ML, Miller N, et al. Intimate partner violence and emergency department screening: computerized screening versus usual care. Ann Emerg Med 2007;49(4):526–34.
161. Tuli R, Romero SA, Figueroa C, et al. Investigating a Substance Abuse Screening in a Trauma Setting. Am Surg 2021;87(10):1606–1611.
162. Ungar OJ, Cavel O, Oron Y, et al. A subjective rating scale for initial assessment of sudden unilateral sensorineural hearing loss. Audiology and Neurotology 2017;22(3):154–159.
163. Vaittinada Ayar P, Taille C, Vaittinada Ayar P, et al. Assessment of Predictor Factors Associated with Multiple Emergency Department Attendance with Asthma Attack: A Qualitative and Multicentric Prospective Observational Study. J Asthma Allergy 2022;15:303–313.
164. van Dam CS, Moss N, Schaper SA, et al. Screening instruments for identification of vulnerable older adults at the emergency department: A critical appraisal. Acute Med 2018;17(3):124–9.
165. van Dam CS, Trappenburg MC, Ter Wee MM, et al. The accuracy of four frequently used frailty instruments for the prediction of adverse health outcomes among older adults at two Dutch

emergency departments: findings of the AmsterGEM study. Annals of Emergency Medicine 2021;78(4):538–548.
166. Van Der Westhuizen C, Wyatt G, Williams JK, et al. Validation of the Alcohol, Smoking and Substance Involvement Screening Test in a low-and middle-income country cross-sectional emergency centre study. Drug and alcohol review 2016;35(6):702–709.
167. van der Westhuizen C, Wyatt G, Williams JK, et al. Validation of the Self Reporting Questionnaire 20-Item (SRQ-20) for Use in a Low- and Middle-Income Country Emergency Centre Setting. Int J Ment Health Addict 2016;14(1):37–48.
168. Weiner SG, Horton LC, Green TC, et al. A comparison of an opioid abuse screening tool and prescription drug monitoring data in the emergency department. Drug Alcohol Depend 2016;159:152–7.
169. Weiss SJ, Ernst AA, Cham E, et al. Development of a screen for ongoing intimate partner violence. Violence and victims 2003;18(2):131.
170. White A, Afsari M, Balakrishnan H, et al. Implementation of universal screening for substance use in pregnancy in a public healthcare system. AJOG Glob Rep 2024;4(3):100384.
171. Williams R, Vinson DC. Validation of a single screening question for problem drinking. J Fam Pract 2001;50(4):307–12.
