# Supplementary material for: Patient-Reported Outcome Measures in the Emergency Department: A Scoping Review
Source: J Am Coll Emerg Physicians Open. 2026 Aug 17;7(5):100486. doi: 10.1016/j.acepjo.2026.100486 (PMC13520099; doi:10.1016/j.acepjo.2026.100486)
Supplement: Supplementary file 4 [file mmc4.pdf]

Table S4: Description of identified PROM by category, topic, subtopic, PROM name and patient group

Topic	Subtopic	PROMs	Applied to ED patients (otherwise specified)
**Generic category**			
**Expectation**		Expectation questionnaire [a]	
**Health and symptoms**	**General health**	EuroQol 5 Dimensions (**EQ-5D**)	Older ED patients with a low energy fall
		Patient Reported Outcome Measurement Information System (**PROMIS**) Physical Function (**PF**) 10 item short form	≥ 65 years
		Short-Form Health Survey (**SF-8**)	Female ED patients
		Short-Form 36 health survey (**SF-36**)	(i) Pt with minor injury, (ii) Older ED patients with a low energy fall
	**Impact of illness**	Brief-illness Perception Questionnaire (B-IPQ)	ED patients with somatic illness
		Patient-centered Outcomes Questionnaire (PCOQ)	ED patients with musculoskeletal pain
	**Symp-toms**	Ada-app (symptom assessment/triage)[b]	(i) ED patients (ii) ED patients with abdominal pain
		Patient Health Questionnaire-15 (PHQ-15)	ED patients with at least one of three long-term physical conditions — diabetes mellitus (DM), rheumatological disorder (RD) and chronic obstructive pulmonary disease (COPD)
		Sickness Impact Profile Alertness Behavior subscale (SIPab)	ED patients with minor injury
**Health Literacy**		Health literacy questionnaire by European Consortium (HL-SF12)	ED patients and patients visiting medical Outdoor Patients Department
		Medical Term Recognition Test (METER)	
		Single-item literacy screener (SILS)	
		Validated Screening Questions [a]	
**Mental health**	**Anxiety**	General Anxiety Disorder questionnaire 2 items (GAD-2)	
		Generalized Anxiety Disorder Scale 7 items (GAD-7)	≥ 60 years
		Patient health questionnaire – panic disorder module (PHQ-PD)	ED patients with primary complaint of palpitations, chest pain, dizziness, or shortness of breath
		Psychiatric diagnostic screening questionnaire – panic disorder module (PDSQ-PD)	ED patients with primary complaint of palpitations, chest pain, dizziness, or shortness of breath

Table S4. (Continued)

Topic	Subtopic	PROMs	Applied to ED patients (otherwise specified)
		**Generic category**	
**Mental health**	**Depression**	Beck Depression Inventory II (BDI-II)	Female ED patients between 18 and 55 years
		Center for Epidemiologic Studies–Depression (CES-D) scale	ED cancer patients with pain
		Computerized Adaptive Test-Depression Inventory (CAT-DI)[b]	(i) ED patients with nonpsychiatric chief complaints (ii) ED patients
		Computerized Adaptive Diagnostic Test for Major Depressive Disorder (CAD-MDD)[b]	ED patients with nonpsychiatric chief complaints
		Patient Health Questionnaire (PHQ-2)	(i) ED patients (ii) ≥ 60 years
		Patient Health Questionnaire (PHQ-9)	(i) Patients with a chief complaint of self-injurious thoughts and behaviour (SITB) (ii) Patients with at least one of three long-term physical conditions - DM, RD and COPD (iii) ≥ 60 years
		"Short two questions scale" or "Brief two question depression screen” [a]	(i) ED patients (ii) ≥ 70 years
	**General**	Barrett Impulsiveness Scale (BIS)	
		Beck Hopelessness Scale (BHS)	
		Brief Screening Instrument for Adult Psychopathology[a,b]	
		Brief Symptom Inventory (BSI)	
		Catastrophizing subscale of the Coping Strategies Questionnaire (CSQ)	ED patients with cancer and pain
		Computer adaptive test - mental health (CAT-MH)[b]	
		Computerized Assessment and Referral System (CARS)[b]	ED patients presented to the ED for reasons unrelated to mental health conditions at the current visit and without a psychiatric diagnose
		Degree-of-worry (DOW)	ED patients with somatic illness
		General Health Questionnaire 12 item (GHQ-12)	ED patients with an asthma exacerbation

Table S4. (Continued)

Topic	Subtopic	PROMs	Applied to ED patients (otherwise specified)
**Generic category**			
**Mental health**	**General**	Patient Reported Outcome Measurement Information System (PROMIS) (Sleep disturbance, anxiety and depression)	ED patients with cancer
		Primary Care Evaluation of Mental Disorders (PRIME-MD)[c]	(i) ≥ 65 years after a motor vehicle collision (ii) ED patients with a complaint likely to be associated with an occult psychiatric illness
		Self Reporting Questionnaire (SRQ)	Adult ED patients presented with treatment of an assault injury or an unintentional injury
		Standardized Assessment of Personality – Abbreviated Scale (SAPAS)	
	**PTSD**	6-item PTSD Checklist – short version	ED patients with injury and met criterion A of the PTSD diagnosis
		Life-threatening experience[a]	≥ 60 years after a motor vehicle collision
		Peritraumatic Dissociative Experiences Questionnaire (10-items)-Self-Report Version (PDQE-10-SRV)	(i) ED patients with injury and met criterion A of the PTSD diagnosis (ii) ED patients - victims of a violent assault
		Peritraumatic distress inventory (PDI)	(i) ≥ 60 years after a motor vehicle collision (ii) Patients with traumatic injury
		Posttraumatic Stress Diagnostic Scale (PDS)	All female patients between the ages of 18 and 55 years
		Pre-injury psychological treatment[a]	ED patients with injury and met criterion A of the PTSD diagnosis
	**PTSD and depression**	Injured trauma survivor screen (ITSS)	Patients with traumatic injury
		Penn Richmond Screener	ED patients, black male and hospitalized for an acute traumatic injury
		Posttraumatic Adjustment Scale (PAS)	(i) Patients with injury (ii) ED patients, black male and hospitalized for an acute traumatic injury
	**Suicide**	0 - to 10 -point rulers [a]	
		Becks scale for suicide ideation (BSS)[c]	(i) ED patients (ii) Patients with a chief complaint of self-injurious thoughts and behaviour (SITB) (iii) Female patients between 18 and 55 years
		Columbia Suicide Severity Scale (C-SSRS)	(i) ED patients (ii) Patients with a chief complaint of self-injurious thoughts and behaviour (SITB)

Table S4. (Continued)

Topic	Subtopic	PROMs	Applied to ED patients (otherwise specified)
**Generic category**			
**Mental health**	**Suicide**	Convergent Functional Information for Suicidality (CFI-S)	
		Patient Safety Screener (PSS)	
		Patient Self-Report Survey[a,b]	
		Psychiatric Emergency Research Collaboration (PERC screener)	
		Suicide Risk Questionnaire	
**Older people**	**Depression**	ED-DSI	≥ 65 years
		Geriatric Depression Scale (GDS-5)	≥ 60 years
		Geriatric Depression Scale (GDS-15)	≥ 65 years
		Mini Geriatric Depression Scale (Mini-GDS)	≥ 75 years
	**Fall**	Carpenter	≥ 65 years
		Falls Efficacy Scale (FES)	≥ 65 years. In case of a mechanical fall, risk was defined by Centers for Disease Control and Prevention criteria
		Single Item Screen for Mobility (SIS-M)	≥ 60 years, community-dwelling
		Stay Independent Questionnaire (SIQ-12)	≥ 60 years, community-dwelling
		Two-item screening tool (Tiedemann's rule)[a]	(i) ≥ 65 years (ii) ≥ 70 years who presented to EDs due to a fall or with a history of two or more falls in the past year
		Vulnerable Elder Survey (VES-13)	≥ 65 years. In case of a mechanical fall, risk was defined by Centers for Disease Control and Prevention criteria
	**Frailty**	Clinical Frailty Scale	Community-dwelling patients ≥ 65 years
		FRAIL scale (FS)	(i) ≥ 60 (ii) ≥ 65 years
		Frailty Screening Questionnaire (FSQ)	≥ 65 years

Table S4. (Continued)

Topic	Subtopic	PROMs	Applied to ED patients (otherwise specified)
**Generic category**			
**Older people**	**General**	A 27-item self-report questionnaire[a]	≥ 65 years
		Brief Risk Identification for Geriatric Health Tool (BRIGHT)	≥ 75 years (65 years for Maori and Pasifika elders)
		Identification of Seniors At Risk (ISAR)	(i) ≥ 60, (ii) ≥ 65, (iii) ≥ 70, (iv) ≥ 75 years or above<br>(ii) Community-dwelling or institutionalized ED patients ≥ 65 years of age or older
		Identification of Senior At Risk Hospitalized Patients (ISAR-HP)	≥ 65 years
		Older Americans Resources and Services scale (OARS)	≥ 65 years
		"Program of Research on the Integration of Services for the Maintenance of Autonomy" (PRISMA-7)	(i) ≥ 65 years with activation of trauma team (ii) ≥ 75 years
		Rowland	≥ 65 years
		SARC-F	≥ 85 years
		The Dutch Safety Management System [Veiligheidsmanagementsysteem] (VMS) screener	≥ 70 years
		The shortened [Veiligheidsmanagementsysteem] VMS-based screening tool	≥ 70 years
	**Nutrition**	Hunger Vital Sign (HVS)	≥60 years
		Malnutrition Screening Tool (MST)	≥60 years
**Partner violence**		Abuse Assessment Screen (ASS)	
		Brief mental health screen	Female ED intimate partner violence patients
		Extended - Hurt, Insult, Threaten, Scream (E-HITS)	Female ED patients aged 18 to 60

Table S4. (Continued)

Topic	Subtopic	PROMs	Applied to ED patients (otherwise specified)
**Generic category**			
**Partner violence**		HARK (Humiliation, Afraid, Rape, Kick)	
		HITS (Hurt, Insulted, Threatened with harm, Screamed at them)	(i) ED patients (ii) Male ED patients
		Ongoing Abuse Screen (OAS)	
		Ongoing violence assessment tool (OVAT)	(i) ED patients (with a partner) (ii) ED patients
		Partner Violence Screen (PVS)	(i) Male ED patients (ii) female ED patients
		PErpetration RaPid Scale (PERPS)	
		SAFE-T	Victims of (intimate) partner violence (female) and 80 lay women attending an annual women's health conference, ED patients (female)
		Short-Woman Abuse Screening Tool (short-WAST)	(i) Male ED patients (ii) female ED patients
		Slapped, Things, Threaten (STaT)	Female ED patients between 18 and 64 years
		Universal Violence Prevention Screening Protocol (UVPSP)	Female ED patients
		Women abuse screening tool (WAST)	(i) Women in ED, (ii) pregnant women seeking prenatal care in their first trimester.
**Sex trafficking**		Human trafficking screening tool [a]	ED patients likely to have experienced human trafficking
		Screening survey [a]	Female ED patients
**Social risk**		A three-item tool for a homelessness risk screening [a]	
		COmplex NEeds Case-finding Tool – 6 (CONECT-6)	Patients presented at least one ambulatory care sensitive conditions (ACSC) and had three or more visits to the ED within 12 months
		Health related social needs (HRSN) (modified)	≥60 years

Table S4. (Continued)

Topic	Subtopic	PROMs	Applied to ED patients (otherwise specified)
		**Generic category**	
**Social risk**		Poverty-related Quality of Life (PQOL-17)	
		Screener for Intensifying Community Referrals for Health (SINCERE)	
		Self-perceived risk of future homelessness[a]	
		Social risk and social need survey tool	
**Substance use**	**Alcohol**	Alcohol Dependence questionnaire[a]	ED patients that had consumed alcohol in some form in the previous year
		Alcohol Use Disorder Identification Test (AUDIT)	(i) ED patients (ii) Patients with hispanic background and reported being current drinkers (iii) ED patients from 19 to 65 years of age (iv) ED patients who were considered current drinkers (v) Injured ED patients (vi) ED trauma patients (vii) Patients visiting a psychosocial care centre for alcohol and drug users (viii) ≥65 years (ix) ED patients with a blood alcohol level (BAL) >0.8 g/L, at the time of ED admission
		Alcohol Use Disorders Identification Test – Consumption- 3 (AUDIT-3)	(i) ED patients (ii) Patients visiting a psychosocial care centre for alcohol and drug users
		Alcohol Use Disorders Identification Test – Consumption – 4 (AUDIT-4)	(i) ED patients (ii) Patients visiting a psychosocial care centre for alcohol and drug users
		Alcohol Use Disorders Identification Test - Consumption (AUDIT-C)	(i) ED patients (ii) Patients following a suicide attempt (iii) Patients visiting a psychosocial care centre for alcohol and drug users (iv) Patients with traffic casualties
		Alcohol Use Disorders Identification Test – Consumption - Quantity-Frequency (AUDIT-QF)	(i)ED patients (ii) Patients visiting a psychosocial care centre for alcohol and drug users
		Alcohol Use Disorders Identification Test - primary care (AUDIT-PC)	(i) ED patients (ii) Patients visiting a psychosocial care centre for alcohol and drug users
		Australian modified version of Paddington Alcohol Test (PAT) (AusPAT)	

Table S4. (Continued)

Topic	Subtopic	PROMs	Applied to ED patients (otherwise specified)
		**Generic category**	
**Substance use**	**Alcohol**	Brief Michigan Alcohol Screening test (BMAST)	(i) ED patients (Hispanic background), and reported being current drinkers (ii) ED patients who were considered current drinkers
		CAGE (Cut down, Annoyed, Guilt, Eye-opener)	(i) ED patients (ii) ED patients (Hispanic background), and reported being current drinkers (iii) Injured ED patients (iv) ED patients who were considered current drinkers (v) Patients aged 65 or older (vi) Female ED patients (vii) ED patients with a blood alcohol level (BAL) >0.8 g/L, at the time of ED admission
		Computerized Alcohol Screening and brief Intervention CASI)[b]	
		Diagnostic interview schedule (DIS)	
		Fast Alcohol Consumption Evaluation (FACE)	
		Fast alcohol screening test (FAST)	(i) ED patients (ii) ≥65 years (iii) Patients visiting a psychosocial care centre for alcohol and drug users"
		Five-shot	(i) ED patients (ii) Patients visiting a psychosocial care centre for alcohol and drug users"
		National Institutes of Health (NIH) - recommended heavy drinking day item[a]	Minor trauma patients age ≥ 18
		Rapid Alcohol Problems Screen (RAPS)	
		Rapid Alcohol Problems Screen (RAPS4)	(i) ED patients (ii) ED patients (Hispanic background) and reported being current drinkers (iii) ED patients who were considered current drinkers (iv) ED patients (injured) (v) Minor trauma patients age ≥ 18.
		Rapid Alcohol Problems Screen – Quantity Frequency (RAPS4-QF)	(i) ED patients (ii) ED patients (Hispanic background), and reported being current drinkers (iii) ED patients who were considered current drinkers<br>ED patients with a blood alcohol level (BAL) >0.8 g/L, at the time of ED admission (iv) ED patients (injured)
		Readiness to Change Questionnaire [Treatment Version] (RCQ[TV])	Injured patients with positive blood alcohol concentration or self-reported drinking before injury
		Screening Test for At-risk Drinking (STAD)	ED patients aged 19–65 years

Table S4. (Continued)

Topic	Subtopic	PROMs	Applied to ED patients (otherwise specified)
**Generic category**			
**Substance use**	**Alcohol**	Self-reported drinking[a]	ED patients with injury
		Single screening question for problem drinking[a]	ED patients with injury
		TRAUMA (Skinner's five-item trauma score)	ED patients (Hispanic background) and reported being current drinkers.
		TWEAK (Tolerance, Worried, Eye, Amnesia, K/Cut down)	(i) ED patients (Hispanic background), and reported being current drinkers (ii) ED patients who were considered current drinkers"
	**Drugs**	Brief screen tool for substance use detection[a]	
		Current Opioid Misuse Measure (COMM)	Patients reporting chronic pain persisting for more than 6 months, or presenting with a refill request for a prescription for opioid medication
		Drug Abuse Screening Test (DAST-10)	
		DSM-5 Criteria for Diagnosis of Opioid Use Disorder	
		Opioid Risk Tool (ORT)	Patients reporting chronic pain persisting for more than 6 months, or presenting with a refill request for a prescription for opioid medication
		Rapid drug problems screen (RDPS)	ED patients with injury
		Screener and Opioid Assessment for Patients with Pain-Revised (SOAPP-R)	(i) ED patients with cancer and pain (ii) Patients reporting chronic pain persisting for more than 6 months, or presenting with a refill request for a prescription opioid medication (iii) Patients aged 18 and older who presented to the ED with a painful condition
		Single Drug Screening Question (SDSQ)	
	**General**	Alcohol, Smoking and Substance Involvement Screening Test (ASSIST)	(i) ED patients (ii) ED patients with mental illness (iii) ED trauma patients (iv) Adult ED patients presenting with treatment of an assault injury or an unintentional injury "
		Computerized adaptive testing-substance use disorder (CAT-SUD)[b]	ED patients between 18–70 years
		Dynamic Assessment and Referral System for Substance Abuse (DARSSA)[b]	

Table S4. (Continued)

Topic	Subtopic	PROMs	Applied to ED patients (otherwise specified)
		**Generic category**	
**Substance use**	**General**	National Institute of Drug Abuse (NIDA) Quick Screen Questionnaire[c]	(i) ED patients (ii) ED trauma patients (iii) ED pregnant women
		Two-Item Conjoint Screen for Alcohol and Other Drug Problems (TICS)	ED patients with mental health problems
		**Disease specific category**	
**Asthma**		Acute Asthma Quality of Life Questionnaire (AQLQ)	ED patients with acute severe broncho-constriction
		AERO Study Questionnaire	ED patients with asthma exacerbation
		Asthma Symptom Severity	ED patients with acute severe broncho-constriction
**Concussion**		Rivermead Post-Concussion Symptoms Questionnaire (RPQ)	Patient with minor injury
**HIV**		HIV RE&IC questionnaire	ED patients and primary care patients
**Palliative care**		Screening for Palliative Care Needs in the Emergency Department (SPEED) instrument	ED patients with cancer
		**Symptom specific category**	
**Dyspnea**		5-point Likert scale	ED patients with dyspnoea suspected to be due to acute heart failure
		Modified Borg scale (MBS)	ED patients with asthma and COPD
		Provocative Dyspnea Severity Score (pDS)	ED patients with dyspnoea suspected to be due to acute heart failure
		Verbal dyspnoea scores (VDS)	ED patients with shortness of breath (SOB)
		Visual analogue scale (10 cm)	ED patients with dyspnoea suspected to be due to acute heart failure
**Hearing loss**		Numeric Rating Scale (NRS)	ED patients suspected of suffering from unilateral sudden sensorineural hearing loss (SSNHL)
**Nausea**		Adjectival scale	ED patients with nausea
		Muth Nausea Profile (Nausea descriptors)	ED patients with most likelihood of nausea, with the following chief complaints: abdominal pain, nausea, vomiting, nausea and vomiting (combined), headache, sore throat, and fever
		Numerical rating scale (NRS)	ED patients with nausea
		Visual Analogue scale (VAS)	ED patients with nausea

Table S4. (Continued)

Topic	Subtopic	PROMs	Applied to ED patients (otherwise specified)
		**Symptom specific category**	
**Pain**	**Back pain**	Cassandra rule	ED patients with non-specific back-pain
		Cassandra (5-item version)	ED patients with non-specific back-pain
		Roland Morris Disability Questionnaire (RMDQ)	ED patients with acute non-specific low back pain
		Roland Morris 5-item scale	ED patients with chief complaint of low back pain whose cause, in the judgment of the ED attending physician, was musculoskeletal
		STarT Back Screening Tool (SBST)	(i) ED patients with acute non-specific low back pain (ii) Patients with acute low back pain
	**Chest pain**	Acute Coronary Syndromes (ACS) Symptom Checklist	ED patients with symptoms triggering an acute coronary syndrome evaluation and who were ≥21 years
		Rose Angina Questionnaire (RAQ)	ED patients with chest pain aged 40 to 70 years
		Self-reported Emergency Department Assessment of Chest Pain Score (S-EDACS)	ED patients presenting with complaints of chest pain or tightness
	**General**	Brief Pain Inventory (BPI)	ED patients with cancer
		Colour Analogue Scale (CAS)	ED patients with pain
		Numeric Rating Scale (NRS)	(i) ED patients with (acute) pain (ii) all ED patients (iii) ≥ 65 after a motor vehicle collision (iv) ED patients with acute non-specific low back pain
		Pain [a]	ED patients with cancer and pain
		Pain Catastrophizing Scale (PCS)	ED patients with cancer
		Verbal numeric rating scale (VNRS)	ED patients with acute pain
		Verbal Rating Scale (VRS)	ED patients with limb trauma
		Visual Analog Scale (VAS)	(i) ED patients (ii) ED patients with (acute) pain

[a] PROMs with no identified name in the study.

[b] Computerized Adaptive Testing (CAT) PROMs

[c] Utilized as both a PROM and a CAT

Patient groups are denoted by Roman numerals (i–iv) to reflect the diversity of populations assessed across the included studies.
