# Supplementary material for: Patient-Reported Outcome Measures in the Emergency Department: A Scoping Review
Source: J Am Coll Emerg Physicians Open. 2026 Aug 17;7(5):100486. doi: 10.1016/j.acepjo.2026.100486 (PMC13520099; doi:10.1016/j.acepjo.2026.100486)
Supplement: Supplementary file 5 [file mmc5.pdf]

**Table S5: Characteristics of included PROM by topic, subtopic, content, purpose and number of items (in alphabetical order)**

| PROM | Topic | Sub-topic | Content[d] | Purpose | Number of items | Study/Author |
|---|---|---|---|---|---|---|
| 0 - to 10 -point rulers[a] | Mental health | Suicide | Seven rulers ranging from a global well-being item to items on sadness, hopelessness, wish to sleep and not wake up, wish to be dead, wish to kill self, and how suicidal the patient feels. | Discriminative | 7 | O'Connor et al.[1] |
| 5-point Likert scale | Dyspnea | | Dyspnea Assessment Instrument | Discriminative | 1 | Greene et al.[2] |
| 6-item PTSD Checklist – short version | Mental health | PTSD | Pre-injury posttraumatic stress symptoms were assessed at baseline using a short version of the Posttraumatic Stress Disorder (PTSD) Checklist for DSM-IV with the introduction "Below is a list of problems and complaints that people sometimes have in response to stressful life experiences. … choose the best response to indicate how much you have been bothered by that problem in the past month BEFORE YOUR INJURY" | Discriminative | 6 | Pacella LaBarbara et al.[3] |
| **A** | | | | | | |
| A 27 item self-report questionnaire[a] | Older people | General | A 27-item self-report questionnaire, completed as part of the ED interview, included age, sex, and other social and demographic variables; premorbid functional status (before the problem that brought the patient to the ED); change in function (since the current medical problem); a history of chronic diseases and falls; presence of sensory impairment(s) and mental problems; use of medications and | Predictive | 27 | McCusker et al.[4] |

| | | | | | | |
|---|---|---|---|---|---|---|
| | | | alcohol; recent hospital admissions and ED visits; and perceived general health status. The 6 ISAR questions are included in the questionnaire. | | | |
| A three-item tool for a homelessness risk screening[a] | Social risk | | A three-item tool (use of a shelter or drop-in center in the past 12 months, application for shelter in the past 3 months, and/or lifetime history of incarceration or jail stay, with any "yes" representing a "positive" screen) | Predictive | 3 | Doran et al.[5] |
| Abuse Assessment Screen (ASS) | Partner violence | | The AAS questions include physical violence questions and a safety question. Also included is a specific question about pregnancy and sexual assault. | Discriminative | 5 | Weiss et al.[6] |
| Acute Asthma Quality of Life Questionnaire (AQLQ) | Asthma | | The questionnaire is in an interviewer-administered format, asks patients to recall how they have felt during the previous half hour. | Evaluative | 11 | Juniper et al.[7] |
| Acute coronary syndromes (ACS) Symptom Checklist | Pain | Chest pain | The ACS Symptom Checklist is a 13-item validated instrument that measures symptoms of ACS. | Discriminative and predictive | 13 | Devon et al.[8] |
| Ada[b] | Health and symptoms | Sympto ms | Patients enter factors such as sex, age, and specific risk factors (hypertension, diabetes, smoking, and pregnancy), followed by their most troubling symptoms, which were the reasons for their ED visit. The app then proceeds through an adaptive question flow, asking the essential next questions to lead to optimal condition suggestions and urgency advice. | Discriminative | - | Cotte et al.[9]<br>Faqar-Uz-Zaman et al.[10]<br>Fraser et al.[11] |
| Adjectival scale | Nausea | | Assessment of nausea: — circling one of "none," "mild," "moderate," or "severe." | Discriminative and evaluative | 1 | Meek et al. [12] |

| | | | | | | |
|---|---|---|---|---|---|---|
| AERO Study Questionnaire | Asthma | | To identify factors associated with multiple emergency department (ED) visits. | Discriminative and predictive | 20 | Vaittinada Ayar et al. [13] |
| Alcohol Dependence questionnaire[a] | Substance use | Alcohol | Designed to provide a current rather than a life-time diagnosis of alcohol dependence using easily remembered and administered items that have a yes/no answer format that reduces ambiguity and enhances ease of scoring | Discriminative | 5 | Singh et al [14] |
| Alcohol, Smoking and Substance Involvement Screening Test (ASSIST) | Substance use | General | The ASSIST is a long-standing measure with subscales to detect nonmedical use of opioids (including prescription) as well as tobacco, nicotine (vaping), cannabis (including vaping), alcohol, cocaine, amphetamines, inhalants, sedatives, and hallucinogens | Discriminative | 8 | McCabe et al. [15]<br>Punches et al. [16]<br>Tuli et al. [17]<br>Van der Westhuizen et al. [18] |
| Alcohol use disorder identification test (AUDIT) | Substance use | Alcohol | AUDIT was developed by the World Health Organization to identify problem drinkers in primary care settings. AUDIT covers three conceptual domains of level of consumption, dependence symptoms, and alcohol-related consequences. | Discriminative | 10 | Borges et al.[19]<br>Cherpitel et al.[20]<br>Cherpitel et al. [21]<br>Chung et al. [22]<br>Cremonte et al. [23]<br>Fulbrook et al. [24]<br>Lee et al. [25]<br>Meneses-Gaya et al. [26]<br>Neumann et al. [27]<br>Shenvi et al. [28]<br>Brousse et al. [29] |
| Alcohol Use Disorders Identification Test - 3 (AUDIT-3) | Substance use | Alcohol | The shortest of these versions is the AUDIT-3, which consists solely of the third question of the AUDIT | Discriminative | 1 | Meneses-Gaya et al. [30] |
| Alcohol Use Disorders Identification Test - 4 (AUDIT-4) | Substance use | Alcohol | AUDIT- 4 consists of the first 3 questions plus question number 10 of the AUDIT | Discriminative | 4 | Meneses-Gaya et al. [30] |

| | | | | | | |
|---|---|---|---|---|---|---|
| Alcohol Use Disorders Identification Test - Consumption (AUDIT-C) | Substance use | Alcohol | AUDIT-C comprises the first 3 items among the 10 items of AUDIT. | Discriminative | 3 | Lee et al. [31] Meneses-Gaya et al. [30] Rodriguez-Martos et al. [32] |
| Alcohol Use Disorders Identification Test-Quantity-Frequency (AUDIT-QF) | Substance use | Alcohol | AUDIT-QF includes the first 2 questions of the AUDIT | Discriminative | 2 | Meneses-Gaya et al. [30] |
| Alcohol Use Disorders Identification Test - primary care (AUDIT-PC) | Substance use | Alcohol | AUDIT-PC comprises questions 1, 2, 4, 5, and 10 of the AUDIT | Discriminative | 5 | Meneses-Gaya et al. [30] |
| Asthma Symptom Severity | Asthma | | Patients were asked the question "How is your asthma now?" | Evaluative | 1 | Juniper et al. [7] |
| Australian modified version of Paddington Alcohol Test (PAT) (AusPAT) | Substance use | Alcohol | AusPAT is a modification of the 2003 version of PAT. | Discriminative | 4 | Fulbrook et al. [24] |
| **B** | | | | | | |
| Barratt Impulsiveness Scale (BIS) | Mental health | General | BIS is a questionnaire designed to measure a person's level of impulsiveness using a 4-point rating scale. The questionnaire provides several subscales that attempt to measure specific aspects of impulsiveness including attentional, attentional impulsiveness, cognitive complexity, cognitive instability, motor, motor impulsiveness, nonplanning impulsiveness, perseverance, and self-control. | Discriminative and predictive | 30 | Randall et al. [33] |

| | | | | | | |
|---|---|---|---|---|---|---|
| Beck Depression Inventory II (BDI-II) | Mental health | Depression | BDI-II is used to screen for the presence and severity of depressive symptoms | Discriminative | 21 | Houry et al. [34] |
| Beck Hopelessness Scale (BHS) | Mental health | General | The BHS is a true or false questionnaire that attempts to measure the level of hopelessness that a patient has about their future. | Discriminative and predictive | 2 | Randall et al. [33] |
| Becks scale for suicide ideation (BSS)[c] | Mental health | Suicide | The screening items assess a participant's wish to live or die and the strength of the desire to commit suicide. | Discriminative | 21 | Boudreaux et al. [35 c]<br>Chang et al. [36]<br>Houry et al. [34] |
| Brief-illness Perception Questionnaire (B-IPQ) | Health and symptoms | Impact of illness | B-IPQ assess consequences (impact of illness on one's life, item 1), timeline (acute/chronic—perception of the length of illness and item 2), personal control (the individual's control over the symptoms/illness, item 3), treatment control (perception of effect of treatment, item 4), identity (the label used to describe the illness or symptoms, item 5), illness concern (concern about the illness, item 6), illness coherence (the individual's understanding of the illness, item 7) and emotions (emotional reactions to illness, item 8). Furthermore, an open-ended qualitative question allowing respondents to articulate their own perceptions of their condition may be incorporated. | Discriminative | 8 | Jensen et al. [37] |
| Brief mental health screen | Partner violence | | It provides emergency clinicians with more information about patients who are intimate partner violence (IPV) positive. | Discriminative | 4 | Houry et al. [34] |

| | | | | | | |
|---|---|---|---|---|---|---|
| Brief Michigan Alcohol Screening test (BMAST) | Substance use | Alcohol | The BMAST questionnaire is used to detect alcoholism. | Discriminative | 10 | Borges et al.[19]<br>Cremonte et al. [23] |
| Brief Pain Inventory (BPI) | Pain | General | BPI assesses pain intensity over the preceding 7 days by querying patients regarding their worst, least, and average pain experiences, in addition to current pain levels. These four ratings are then averaged to generate a composite pain severity score. Furthermore, the BPI incorporates ten items designed to quantify the extent to which pain interferes with various aspects of daily functioning. | Discriminative and evaluative | 10<br><br>(BPI short version - 4 items) | Azizoddin et al. [38] |
| Brief Risk Identification for Geriatric Health Tool (BRIGHT) | Older people | General | BRIGHT screen is designed to quickly identify those older adults who would benefit most from a comprehensive geriatric assessment.<br><br>The items address the following common geriatric issues: help with bathing, personal hygiene, dressing the lower body, getting around indoors, difficulty making decisions about everyday activity, shortness of breath, recent falls, perception of general health, memory problems, ability to do ordinary housework, and depression. | Discriminative | 11 | Boyd et al. [39] |
| Brief Screening Instrument for Adult Psychopathology [a,b] | Mental health | General | An instrument that assesses areas of behavioral health.<br>Covers 10 domains of emotional and/or behavioral difficulties: Major depressive disorder/dysthymia, Panic, PTSD, Social anxiety, Mania/hypomania, Obsesive Compulsive Disorder, Generalized anxiety disorder, Suicidality, Drug use, Alcohol use. | Discriminative | 12-28<br><br>Completion time: less than 9 min. | Sattler et al. [40] |

| | | | | | | |
|---|---|---|---|---|---|---|
| Brief screen tool for substance use detection[a] | Substance use | Drugs | Two single-item screening questions to detect illicit substance use, one for marijuana and one for other illicit drugs.<br>'In the past 3 months, have you 1) used any street drugs and 2) used any marijuana?' | Discriminative | 2 | Broderick et al.[41] |
| Brief Symptom Inventory (BSI) | Mental health | General | BSI assesses various psychiatric symptoms present in the past week using a 5-point rating scale. Subscales; anxiety, depression, hostility, interpersonal sensitivity, obsessive–compulsivity, paranoid ideation, phobic anxiety, psychoticism, and somatization. | Discriminative and predictive | 53 | Randall et al. [33] |
| **C** | | | | | | |
| Carpenter | Older people | Fall | Carpenter assesses fall risk by evaluating the presence of four risk factors – nonhealing foot sores, self-reported depression, inability to self-clip toenails, and prior falls. | Predictive | 4 | Curiati et al. [42] |
| Cassandra rule | Pain | Back pain | Used to predict back pain. Items from the SCL90-R were used to measure symptoms of depression and somatization. Other questions drawn from the Psychological Symptom Index (PSI) and the Brief Symptom Inventory (BSI) were also included. | Predictive | 17 | Dionne et al. [43] |
| Cassandra (5-item version) | Pain | Back pain | Questions included in the Cassandra rules for the prediction of long-term, severe, back-related functional limitations. | Predictive | 5 | Dionne et al. [43] |
| Catastrophizing subscale of the Coping Strategies Questionnaire (CSQ) | Mental health | General | The original CSQ, a 42-item self-report scale, was developed to assess the use of six cognitive coping strategies and one behavioral strategy in a sample of patients with chronic pain. | Predictive | 6 | Reyes-Gibby et al. [44] |

| | | | | | | |
|---|---|---|---|---|---|---|
| | | | The Catastrophizing subscale of the CSQ has six items. | | | |
| Center for Epidemiologic Studies–Depression (CES-D) scale | Mental health | Depress ion | CES-D is a self-report scale designed to measure depressive symptomatology in the general population. | Discriminative | 20 | Reyes-Gibby et al. [44] |
| Clinical Frailty Scale | Older people | Frailty | CFS is a global clinical measure of fitness and frailty that uses pictographs and clinical descriptions to help clinicians stratify older adults on a nine-point scale. | Discriminative | 1 | Dresden et al. [45] |
| Color Analog Scale (CAS) | Pain | General | CAS is a 100-mm-long triangular shape. Its width ranges between 10 mm at the lower anchor ‘‘no pain’’ and 30 mm at the upper anchor ‘‘worst possible pain.’’ CAS is also color graduated from white (lower anchor) to dark red (upper anchor). | Discriminative and evaluative | 1 | Bahreini et al. [46] |
| Columbia Suicide Severity Scale (C-SSRS) | Mental health | Suicide | C-SSRS is a risk scoring system that can be used to assist clinicians with their evaluation of patients in the Emergency Department in predicting overall risk and the need for hospital admission. | Discriminative and predictive | 6 | Chang et al. [36]<br>Simpson et al. [47] |
| COmplex NEeds Case-finding Tool – 6 (CONECT-6) | Social risk | | CONECT-6 can identify patients with complex health needs | Discriminative | 6 | Hudon et al. [48] |
| Computerized Adaptive Test-Depression Inventory (CAT-DI)[b] | Mental health | Depress ion | CAT-DI is a computerized adaptive dimensional severity measure for depression that utilizes a bank of 389 depression items whose response patterns are fitted to a multidimensional item response theory model. | Discriminative | 389<br><br>The average is 12 items. Completio | Beiser et al. [49]<br>Beiser et al. [50] |

| | | | | | | |
|---|---|---|---|---|---|---|
| | | | Patients receive on average 12 items with a median completion time of 2 minutes 17 seconds. | | n time: 2 minutes | |
| Computerized Adaptive Diagnostic Test for Major Depressive Disorder (CAD-MDD)[b] | Mental health | Depression | CAD-MDD is a computerized adaptive depression screening tool based on a random forest machine learning algorithm that adapts to patient responses to questions about depression by asking the most diagnostically informative question from an item bank. | Discriminative | 88<br><br>Completion time: less than 1 minute | Beiser et al. [49] |
| Computer adaptive test - mental health (CAT-MH)[b] | Mental health | General | CAT-MH is based on multidimensional item response theory and is suitable for the measurement of complex constructs such as depression, anxiety, and suicidality. | Discriminative | 1500<br><br>Completion time: 2 minutes per module | O'Reilly et al.[51] |
| Computerized adaptive testing-substance use disorder (CAT-SUD)[b] | Substance use | General | The CAT-SUD design involves adaptive measurement that includes a fixed set of substance-specific questions (administered randomly throughout the testing session) that inquiries about use and frequency of (1) opiates and analgesics, (2) alcohol, (3) cocaine and amphetamines, (4) heroin or methadone and (5) sedatives (sedatives, hypnotics, tranquilizers, barbiturates), with accompanying lists of specific substances.<br>The CAT-SUD can accurately measure SUD severity with an average of 11 items in approximately 2 minutes. | Discriminative | 252<br><br>The average is 11 items. Completion time: 2 minutes | Gibbons et al. [52] |

| | | | | | | |
|---|---|---|---|---|---|---|
| Computerized Alcohol Screening and brief Intervention (CASI)[b] | Substance use | Alcohol | CASI provides a short, self-administered questionnaire. It administers the Alcohol Use Disorders Identification Test (AUDIT), with logic branching to shorten the screening for non-drinkers and those who drink less than the NIAAA-recommended thresholds. At the end of the questionnaire, CASI selects an appropriate plan with referral for treatment if indicated. | Discriminative | 10<br><br>Completion time: 2-4 minutes | Lotfipour et al. [53] |
| Computerized Assessment and Referral System (CARS)[b] | Mental health | General | A self-administered computerized screener adapted from the Composite International Diagnostic Interview. CARS identifies up to 16 major psychiatric disorders.<br><br>After completion, the screener generates a printout that identifies both positive lifetime and past-year diagnoses as well as more detailed information about each disorder identified. | Discriminative | -<br><br>Completion time: 10-15 minutes | Thompson et al. [54] |
| Convergent Functional Information for Suicidality (CFI-S) | Mental health | Suicide | CFI-S is a checklist of risk factors for suicidality from a variety of domains including life satisfaction, mental health, physical health, environmental stress, addictions, cultural factors, and demographic information. | Discriminative and predictive | 22 | Brucker et al. [55] |
| Current Opioid Misuse Measure (COMM) | Substance use | Drugs | COMM assesses current opioid misuse in chronic pain patients receiving opioid therapy but does not predict future risk. The 17-item questionnaire focuses on opioid use behaviors within the past 30 days. | Discriminative | 17 | Chalmers et al. [56] |

| | | | | | | |
|---|---|---|---|---|---|---|
| Cut down, Annoyed, Guilt, Eye-opener (CAGE) | Substance use | Alcohol | CAGE was designed for screening for alcohol dependence on a lifetime basis in clinical practice, and is derived from the following four items: (1) Have you ever felt you should cut down on your drinking? (2) Have people annoyed you about your drinking? (3) Have you ever felt bad or guilty about your drinking? (4) Have you ever had a drink first thing in the morning to steady your nerves or get rid of a hangover (eye-opener)? | Discriminative | 4 | Borges et al. [19]<br>Randall et al. [33]<br>Trautman et al. [57]<br>Cherpitel et al. [21]<br>Cremonte et al. [23]<br>Friedmann et al. [58]<br>Shenvi et al. [28]<br>Brousse et al. [29] |
| **D** | | | | | | |
| Degree-of-worry (DOW) scale | Mental health | General | DOW askes 'How worried are you about the condition that brings you here today, on a scale from 1 to 10, where 1 is minimal worried and 10 is maximum worried?' | Discriminative | 1 | Gamst-Jensen et al. [59] |
| Diagnostic interview schedule (DIS) | Substance use | Alcohol | The first DIS question was, 'In the last year, have you had 6 or more drinks?' Participants who twice denied drinking 6 drinks in the entire previous year were not asked the remaining DIS questions. The next DIS question addressed the frequency of drinking. The third DIS question asked the 'average' number of drinks per occasion. | Discriminative | 3 | Canagasaby et al. [60] |
| Drug Abuse Screening Test (DAST-10) | Substance use | Drugs | The DAST-10 questionnaire is used to detect at-risk drug users for substances other than alcohol or tobacco. Questions relate to consumption, tolerance, dependence, and consequences of drug use with an emphasis on questions relating to consequences. | Discriminative | 10 | Randall et al. [33] |

| | | | | | | |
|---|---|---|---|---|---|---|
| DSM-5 Criteria for Diagnosis of Opioid Use Disorder (survey checklist) | Substance use | Drugs | A survey checklist adapted from the 5th Edition of the Diagnostic and Statistical Manual of Mental Disorders (DSM-5) criteria, adapted with (Yes/no) | Discriminative | 11 | Punches et al. [16] |
| Dynamic Assessment and Referral System for Substance Abuse (DARSSA)[b] | Substance use | General | The DARSSA is comprised of three integrated modules: (1) a computerized assessment of substance use and other psychosocial variables, (2) a report generator, and (3) a referral generator. The assessment module provides for the self-administered assessment of tobacco, alcohol, illicit drug, and licit drug use and abuse. For each substance use category (tobacco, alcohol, and drugs), the DARSSA assesses lifetime use, use in the past 30 days, the amount of use, last use, dollars spent, substance-related problems and consequences, readiness to change, readiness to enter treatment, and withdrawal symptoms. In addition to substance use and abuse, the DARSSA assesses (1) demographic and socio-economic variables (2) general health status (3) psychiatric history (4) current mental health symptoms and (5) recent interpersonal victimization. | Discriminative | - Completion time: 12min | Boudreaux et al. [61] |
| **E** | | | | | | |
| ED-DSI | Older people | Depression | ED-DSI is a screening instrument to detect depression in ED elders | Discriminative | 3 | Fabacher et al. [62] |

| | | | | | | |
|---|---|---|---|---|---|---|
| EuroQol 5 Dimensions (EQ-5D) | Health and symptoms | General health | The EQ-5D value health by deriving a utility score from individual responses to 5 questions measuring severity in mobility, self-care, usual activities, pain/discomfort, and anxiety/depression. Is also includes a VAS-scale on general health, from 0-100 | Discriminative | 6 | Clemens et al. [63] |
| Expectation questionnaire[a] | Expectati on | | The tool includes questions asking the patient where they expected to be dispositioned, their reason for presenting to the ED, and what expectations they had regarding potential treatment medications and interventions. | Discriminative | 5 | Chapman et al. [64] |
| Extended - Hurt, Insult, Threaten, Scream (E-HITS) | Partner violence | | The HITS is an instrument that asks female patients about their experiences with IPV and the frequencies. The items cover the main areas experienced by victims who are abused by verbal and physical aggression by their spouses. Since HITS does not cover sexual violence, one more question to detect the presence of sexual violence was added. | Discriminative | 5 | Chan et al. [65] |
| **F** | | | | | | |
| Falls Efficacy Scale (FES) | Older people | Fall | FES is a tool that assesses fall-related self-efficacy and fear of falling, which may lead to a decline in physical fitness and an increase in fall risk due to physical frailty. | Discriminative and predictive | 10 | Greenberg et al. [66] |

| | | | | | | |
|---|---|---|---|---|---|---|
| Fast Alcohol Consumption Evaluation (FACE) | Substance use | Alcohol | Screenings for alcohol use disorder (AUD) at the Emergency Department<br><br>The FACE relies on the following items regarding the last 12 months: 1) How often have you had a drink containing alcohol?<br>2) How many drinks containing alcohol do you have on a typical day when you are drinking?<br>3) Have your friends and relatives worried or complained about your drinking?<br>4) Do you sometimes have a drink first gesture of the morning?<br>5) Have you ever had drinks and no longer remember what you said or did afterwards? | Discriminative | 5 | Airagnes et al. [67] |
| Fast alcohol screening test (FAST) | Substance use | Alcohol | FAST consists of question numbers 3, 5, 8, and 10 of the AUDIT. The first question of the FAST (item 3 of the AUDIT) was modified; the amount of drinks consumed in a single occasion is no longer the same for men and women (six or more), now reads "eight or more doses" for men and "six or more doses" for women | Discriminative | 4 | Knightly et al. [68] (2016)<br>Meneses-Gaya et al. [26]<br>Meneses-Gaya et al. [30] |
| Five-Shot | Substance use | Alcohol | The Five-Shot integrates questions 2, 3, and 4 of the CAGE with items 1 and 2 of the AUDIT | Discriminative | 5 | Meneses-Gaya et al. [30] |
| FRAIL scale (FS) | Older people | Frailty | The FS is comprised of five characteristics: fatigue, resistance, ambulation, illnesses and loss of weight. | Discriminative | 5 | Liu et al. [69]<br>Shang et al. [70] |

| | | | | | | |
|---|---|---|---|---|---|---|
| Frailty Screening Questionnaire (FSQ) | Older people | Frailty | The FSQ includes five self-reported components based on the modified Fried FP criteria: weight loss, exhaustion, slowness, weakness and inactivity. | Discriminative | 5 | Liu et al. [69]<br>Shang et al. [70] |
| **G** | | | | | | |
| General Anxiety Disorder questionnaire 2 item (GAD-2) | Mental health | Anxiety | GAD-2 is an initial screening tool for generalized anxiety disorder | Discriminative | 2 | Kene et al. [71] |
| Generalized Anxiety Disorder Scale (GAD-7) | Mental health | Anxiety | The GAD-7 measures severity of anxiety. | Discriminative | 7 | Shah et al. [72] |
| Geriatric Depression Scale (GDS-5) | Older people | Depress ion | GDS is a widely used screening tool to identify depression risk in geriatric patients. The GDS was originally a 30-item questionnaire (GDS-30), and it has been validated in a large sample of geriatric patients. | Discriminative | 5 | Shrestha et al. [73] |
| Geriatric Depression Scale (GDS-15) | Older people | Depress ion | GDS is a widely used screening tool to identify depression risk in geriatric patients.<br>GDS-15 has been validated and is now widely used. Each item covers the individual's experiences during the last week. The GDS is useful in the ED given that it has simple "yes or no" responses | Discriminative | 15 | Shrestha et al. [73] |
| General Health Questionnaire -12 item (GHQ-12) | Mental health | General | GHQ-12 is generally considered a unidimensional scale of psychological distress, consisting of items capturing symptoms of anxiety, depression, social dysfunction, and loss of confidence. | Discriminative | 12 | Vaittinada Ayar al. [13] |
| **H** | | | | | | |

| | | | | | | |
|---|---|---|---|---|---|---|
| HARK (Humiliation, Afraid, Rape, Kick) | Partner violence | | 4 items assess emotional and physical intimate partner violence (IPV) in the past year | Discriminative | 4 | Kene et al. [71] |
| Health literacy questionnaire by European Consortium (HL-SF12) | Health literacy | | The questionnaire includes items from a short version of the Health Literacy Questionnaire developed and validated by the European Consortium as part of the European Health Literacy (HLS-EU) project.<br>The 12 included questions assess health literacy across three domains: health promotion, disease prevention, and health care. Patients are asked to evaluate their ability to access, understand, and use health-related information within each of these domains. | Discriminative | 12 | Ahmed et al.[74] |
| Health-related social needs (HRSN) (modified) | Social risk | | HRSN questions were selected from validated instruments in consultation with experts in geriatric care. Five domains were examined: transportation, social isolation, financial strain, housing stability, and difficulty paying for medications. | Discriminative and predictive | 26 | Aylward et al. [75] |
| HITS ("Hurt/Insult/Threaten /Scream") | Partner violence | | HITS screens for IPV, with following questions: "How often does your partner: physically hurt you; Insult or talk down to you; Threaten you with harm; Scream or curse at you?" | Discriminative | 4 | Kene et al. [71]<br>Mills et al [76] |

| | | | | | | |
|---|---|---|---|---|---|---|
| HIV RE&IC questionnaire | HIV | | The questionnaire facilitates the identification of potential risks for HIV exposure or indicator conditions (IC) known to be associated with HIV acquisition.<br><br>The first six items of the HIV RE&IC questionnaire assess HIV-related exposures (RE), based on established routes of HIV transmission. The remaining 14 items evaluate HIV indicator conditions (IC), drawing on a selection of conditions identified in the HIV Indicator Diseases Across Europe Study. | Discriminative | 20 | Perez Elias et al. [77] |
| "Human trafficking screening tool" [a] | Sex trafficking | | Human trafficking screening tool for adults in the ED | Discriminative | 11 | Kaltiso et al. [78] |
| Hunger Vital Sign questionnaire (HVS) | Older people | Nutrition | The HVS questionnaire assesses food insecurity using respondents' level of agreement with two statements regarding the perceived risk and experience of food scarcity: "We worried whether our food would run out before we got money to buy more" and "The food we bought just did not last and we did not have money to get more." | Discriminative | 2 | Aylward et al. [75] |
| I | | | | | | |

| | | | | | | |
|---|---|---|---|---|---|---|
| Identification of Seniors At Risk (ISAR) | Older people | General | ISAR identifies older people (≥65 years) at risk of functional decline, mortality, readmission and institutionalisation.<br><br>ISAR consists of six assessment items: presence of home help, increased dependency, history of hospital admissions, visual problems, memory problems, and polypharmacy (≥3 drugs). | Discriminative and predictive | 6 | Gentile et al. [79]<br>Graf et al [80]<br>Bahadirli et al. [81]<br>Braes et al. [82]<br>Braes et al [83]<br>de Almeida Tavares et al. [84]<br>Demir Akça et al. [85]<br>Dendukuri et al. [86]<br>Heeren et al. [87]<br>Loddo [88] (2021)<br>McCusker et al. [89]<br>McCusker et al. [4]<br>Moons et al. [90]<br>Rizka et al. [91]<br>Rizka et al. [92]<br>Salvi et al. [93]<br>Salvi et al. [94]<br>Salvi et al. [95]<br>Singler et al. [96]<br>Suffoletto et al. [97]<br>Tavares et al. [98] |
| Identification of Senior At Risk Hospitalized Patients' (ISAR-HP) | Older people | General | ISAR-HP was developed in a Dutch cohort study of hospitalized patients. It includes items assessing instrumental activities of daily living (IADL) assistance, use of a walking aid, need for assistance with traveling, and education beyond the age of 14. | Discriminative and predictive | 4 | van Dam et al. [99]<br>van Dam et al. [100] |
| Injured trauma survivor screen (ITSS) | Mental health | PTSD | ITSS is a self-report measure of risk for PTSD and depression post-injury. | Predictive | 9 | Ennis et al. [101] |
| **L** | | | | | | |
| Life-threatening experience[a] | Mental health | PTSD | Patients were asked to rate how life-threatening they believed the motor vehicle collision to be on a scale of 0 to 10. | Predictive | 1 | Platts-Mills et al.[102] |
| **M** | | | | | | |

| | | | | | | |
|---|---|---|---|---|---|---|
| Malnutrition Screening Tool (MST) | Older people | Nutrition | MST is a screening tool used to identify individuals at risk of malnutrition who may require nutritional support and intervention. Patients are asked the following questions: “Have you recently lost weight without trying?,” “If yes, how much weight have you lost?” and “Have you been eating poorly because of a decreased appetite?” | Discriminative | 3 | Aylward et al. [75] |
| Medical Term Recognition Test (METER) | Health literacy | | METER consists of a list of 40 medical terms intermixed with nonwords. Patients are asked to identify the real words by marking them on a response sheet. | Discriminative | - | Kiechle et al. [103] |
| Mini Geriatric Depression Scale (Mini-GDS) | Older people | Depression | Screening for depression. | Discriminative | 4 | Gentile et al. [79] |
| Modified Borg scale (MBS) | Dyspnea | | The MBS is a vertical numerical scale ranging from 0 to 10, with each number anchored by a corresponding verbal descriptor of progressively increasing intensity. Patients were asked to rate their dyspnea by selecting the number and associated descriptor that best reflected their perceived level of breathlessness. | Discriminative | 1 | Kendrick et al. [104] |
| Muth Nausea Profile (Nausea descriptors) | Nausea | | The survey instrument consisted of 18 items, each rated on an 11-point Likert scale ranging from 0 to 10, assessing different subjective experiences of nausea | Discriminative and evaluative | 18 | Cloutier et al. [105] |
| **N** | | | | | | |

| | | | | | | |
|---|---|---|---|---|---|---|
| National Institute of Drug Abuse (NIDA) Quick Screen Questionnaire [c] | Substance use | General | The NIDA Quick Screen Questionnaire investigates an individual's use of alcohol, tobacco products, prescription drugs (without a prescription), and illegal drugs within the last year.<br><br>A computerized version of the National Institute of Drug Abuse (NIDA) Quick Screen Questionnaire.<br>If an individual answered anything other than "never" to either of the questions that ask about improper prescription or illegal drug use, extra survey questions asking about an individual's specific drug use were populated. The types of drugs in question included: cannabis, cocaine, prescription stimulants, methamphetamine, inhalants, sedatives/sleeping pills, hallucinogens, street opioids (i.e. heroin), and prescription opioids (Tuli et al.) | Discriminative | 4 | Kene et al. [71]<br>Tuli et al. [17]<br>White et al. [106] |
| National Institutes of Health (NIH) - recommended heavy drinking day item[a] | Substance use | Alcohol | How many times in the past year have you had 5 or more drinks in a day (4 or more for women)? | Discriminative | 1 | Stewart et al.[107] |
| Numerical rating scale (NRS) | Hearing loss | | A numerical rating scale (NRS) for assessing hearing loss serves as a tool to capture a patient's subjective estimation of the severity of the loss, ranging from no hearing loss to complete deafness. | Discriminative | 1 | Ungar et al. [108] |
| Numerical rating scale (NRS) | Nausea | | On the NRS, participants indicate their nausea by selecting a value from 0 ("no nausea") to 10 ("unbearable nausea"), with shifts to lower or higher numbers recorded as negative or positive changes, respectively. | Discriminative and evaluative | 1 | Meek et al. [12] |

| | | | | | | |
|---|---|---|---|---|---|---|
| Numerical rating scale (NRS) | Pain | General | Patients rate their pain ‘‘on a scale from 0 to 10, with 0 equal to no pain and 10 equal to worst possible pain’’ | Discriminative and evaluative | 1 | Al-Kalaldeh et al. [109]<br>Bahreini et al. [46]<br>Bijur et al. [110]<br>Hsu et al. [111]<br>Ku et al. [112]<br>Platts-Mills et al. [102]<br>Medeiros et al.[113] |
| **O** | | | | | | |
| Older Americans Resources and Services scale (OARS) | Older people | General | The Older Americans Resources and Services scale is a 28-point validated tool that quantifies patients' ability to perform seven activities of daily living (eating, dressing, grooming, walking, transfers, bathing, and using the toilet) and seven instrumental activities of daily living (using the telephone, driving or using public transportation, shopping, cooking, housework, managing medications, and managing finances). The tool was modified for self-assessment. | Discriminative | 14 | Boucher et al. [114]<br>McCusker et al. [89] |

| | | | | | | |
|---|---|---|---|---|---|---|
| Ongoing Abuse Screen (OAS) | Partner violence | | The OAS was developed specifically to address ongoing abuse:<br>1. Are you presently emotionally or physically abused by your partner or someone important to you?<br>2. Are you presently being hit, slapped, kicked, or otherwise physically hurt by your partner or someone important to you?<br>3. Are you presently being forced to have sexual activities?<br>4. Are you afraid of your partner or anyone of the following (circle if applicable): (husband/wife), (ex-husband/ex-wife), (boyfriend/girlfriend), stranger.<br>5. (If pregnant) Have you been hit, slapped, kicked, or otherwise physically hurt by your partner or someone important to you during pregnancy? | Discriminative | 5 | Weiss et al. [6] |
| Ongoing Violence Assessment Tool (OVAT) | Partner violence | | OVAT is an assessment tool for ongoing violence assessment tool. | Discriminative | 4 | Ernst et al. [115]<br>Weiss et al. [6] |
| Opioid Risk Tool (ORT) | Substanse use | Drugs | ORT is an instrument evaluating risk factors, including personal and familial substance use history. | Predictive | 5 | Chalmers et al. [56] |
| **P** | | | | | | |

| | | | | | | |
|---|---|---|---|---|---|---|
| Pain[a] | Pain | General | Pain was assessed using a 0-to-10 rating scale consisting of items such as "Best describes the amount of pain you are having now. (How strong is the pain?)," "At what pain level would you be comfortable," and "How much does pain interfere with your daily activities." Some items were aimed at determining the location of pain, and some dealt with pain treatment: "Are you currently being treated by or under the care of a health care provider for any problems with pain," "Approximately how many times have you visited a health care professional about your pain in the last year." | Discriminative | - | Reyes-Gibby et al. [44] |
| Pain Catastrophizing Scale (PCS) | Pain | General | To measure pain catastrophizing | Predictive | 13 | Azizoddin et al. [38] |
| Partner Violence Screen (PVS) | Partner violence | | The PVS consists of three questions about past physical violence and perceived personal safety. | Discriminative | 3 | Halpern et al. [116]<br>Houry et al. [117]<br>Mills et al. [76]<br>Trautman et al. [57] |
| Patient-centered Outcomes Questionnaire (PCOQ) | Health and symptoms | Impact of illness | PCOQ is a tool in which patients rate their usual, desired, expected, and successful levels across domains including pain, fatigue, emotional distress, and interference with daily activities. Patients also indicate the relative importance of improvement within each domain. The PCOQ was modified to incorporate two additional domains relevant to the emergency department population: sleep disturbances and difficulty concentrating. | Discriminative | 5 | Flynn et al. [118] |
| Patient Health Questionnaire (PHQ-2) | Mental health | Depression | PHQ-2 screens for depression as a first-step approach | Discriminative | 2 | Kene et al. [71]<br>Shenvi et al. [28] |

| | | | | | | |
|---|---|---|---|---|---|---|
| Patient Health Questionnaire-9 (PHQ-9) | Mental health | Depression | PHQ-9 is a self-reported screening tool for the identification of depression. | Discriminative | 9 | Chang et al. [36]<br>Hyphantis al. [119]<br>Shah et al. [72] |
| Patient Health Questionnaire-15 (PHQ-15) | Health and symptoms | Symptoms | PHQ-15 assesses 15 somatic symptoms or symptom clusters that together account for over 90% of physical complaints, excluding upper respiratory symptoms, reported in outpatient settings | Discriminative | 15 | Hyphantis et al. [120] |
| Patient Health Questionnaire – panic disorder module (PHQ-PD) | Mental health | Anxiety | PHQ-PD comprises four dichotomous (yes/no) screening items (Questions 3a–d; e.g., "In the last 4 weeks, have you had an anxiety attack—suddenly feeling fear or panic?") followed by an 11-item checklist of somatic symptoms (Questions 4a–k). These items are used to assess the presence of panic attacks as well as associated distress/ impairment. | Discriminative | 15 | Sung et al. [121] |
| Patient Reported Outcome Measurement Information System (PROMIS) short-forms | Mental health | General | PROMIS short-forms assess symptoms of depression, anxiety, and sleep disturbance. | Predictive | - | Azizoddin et al. [38] |
| Patient Reported Outcome Measurement Information System (PROMIS) Physical Function (PF) 10 item short form | Health and symptoms | General health | Self-reported measure of physical function | Discriminative | 10 | Fox et al. [122] |
| Patient Safety Screener (PSS) | Mental health | Suicide | PSS is a suicide risk screening instrument<br>PSS-2 covers two items<br>PSS-3 covers three items (depression item included) | Discriminative | 2-4 | Boudreaux et al. [123] |

| | | | | | | |
|---|---|---|---|---|---|---|
| Patient Self-Report Survey[a,b] | Mental health | Suicide | The self-report battery assesses established risk factors for suicide attempt, including sociodemographic characteristics; history of psychiatric symptoms, suicidal thoughts, and suicidal behaviors; family history of psychiatric problems; traumatic life event history; psychological traits; and psychiatric treatment history. | Predictive | 67-97<br><br>Completion time: 11 minutes | Nock et al. [124] |
| Penn Richmond screener | Mental health | PTSD and depression | The Penn Richmond Screener is a brief instrument that can be administered in approximately 1–2 minutes. It comprises four items assessing depressive symptoms and five items assessing posttraumatic stress disorder (PTSD) symptoms, with one item shared across both domains. | Predictive | 8 | Kumodzi et al. [125] |
| Peritraumatic Dissociative Experiences Questionnaire (10-items)-Self-Report Version (PDEQ-10-SRV) | Mental health | PTSD | PDEQ-10-SRV assesses the presence of peritraumatic dissociation. | Discriminative | 10 | Birmes et al. [126]<br>Pacella-LaBarbara et al. [3] |
| Peritraumatic distress inventory (PDI) | Mental health | PTSD | PDI is a self-report measure. | Predictive | 13 | Ennis et al. [101]<br>Platts-Mills et al. [102] |
| PErpetration RaPid Scale (PERPS) | Partner violence | | PERPS is a short scale to detect perpetration of IPV. | Discriminative | 3 | Ernst et al. [127] |
| Posttraumatic Adjustment Scale (PAS) | Mental health | PTSD and depression | PAS is assessing pretrauma, peritrauma, and posttrauma risk factors to screen for the likelihood of developing posttraumatic stress disorder (PTSD) and depression following acute trauma | Predictive | 10 | Jaramillo et al. [128]<br>Kumodzi et al. [125] |

| | | | | | | |
|---|---|---|---|---|---|---|
| Posttraumatic Stress Diagnostic Scale (PDS) | Mental health | PTSD | PDS are designed to assess three categories of symptoms associated with posttraumatic stress disorder (PTSD): re-experiencing, avoidance, and hyperarousal. | Discriminative | 17 | Houry et al. [34] |
| Poverty-related Quality of Life (PQOL-17) | Social risk | | PQOL-17 is an original tool that may be used in clinical routine practice to detect particularly vulnerable individuals to improve treatment effectiveness. | Discriminative | 17 | Boyer et al. [129] |
| Pre-injury psychological treatment[a] | Mental health | PTSD | "Has there ever been a point in your life when you received help for emotional or mental health problems?" | Predictive | 1 | Pacella-LaBarbara et al.[3] |
| Primary Care Evaluation of Mental Disorders (PRIME-MD)[c] | Mental health | | PRIME-MD is a validated diagnostic instrument developed to address barriers to the identification of psychiatric disorders in primary care settings. In its original format, the PRIME-MD comprised a 27-item, patient-administered screening questionnaire assessing five clinical domains. Positive responses within a given domain prompted the clinician to conduct a structured, domain-specific interview.<br>Subsequently, both the screening and interview components were adapted for patient self-administration, and a computerized version was developed. The computer-based version includes diagnostic modules for mood, anxiety, alcohol use, eating, obsessive-compulsive, phobic, and somatoform disorders. Upon completion of the assessment, the program generates a one-page summary listing all diagnosable conditions within its | Discriminative | 27 | Platts-Mills et al. [102]<br>Schriger et al. [130] |

| | | | | | | |
|---|---|---|---|---|---|---|
| | | | scope, with confirmed diagnoses clearly indicated. | | | |
| Program of Research on the Integration of Services for the Maintenance of Autonomy (PRISMA-7) | Older people | General | PRISMA-7 tool categorizes older people into two risk strata for disability: low versus high risk. Demonstrating robust sensitivity, it effectively identifies frailty within community-dwelling older populations. | Discriminative and predictive | 7 | Beauchet et al. [131]<br>Jarman et al. [132] |
| Provocative Dyspnea Severity Score (pDS) | Dyspnea | | pDS is a dyspnea assessment instrument using a 9-point scale. | Discriminative and evaluative | 1 | Greene et al. [2] |

| | | | | | | |
|---|---|---|---|---|---|---|
| Psychiatric Emergency Research Collaboration (PERC screener) | Mental health | Suicide | PERCH is a screening instrument was developed by adapting questions from two established measures: the Patient Health Questionnaire-9 (PHQ-9) and the Columbia Suicide Severity Rating Scale (SSRS). The first two questions from the PHQ9 are probes for depression and loss of interest consistent with the diagnostic criteria for major depression as outlined in the Diagnostic and Statistical Manual of Mental Disorders (DSM-IV; American Psychiatric Association, 2000). The remaining three items were derived from the SSRS, with two addressing passive and active suicidal ideation. The final item assessed lifetime history of suicide attempts via the direct question, "Have you ever attempted to kill yourself?" If a prior attempt was reported, the time elapsed since the most recent attempt was subsequently ascertained | Discriminative | 5-6 | Allen et al. [133] |
| Psychiatric diagnostic screening questionnaire – panic disorder module (PDSQ-PD) | Mental health | Anxiety | PDSQ-PD can be used to assess the presence of panic symptoms (e.g., "During the past two weeks did you get very scared because your heart was beating fast?"). | Discriminative | 8 | Sung et al. [121] |
| **R** | | | | | | |
| Rapid Alcohol Problems Screen (RAPS) | Substance use | Alcohol | RAPS exhibits good sensitivity to identify individuals currently meeting the diagnosis for alcohol dependence | Discriminative | 5 | Cherpitel [134] |

| | | | | | | |
|---|---|---|---|---|---|---|
| Rapid Alcohol Problems Screen (RAPS4) | Substanc e use | Alcohol | RAPS4 has been validated in EDs for the detection of alcohol use disorders. An individual who provides a positive response to any one of the four questions is considered positive. | Discriminative | 4 | Cherpitel [134]<br>Cherpitel et al. [135]<br>Cherpitel et al. [20]<br>Cherpitel et al. [21]<br>Cremonte et al. [23]<br>Stewart et al. [107] |
| Rapid Alcohol Problems Screen – Quantity Frequency (RAPS4-QF) | Substanc e use | Alcohol | RAPS4-QF is a screening instrument for harmful drinking and abuse in the general population and in ED populations<br><br>The RAPS4-QF consists of the RAPS4 questions plus two additional items: quantity and frequency | Discriminative | 6 | Cherpitel [135]<br>Cherpitel et al. [20]<br>Cherpitel et al. [21]<br>Cremonte et al. [23]<br>Brousse et al. [29] |
| Rapid drug problems screen (RDPS) | Substanc e use | Drugs | RDPS is a modified version of the RAPS4, adapted for the assessment of drug abuse and dependence. Patients reporting positive on one or more items were considered to screen positive. | Discriminative | 4 | Cherpitel et al. [136] |
| Readiness to Change Questionnaire [Treatment Version] (RCQ[TV]) | Substanc e use | Alcohol | RCQ[TV] was designed for use with treatment-seeking populations.<br><br>The RCQ[TV] reflects Stages of Change for both reducing and abstaining from drinking and thus may better capture the diverse goals of non-treatment-seeking populations. | Discriminative | 12/15 | Richards et al. [137] |

| | | | | | | |
|---|---|---|---|---|---|---|
| Rivermead Post-Concussion Symptoms Questionnaire (RPQ) | Concussion | | RPQ assesses the presence and severity of the following post-concussion (PC) symptoms: headaches, dizziness, nausea or vomiting, noise sensitivity, sleep disturbance, fatigue, irritability, depression, frustration, forgetfulness, poor concentration, slow thinking, blurred vision, light sensitivity, double vision, and restlessness. | Discriminative and predictive | 16 | McLean et al. [138] |
| Roland Morris Disability Questionnaire (RMDQ) | Pain | Back pain | The RMDQ is used to assess the level of disability associated with LBP. The RMDQ has items related to common activities that patients might have difficulty due to LBP | Discriminative | 24 | Medeiros et al.[113] |
| Roland Morris 5-item scale | Pain | Back pain | A short version of the Roland Morris Disability Questionnaire | Discriminative | 5 | Friedman et al. [139] |
| Rose Angina Questionnaire (RAQ) | Pain | Chest pain | RAQ is a validated tool used in a variety of clinical settings for the diagnosis of ischemic heart disease including myocardial infarct. | Discriminative | 2 | Poudel et al. [140] |
| Rowland | Older people | General | The Rowland questionnaire assess a patient's functional status, with the aim of identifying patients at risk for ED readmission. Items of the questionnaire address the use of walking aids or assistance when walking or transferring, assistance in dressing, dependency for collecting pension, dependency for shopping, use of daycare centre, use of meals-on-wheels and the presence of home help. | Predictive | 7 | Moons et al. [90] |
| **S** | | | | | | |

| | | | | | | |
|---|---|---|---|---|---|---|
| SAFE-T | Partner violence | | SAFE-T questionnaire is used to screen for intimate partner violence in an emergency department setting<br><br>The issues covered by these items were (a) **S**ecure/comfortable in home/apartment, (b) **A**ccepted by husband/partner, (c) **F**amily likes husband/ partner, (d) **E**ven/calm disposition of husband/partner, and (e) **T**alk to resolve differences. | Discriminative | 5 | Fulfer et al. [141] |
| SARC-F | Older people | General | SARC-F questionnaire has been found to be highly sensitive for the detection of frailty in older adults.<br>It comprises 5 components: strength, assistance with walking, rise from a chair, climb stairs, and falls. | Discriminative and predictive | 5 | Chong et al. [142] |
| Screener and Opioid Assessment for Patients with Pain-Revised (SOAPP-R) | Substance use | Drugs | SOAPP-R is a questionnaire designed to predict medication-related behaviors in individuals with chronic pain. It enquires about substance abuse and psychiatric history, medication related behaviors, doctor–patient relationship factors, lifestyle issues, and emotional attachment to pain medications. | Discriminative and predictive | 24 | Chalmers et al. [56]<br>Reyes-Gibby et al. [44]<br>Weiner et al. [143] |
| Screener for Intensifying Community Referrals for Health (SINCERE) | Social risk | | SINCERE is a screening tool inquiring about needs related to mortgage or rent, utilities, food security, household items, unstable housing, medical expenses, transportation, employment status, medication expenses, and childcare or eldercare. | Discriminative | 10 | Guo et al. [144] |

| | | | | | | |
|---|---|---|---|---|---|---|
| Screening for Palliative Care Needs in the Emergency Department (SPEED) instrument | Palliative care | | SPEED instrument is a brief, multidimensional symptom assessment tool developed to comprehensively and rapidly evaluate key domains of palliative care within the ED setting. Designed as a 'first-pass' assessment for ED clinicians, the SPEED instrument facilitates the identification of palliative care needs necessitating intervention, whether during the ED visit, through inpatient hospitalization, or in subsequent follow-up care | Discriminative | 13 | Richards et al. [145] |
| Screening survey[a] | Sex trafficking | | A screening survey based on published recommendations which could be administered in 5-10 minutes during the ED visit. | Discriminative | 14 | Mumma et al. [146] |
| Screening Test for At-risk Drinking (STAD) | Substanc e use | Alcohol | STAD is derived from AUDIT (Q3 and Q7), and screen for at-risk drinking in the ED. | Discriminative | 2 | Bae et al. [147]<br>Chung et al. [22] |
| Self-perceived risk of future homelessness[a] | Social risk | | Two single-item screening questions for self-perceived risk of future homelessness: (1) "Are you worried or concerned that in the next 2 months you may not have stable housing that you own, rent, or stay in as part of a household?" (2) "How likely do you think it would be that you would have to use a homeless shelter in the next 6 months?" | Predictive | 2 | Byrne et al.[148] |
| Self-reported drinking[a] | Substanc e use | Alcohol | Self-reported drinking within 6 hours prior to the event was obtained from the following dichotomous question "In the 6 hours before your injury, did you have any alcoholic beverages to drink - even one drink? This includes wine, beer, hard liquor, and any other | Discriminative | - | Cherpitel et al. [149] |

| | | | | | | |
|---|---|---|---|---|---|---|
| | | | beverages containing alcohol?" Additionally, data were obtained regarding the number of drinks consumed by beverage type and the time elapsed between the last drinks and the arrival in the ED<br><br>Usual quantity and frequency were obtained from questions having to do with the frequency of any drinking in the last year and the number of standard drinks usually consumed on an occasion. | | | |
| Self-reported Emergency Department Assessment of Chest Pain Score (S-EDACS) | Pain | Chest pain | S-EDACS relies solely on the responses to yes/no questions assessing patient-reported chest pain characteristics and cardiovascular risk factors. Given that no further interpretation of clinical symptoms, physical examination findings or troponins are required, EDACS can in fact be self-reported by untrained laypersons like patients. | Discriminative | 10 | Ng et al. [150] |
| Self Reporting Questionnaire (SRQ) | Mental health | General | SRQ has been developed by WHO as an instrument designed to screen for psychiatric disturbance, especially in developing countries. | Discriminative | 20 | van der Westhuizen et al. [151] |
| Short-Form Health Survey (SF-8) | Health and symptoms | General health | SF-8 instrument yields a composite measure of both physical and mental health domains. Higher scores on the SF-8 indicate more favorable self-reported health status. | Discriminative | 8 | Trautman et al.[57] |
| Short-Form 36 health survey (SF-36) | Health and symptoms | General health | SF-36 assesses 8 health domains. | Discriminative and predictive | 36 | McLean et al.[138]<br>Clemens et al.[63] |

| | | | | | | |
|---|---|---|---|---|---|---|
| Short two-question scale" or Brief two question depression screen[a] | Mental health | Depression | A short two-question scale screening for depressive symptoms. The questions were 1) ‘‘During the past month, have you been bothered by feeling down, depressed, or hopeless?’’ and 2) ‘‘During the past month, have you often been bothered by little interest or pleasure in doing things?’’ | Discriminative | 2 | Haughey et al.[152] Hustey et al.[153] |
| Short-Woman Abuse Screening Tool (short-WAST) | Partner violence | | short-WAST is a screening tool derived from the original WAST, designed to more efficiently identify women experiencing IPV. | Discriminative | 2 | Halpern et al.[116] Houry et al.[117] Mills et al.[76] Trautman et al.[57] |
| Sickness Impact Profile Alertness Behavior subscale (SIPab) | Health and symptoms | Symptoms | SIPab was used to evaluate postinjury cognitive symptoms, including confusion, disorientation, forgetfulness, slow reactivity, difficulty completing tasks, reason and problem-solving difficulties, and problems with concentration and thinking | Discriminative | - | McLean et al.[138] |
| Single Drug Screening Question (SDSQ) | Substance use | Drugs | SDSQ is a single-item screening tool for substance use | Discriminative | 1 item | Seale et al.[154] |
| Single-item literacy screener (SILS) | Health literacy | | SILS – a simple and brief instrument – asks: “How often do you need to have someone help when you read instructions, pamphlets, or other written material from your doctor or pharmacy? | Discriminative | 1 item | Bonaccorsi et al.[155] Kiechle et al.[103] |
| Single Item Screen for Mobility (SIS-M) | Older people | Fall | SIS-M was taken from the EQ-5D-5L. Patients were asked: “How would you describe your mobility TODAY”. | Predictive | 1 | Dasgupta et al.[156] |
| Single screening question for problem drinking[a] | Substance use | Alcohol | A single question as screening instrument for hazardous drinking and current alcohol use disorder. "When was the last time you had more than X drinks in one day?" | Discriminative | 1 | Williams et al.[157] |

| | | | | | | |
|---|---|---|---|---|---|---|
| Slapped, Things, Threaten (STaT) | Partner violence | | STaT is a screen for IPV.<br>The STaT questions are: “Have you ever been in a relationship where your partner has pushed or **S**lapped you?” “Have you ever been in a relationship where your partner **T**hreatened you with violence?” and “Have you ever been in a relationship where your partner has thrown, broken or punched **T**hings? | Discriminative | 3 | Paranjabe et al.[158] |
| Social risk and social need survey tool | Social risk | | A social risk and social need screening tool for ED patients | Discriminative | 10 | Ciccolo et al.[159] |
| Standardized Assessment of Personality – Abbreviated Scale (SAPAS) | Mental health | General | SAPAS measures aspects of emotion, behavior and relationships associated with a diagnosis of personality disorders. | Discriminative | 8 | Sen et al.[160] |
| STarT Back Screening Tool (SBST) | Pain | Back pain | SBST classifies patients with low back pain (LBP) into low, medium or high risk of developing chronic LBP. | Discriminative and predictive | 9 | Medeiros et al.[113]<br>Tan et al. [161] |
| Stay Independent Questionnaire (SIQ-12) | Older people | Fall | SIQ-12 was developed by the Center for Disease Control and Prevention as part of an initiative to promote and facilitate fall screening and management within primary care settings. | Predictive | 12 | Dasgupta et al.[156] |
| Suicide risk questionnaire | Mental health | Suicide | The questionnaire includes items on Pain, Coping, Upset, Wish to live, Wish to die. | Predictive | 11 | Desjardins et al.[162] |
| **T** | | | | | | |
| TRAUMA (Skinner's five-item trauma score) | Substance use | Alcohol | TRAUMA indicates the presence of excessive drinking or alcohol abuse. | Discriminative | 5 | Borges et al.[19] |

| | | | | | | |
|---|---|---|---|---|---|---|
| TWEAK (Tolerance, Worried, Eye, Amnesia, K/Cut down) | Substance use | Alcohol | TWEAK is used to screen for harmful drinking<br>T-Tolerance “How many drinks can you hold?”<br>W-Worried “Have close friends or relatives worried or complained about your drinking in the past year?”<br>E-Eye-opener “Do you sometimes take a drink in the morning when you first get up?”<br>A-Amnesia - stands for blackouts - “Has a friend or family member ever told you about things you said or did while you were drinking that you could not remember?”<br>K-K/Cut Down “Do you sometimes feel the need to cut down on your drinking?” | Discriminative | 5 | Cremonte et al.[23]<br>Borges et al.[19] |
| Two-Item Conjoint Screen for Alcohol and Other Drug Problems (TICS) | Substance use | General | TICS is designed for use in health care settings and assesses substance use problems over the past 12 months. It screens individuals who do not have problematic substance abuse or dependence. Both items inquire about alcohol and other drug use simultaneously. | Discriminative | 2 | McCabe et al.[15] |
| Two-item screening tool (Tiedemann's rule)[a] | Older people | Fall | Patients were given one point if they answered “yes” to “Are you taking six or more medications?” and two points for answering “yes” to “Have you had two or more falls in the past year?” to give a cumulative score from 0 to 3. | Predictive | 2 | Solie et al.[163]<br>Tiedemann et al.[164] |
| **U** | | | | | | |
| Universal Violence Prevention Screening Protocol (UVPSP) | Partner violence | | UVPSP assesses a patient for physical violence, threat of violence, sexual violence, and emotional violence. | Discriminative | 5 | Houry et al.[34] |
| **V** | | | | | | |

| | | | | | | |
|---|---|---|---|---|---|---|
| Validated Screening Questions[a] | Health literacy | | Questions that can identify patients with low health literacy:<br>the first question assesses confidence in filling out medical forms, the second question assesses need for help in reading hospital materials, and the final question assesses difficulty understanding written information in trying to learn more about a medical condition. | Discriminative | 3 | Kiechle et al.[103] |
| The Dutch Safety Management System [Veiligheidsmanage mentsysteem] (VMS) screener | Older people | General | The VMS screener consists of four domains: risk of functional decline, risk of falling, risk of delirium, and risk of malnourishment. | Predictive | 13 | Schuijt et al.[165]<br>van Dam et al.[99]<br>van Dam et al. [100] |
| The shortened [Veiligheidsmanage mentsysteem] VMS-based screening tool | Older people | General | The shortened VMS screening tool was derived from the Dutch national VMS-based screening instrument and address the same four geriatric domains.<br>The shortened tool consists of five questions that concerns: (1) memory problems, (2) history of acute confusional state or delirium, (3) unintentional weight loss, (4) help in activities of daily living (ADL), and (5) falls in the past 6 months. | Predictive | 5 | Snijders et al.[166] |
| Verbal dyspnea scores (VDS) | Dyspnea | | VDS is a verbal 0–10 rating scale in the assessment of dyspnea.<br>“On a scale from 0 to 10 how bad is your shortness of breath (SOB). | Discriminative and evaluative | 2 | Saracino et al.[167]<br>Saracino et al.[168] |
| Verbal numeric rating scale (VNRS) | Pain | General | VNRS is used to assess pain and the effect of pain management. The patient rates their pain on a verbal numeric scale. | Discriminative and evaluative | 1 | Holdgate et al.[169] |
| Verbal rating scale (VRS) | Pain | General | VRS measures the severity of pain of 0 to 10. | Discriminative and evaluative | 1 | Masoudi Alavi et al.[170] |

| | | | | | | |
|---|---|---|---|---|---|---|
| Visual Analogue Scale (VAS) - 10 cm | Dyspnea | | VAS rates dyspnea severity. | Discriminative and evaluative | 1 | Greene et al.[2] |
| Visual Analogue Scale (VAS) | Pain | General | VAS rates pain severity by placing a mark.<br>Ranging from 0-10 og 0-100 or facial expressions | Discriminative | 1 | Al-Kalaldeh et al.[109]<br>Bahreini et al.[46]<br>Holdgate et al.[169] |
| Visual Analogue Scale (VAS) | Nausea | | VAS is a 100-mm line labeled “no nausea” at the left and “unbearable nausea” at the right, with ratings recorded as millimeters from the left and change to the left or right being recorded as negative or positive, respectively | Discriminative and evaluative | 1 | Meek et al.[12] |
| Vulnerable Elder Survey (VES-13) | Older people | Fall | VES-13 is used to predict the functional impairment of older adults and identify individuals at higher risk of falls. | Discriminative and predictive | 13 | Greenberg et al.[66] |
| **W** | | | | | | |
| Women abuse screening tool (WAST) | Partner violence | | WAST is used to screen for abuse within current marital/partner relationship. The instrument consists of eight items, but the two first questions make up the short-WAST questionnaire and assess the degree of relationship tension and the amount of difficulty that the woman and her partner have in working out arguments on a scale of 1–3. | Discriminative | 8 | Svavarsdottir et al.[171] |

[a] PROMs with no identified name in the study.

[b] Computerized Adaptive Testing (CAT) PROMs

[c] Utilized as both a PROM and a CAT

[d] Based on the descriptions of PROM content provided in the studies

Abbreviations: Questions (Q), Intimate partner violence (IPV), Emergency Department (ED), not available (-), Post Traumatic Stress Disorder (PTSD).

## References

1. O'Connor L, Larkin C, Ibrahim AF, et al. Development and pilot study of simple suicide risk rulers for use in the emergency department. Gen Hosp Psychiatry 2020;63:97–102.
2. Greene SJ, Mentz RJ, Limkakeng AT, et al. Comparison of Dyspnea Measurement Instruments in Acute Heart Failure: The DYSPNEA-AHF Pilot Study. J Card Fail 2021;27(5):607–609.
3. Pacella-LaBarbara M, Larsen SE, Jaramillo S, et al. Event centrality following treatment for physical injury in the emergency department: Associations with posttraumatic outcomes. Gen Hosp Psychiatry 2020;67:77–82.
4. McCusker J, Bellavance F, Cardin S, et al. Prediction of hospital utilization among elderly patients during the 6 months after an emergency department visit. Ann Emerg Med 2000;36(5):438–45.
5. Doran KM, Johns E, Zuiderveen S, et al. Development of a homelessness risk screening tool for emergency department patients. Health Serv Res 2022;57(2):285–293.
6. Weiss SJ, Ernst AA, Cham E, et al. Development of a screen for ongoing intimate partner violence. Violence and victims 2003;18(2):131.
7. Juniper EF, Svensson K, Mörk AC, et al. Measuring health-related quality of life in adults during an acute asthma exacerbation. Chest 2004;125(1):93–97.
8. Devon HA, Rosenfeld A, Steffen AD, et al. Sensitivity, specificity, and sex differences in symptoms reported on the 13-item acute coronary syndrome checklist. J Am Heart Assoc 2014;3(2):e000586.
9. Cotte F, Mueller T, Gilbert S, et al. Safety of triage self-assessment using a symptom assessment app for walk-in patients in the emergency care setting: observational prospective cross-sectional study. JMIR mHealth and uHealth 2022;10(3):e32340.
10. Faqar-Uz-Zaman SF, Anantharajah L, Baumartz P, et al. The Diagnostic Efficacy of an App-based Diagnostic Health Care Application in the Emergency Room: eRadaR-Trial. A prospective, Double-blinded, Observational Study. Ann Surg 2022;276(5):935–942.
11. Fraser HSF, Cohan G, Koehler C, et al. Evaluation of Diagnostic and Triage Accuracy and Usability of a Symptom Checker in an Emergency Department: Observational Study. JMIR Mhealth Uhealth 2022;10(9):e38364.
12. Meek R, Egerton-Warburton D, Mee MJ, et al. Measurement and monitoring of nausea severity in emergency department patients: a comparison of scales and exploration of treatment efficacy outcome measures. Acad Emerg Med 2015;22(6):685–93.
13. Vaittinada Ayar P, Taille C, Vaittinada Ayar P, et al. Assessment of Predictor Factors Associated with Multiple Emergency Department Attendance with Asthma Attack: A Qualitative and Multicentric Prospective Observational Study. J Asthma Allergy 2022;15:303–313.
14. Singh SM, Bhalla A, Giri OP, et al. Development of Screening Questionnaire for Detection of Alcohol Dependence. J Clin Diagn Res 2015;9(9):VC07–VC10.
15. McCabe MP, Staiger PK, Thomas AC, et al. Screening for comorbid substance use disorders among people with a mental health diagnosis who present to emergency departments. Australasian Emergency Nursing Journal 2011;14(3):163–171.
16. Punches BE, Freiermuth CE, Sprague JE, et al. Screening for problematic opioid use in the emergency department: Comparison of two screening measures. J Am Coll Emerg Physicians Open 2024;5(1):e13106.
17. Tuli R, Romero SA, Figueroa C, et al. Investigating a Substance Abuse Screening in a Trauma Setting. Am Surg 2021;87(10):1606–1611.

18. Van Der Westhuizen C, Wyatt G, Williams JK, et al. Validation of the Alcohol, Smoking and Substance Involvement Screening Test in a low-and middle-income country cross-sectional emergency centre study. Drug and alcohol review 2016;35(6):702–709.
19. Borges G, Cherpitel CJ. Selection of screening items for alcohol abuse and alcohol dependence among Mexicans and Mexican Americans in the emergency department. J Stud Alcohol 2001;62(3):277–85.
20. Cherpitel CJ, Bazargan S. Screening for alcohol problems: comparison of the audit, RAPS4 and RAPS4-QF among African American and Hispanic patients in an inner city emergency department. Drug and Alcohol Dependence 2003;71(3):275–280.
21. Cherpitel CJ, Ye Y, Moskalewicz J, et al. Screening for alcohol problems in two emergency service samples in Poland: Comparison of the RAPS4, CAGE and AUDIT. Drug and Alcohol Dependence 2005;80(2):201–207.
22. Chung H, Lee JH. Clinical validation of screening test for at-risk drinking for young and middle-aged adults in an emergency department. Hong Kong Journal of Emergency Medicine 2020;30(4):203–209.
23. Cremonte M, Cherpitel CJ. Performance of screening instruments for alcohol use disorders in emergency department patients in Argentina. Subst Use Misuse 2008;43(1):125–38.
24. Fulbrook P, Lawrence P, Watt K. Validity of the Paddington Alcohol Test in an Australian Emergency Departmentdagger. Alcohol Alcohol 2015;50(4):407–12.
25. Lee KW, Choi YH, Lee JH. Cut-off points for screening at-risk drinking by AUDIT-C Korean version at emergency department. Turk J Emerg Med 2018;18(2):57–61.
26. Meneses-Gaya C, Crippa JA, Zuardi AW, et al. The fast alcohol screening test (FAST) is as good as the AUDIT to screen alcohol use disorders. Subst Use Misuse 2010;45(10):1542–57.
27. Neumann T, Neuner B, Gentilello LM, et al. Gender differences in the performance of a computerized version of the alcohol use disorders identification test in subcritically injured patients who are admitted to the emergency department. Alcohol Clin Exp Res 2004;28(11):1693–701.
28. Shenvi CL, Weaver MA, Biese KJ, et al. Identification and characterization of older emergency department patients with high-risk alcohol use. J Am Coll Emerg Physicians Open 2020;1(5):804–811.
29. Brousse G, Arnaud B, Geneste J, et al. How CAGE, RAPS4-QF, and AUDIT Can Help Practitioners for Patients Admitted with Acute Alcohol Intoxication in Emergency Departments? Front Psychiatry 2014;5:72.
30. Meneses-Gaya C, Zuardi AW, Loureiro SR, et al. Is the full version of the AUDIT really necessary? Study of the validity and internal construct of its abbreviated versions. Alcohol Clin Exp Res 2010;34(8):1417–24.
31. Lee J, Min S, Ahn JS, et al. Identifying alcohol problems among suicide attempters visiting the emergency department. BMC Psychiatry 2019;19(1):350.
32. Rodriguez-Martos A, Santamarina E. Does the short form of the Alcohol Use Disorders Identification Test (AUDIT-C) work at a trauma emergency department? Subst Use Misuse 2007;42(6):923–32.
33. Randall JR, Rowe BH, Colman I. Emergency department assessment of self-harm risk using psychometric questionnaires. The Canadian Journal of Psychiatry 2012;57(1):21–28.

34. Houry D, Kemball RS, Click LA, et al. Development of a brief mental health screen for intimate partner violence victims in the emergency department. Academic Emergency Medicine 2007;14(3):202–209.
35. Boudreaux ED, De Beurs DP, Nguyen TH, et al. Applying Computer Adaptive Testing Methods to Suicide Risk Screening in the Emergency Department. Suicide Life Threat Behav 2019;49(4):917–927.
36. Chang BP, Tan TM. Suicide screening tools and their association with near-term adverse events in the ED. Am J Emerg Med 2015;33(11):1680–1683.
37. Jensen AN, Kallemose T, Frostholm L, et al. Correlation between illness perceptions and self-reported degree-of-worry in somatic ill patients in emergency care: a Danish cross-sectional study. BMJ open 2024;14(10):e089595.
38. Azizoddin DR, Beck M, Flowers KM, et al. Psychological Evaluation of Patients With Cancer Presenting to the Emergency Department With Pain: Independent Predictors of Worse Pain Severity, Interference, and Higher Hourly Opioid Administration. JCO Oncol Pract 2022;18(10).
39. Boyd M, Koziol-McLain J, Yates K, et al. Emergency department case-finding for high-risk older adults: the Brief Risk Identification for Geriatric Health Tool (BRIGHT). Acad Emerg Med 2008;15(7):598–606.
40. Sattler AF, Bentley JP, Young J. Validation of a brief screening instrument for adult psychopathology. Professional Psychology: Research and Practice 2019;50(5):315–322.
41. Broderick KB, Richmond MK, Fagan J, et al. Pilot Validation of a Brief Screen Tool for Substance Use Detection in Emergency Care. J Emerg Med 2015;49(3):369–74.
42. Curiati PK, Arruda MDS, Carpenter CR, et al. Predicting posthospitalization falls in Brazilian older adults: External validation of the Carpenter instrument. Acad Emerg Med 2024;31(7):688–695.
43. Dionne CE, Le Sage N, Franche RL, et al. Five questions predicted long-term, severe, back-related functional limitations: evidence from three large prospective studies. J Clin Epidemiol 2011;64(1):54–66.
44. Reyes-Gibby CC, Anderson KO, Todd KH. Risk for Opioid Misuse Among Emergency Department Cancer Patients. Acad Emerg Med 2016;23(2):151–8.
45. Dresden SM, Platts-Mills TF, Kandasamy D, et al. Patient Versus Physician Perceptions of Frailty: A Comparison of Clinical Frailty Scale Scores of Older Adults in the Emergency Department. Acad Emerg Med 2019;26(9):1089–1092.
46. Bahreini M, Jalili M, Moradi-Lakeh M. A comparison of three self-report pain scales in adults with acute pain. J Emerg Med 2015;48(1):10–8.
47. Simpson SA, Goans C, Loh R, et al. Suicidal ideation is insensitive to suicide risk after emergency department discharge: Performance characteristics of the Columbia-Suicide Severity Rating Scale Screener. Acad Emerg Med 2021;28(6):621–629.
48. Hudon C, Bisson M, Dubois MF, et al. CONECT-6: a case-finding tool to identify patients with complex health needs. BMC Health Serv Res 2021;21(1):157.
49. Beiser DG, Ward CE, Vu M, et al. Depression in Emergency Department Patients and Association With Health Care Utilization. Acad Emerg Med 2019;26(8):878–888.

50. Beiser D, Vu M, Gibbons R. Test-Retest Reliability of a Computerized Adaptive Depression Screener. Psychiatr Serv 2016;67(9):1039–41.
51. O'Reilly LM, Dalal AI, Maag S, et al. Computer adaptive testing to assess impairing behavioral health problems in emergency department patients with somatic complaints. J Am Coll Emerg Physicians Open 2022;3(5):e12804.
52. Gibbons RD, Alegria M, Markle S, et al. Development of a computerized adaptive substance use disorder scale for screening and measurement: the CAT-SUD. Addiction 2020;115(7):1382–1394.
53. Lotfipour S, Howard J, Roumani S, et al. Increased detection of alcohol consumption and at-risk drinking with computerized alcohol screening. J Emerg Med 2013;44(4):861–6.
54. Thompson RG, Jr., Mullinax S, De Monte R, et al. Effectiveness of a Self-Administered Computerized Mental Health Screening Tool in the Emergency Department. Psychiatr Serv 2023;74(11):1180–1184.
55. Brucker K, Duggan C, Niezer J, et al. Assessing Risk of Future Suicidality in Emergency Department Patients. Acad Emerg Med 2019;26(4):376–383.
56. Chalmers CE, Mullinax S, Brennan J, et al. Screening Tools Validated in the Outpatient Pain Management Setting Poorly Predict Opioid Misuse in the Emergency Department: A Pilot Study. J Emerg Med 2019;56(6):601–610.
57. Trautman DE, McCarthy ML, Miller N, et al. Intimate partner violence and emergency department screening: computerized screening versus usual care. Ann Emerg Med 2007;49(4):526–34.
58. Friedmann PD, Saitz R, Gogineni A, et al. Validation of the screening strategy in the NIAAA "Physicians' Guide to Helping Patients with Alcohol Problems". J Stud Alcohol 2001;62(2):234–8.
59. Gamst-Jensen H, Trondarson T, Kallemose T, et al. How well do nurses know their patients? Agreement between patients' degree-of-worry and nurses' estimation of patients' degree-of-worry-An observational study. Scand J Caring Sci 2023;37(3):654–661.
60. Canagasaby A, Vinson DC. Screening for hazardous or harmful drinking using one or two quantity-frequency questions. Alcohol Alcohol 2005;40(3):208–13.
61. Boudreaux ED, Bedek KL, Gilles D, et al. The Dynamic Assessment and Referral System for Substance Abuse (DARSSA): development, functionality, and end-user satisfaction. Drug Alcohol Depend 2009;99(1-3):37–46.
62. Fabacher DA, Raccio-Robak N, McErlean MA, et al. Validation of a brief screening tool to detect depression in elderly ED patients. Am J Emerg Med 2002;20(2):99–102.
63. Clemens V, Saller MM, Meller R, et al. Clinical Acuity in the Emergency Department and Injury Severity Determine Hospital Admission of Older Patients with Low Energy Falls: Outcomes from a Prospective Feasibility Study. J Clin Med 2023;12(9).
64. Chapman J, Wakely M, Leonard K, et al. Great expectations: A multisite, randomized controlled open-label trial of a novel patient expectations communication tool. J Am Coll Emerg Physicians Open 2024;5(4):e13229.
65. Chan C, Chan Y, Au A, et al. Reliability and validity of the “Extended-Hurt, Insult, Threaten, Scream”(E-Hits) screening tool in detecting intimate partner violence in hospital emergency departments in Hong Kong. Hong Kong Journal of Emergency Medicine 2010;17(2):109–117.

66. Greenberg M, Jacoby J, Barraco RD, et al. Analysis of Falls Efficacy Scale and Vulnerable Elders Survey as Predictors of Falls. Cureus 2021;13(4):e14471.
67. Airagnes G, Le Faou AL, Duviviers E, et al. Does a standardized screening for alcohol use disorder at the Emergency Department inform the risk of repeated ED visit? Gen Hosp Psychiatry 2021;69:115–116.
68. Knightly R, Tadros G, Sharma J, et al. Alcohol screening for older adults in an acute general hospital: FAST v. MAST-G assessments. BJPsych Bull 2016;40(2):72–6.
69. Liu H, Shang N, Chhetri J, et al. A frailty screening questionnaire (FSQ) to rapidly predict negative health outcomes of older adults in emergency care settings. The journal of nutrition, health & aging 2020;24(6):627–633.
70. Shang N, Liu H, Wang N, et al. Comparison of three frailty screening instruments for prediction of adverse outcomes among older adults in the emergency department. Geriatr Gerontol Int 2022;22(10):851–856.
71. Kene M, Miller Rosales C, Wood S, et al. Feasibility of expanded emergency department screening for behavioral health problems. Am J Manag Care 2018;24(12):585–591.
72. Shah MN, Richardson TM, Jones CM, et al. Depression and cognitive impairment in older adult emergency department patients: changes over 2 weeks. J Am Geriatr Soc 2011;59(2):321–6.
73. Shrestha R, Shrestha AP, Shrestha A, et al. Unrecognized geriatric depression in the emergency Department of a Teaching Hospital in Nepal: prevalence, contributing factors, and metric properties of 5 item geriatric depression scale in this population. BMC Psychiatry 2020;20(1):533.
74. Ahmed A, Bushra A, Nisar N, et al. Adapting Health Literacy Tool for Use in Hospital: Experience of Holy Family Hospital Rawalpindi, Pakistan. Medical Forum Monthly 2021;32(10).
75. Aylward AF, Engelberg Anderson J, Morris A, et al. Using malnutrition and food insecurity screening to identify broader health-related social needs amongst older adults receiving emergency department care in the Southeastern United States: A cross-sectional study. Health Soc Care Community 2021;29(6):e420–e430.
76. Mills TJ, Avegno JL, Haydel MJ. Male victims of partner violence: prevalence and accuracy of screening tools. J Emerg Med 2006;31(4):447–52.
77. Perez Elias MJ, Gomez-Ayerbe C, Perez Elias P, et al. Development and Validation of an HIV Risk Exposure and Indicator Conditions Questionnaire to Support Targeted HIV Screening. Medicine (Baltimore) 2016;95(5):e2612.
78. Kaltiso SO, Greenbaum VJ, Moran TP, et al. Feasibility of a screening tool for sex trafficking in an adult emergency department. Acad Emerg Med 2021;28(12):1399–1408.
79. Gentile S, Lacroix O, Durand A, et al. Malnutrition: a highly predictive risk factor of short-term mortality in elderly presenting to the emergency department. The Journal of nutrition, health and aging 2013;17(4):290–294.
80. Graf CE, Giannelli SV, Chevalley T, et al. Identification of older patients at risk of unplanned readmission after discharge from the emergency department. Swiss medical weekly 2012;142(0102):w13327–w13327.

81. Bahadirli S, Kurt E, Rohat A, et al. Evaluation and comparison of screening tools used to predict the adverse outcomes of elderly patients in the emergency department. Acta Med Mediter 2021;37:1133–39.
82. Braes T, Flamaing J, Sterckx W, et al. Predicting the risk of functional decline in older patients admitted to the hospital: a comparison of three screening instruments. Age and ageing 2009;38(5):600–603.
83. Braes T, Moons P, Lipkens P, et al. Screening for risk of unplanned readmission in older patients admitted to hospital: predictive accuracy of three instruments. Aging clinical and experimental research 2010;22(4):345–351.
84. de Almeida Tavares JP, Sa-Couto P, Boltz M, et al. Portuguese older adults presenting at the emergency department: predictive validity of the Identification of Seniors at Risk (ISAR) tool. Journal of gerontological nursing 2017;43(8):18–24.
85. Demir Akça AS, Kafadar D, Kahveci FO, et al. Performance of Identification of Seniors at Risk tool to predict unplanned revisits to the emergency department in 6 months was low in predictive accuracy: A single-center study. Hong Kong Journal of Emergency Medicine 2021;28(2):72–78.
86. Dendukuri N, McCusker J, Belzile E. The identification of seniors at risk screening tool: further evidence of concurrent and predictive validity. Journal of the American Geriatrics Society 2004;52(2):290–296.
87. Heeren P, Devriendt E, Wellens NIH, et al. Old and New Geriatric Screening Tools in a Belgian Emergency Department: A Diagnostic Accuracy Study. J Am Geriatr Soc 2020;68(7):1454–1461.
88. Loddo S, Costaggiu D, Palimodde A, et al. Emergency department: risk stratification in the elderly. Journal of Gerontology and Geriatrics 2021;69(3):164–170.
89. McCusker J, Cardin S, Bellavance Fo, et al. Return to the emergency department among elders: patterns and predictors. Academic Emergency Medicine 2000;7(3):249–259.
90. Moons P, De Ridder K, Geyskens K, et al. Screening for risk of readmission of patients aged 65 years and above after discharge from the emergency department: predictive value of four instruments. European Journal of Emergency Medicine 2007;14(6):315–323.
91. Rizka A, Harimurti K, Pitoyo CW, et al. Comparison between the Identification of Seniors at Risk and Triage Risk Screening Tool in predicting mortality of older adults visiting the emergency department: Results from Indonesia. Geriatr Gerontol Int 2020;20(1):47–51.
92. Rizka A, Ambarwati RA, Mansjoer A, et al. Performance of Identification of the Seniors At Risk (ISAR) tool and Triage Risk-Screening Tool (TRST) for frail elderly emergency room patients. Infection 2021;70:S42–S45.
93. Salvi F, Morichi V, Grilli A, et al. Screening for frailty in elderly emergency department patients by using the Identification of Seniors At Risk (ISAR). The journal of nutrition, health & aging 2012;16(4):313–318.
94. Salvi F, Morichi V, Grilli A, et al. Predictive validity of the Identification of Seniors At Risk (ISAR) screening tool in elderly patients presenting to two Italian Emergency Departments. Aging clinical and experimental research 2009;21(1):69–75.
95. Salvi F, Morichi V, Lorenzetti B, et al. Risk stratification of older patients in the emergency department: comparison between the Identification of Seniors at Risk and Triage Risk Screening Tool. Rejuvenation research 2012;15(3):288–294.
96. Singler K, Heppner HJ, Skutetzky A, et al. Predictive validity of the identification of seniors at risk screening tool in a German emergency department setting. Gerontology 2014;60(5):413–9.

97. Suffoletto B, Miller T, Shah R, et al. Predicting older adults who return to the hospital or die within 30 days of emergency department care using the ISAR tool: subjective versus objective risk factors. Emergency Medicine Journal 2016;33(1):4–9.
98. Tavares JPA, Sa-Couto P, Boltz M, et al. Identification of Seniors at Risk (ISAR) in the emergency room: A prospective study. Int Emerg Nurs 2017;35:19–24.
99. van Dam CS, Moss N, Schaper SA, et al. Screening instruments for identification of vulnerable older adults at the emergency department: A critical appraisal. Acute Med 2018;17(3):124–9.
100. van Dam CS, Trappenburg MC, Ter Wee MM, et al. The accuracy of four frequently used frailty instruments for the prediction of adverse health outcomes among older adults at two Dutch emergency departments: findings of the AmsterGEM study. Annals of Emergency Medicine 2021;78(4):538–548.
101. Ennis N, Anton M, Bravoco O, et al. Prediction of posttraumatic stress and depression one-month post-injury: A comparison of two screening instruments. Health psychology 2021;40(10):702.
102. Platts-Mills TF, Nebolisa BC, Flannigan SA, et al. Post-Traumatic Stress Disorder among Older Adults Experiencing Motor Vehicle Collision: A Multicenter Prospective Cohort Study. Am J Geriatr Psychiatry 2017;25(9):953–963.
103. Kiechle ES, Hnat AT, Norman KE, et al. Comparison of brief health literacy screens in the emergency department. J Health Commun 2015;20(5):539–45.
104. Kendrick KR, Baxi SC, Smith RM. Usefulness of the modified 0-10 Borg scale in assessing the degree of dyspnea in patients with COPD and asthma. Journal of Emergency Nursing 2000;26(3):0216–0222.
105. Cloutier RL, Fuller BE, Hendrickson RG, et al. Measuring nausea in emergency department patients via the use of exploratory factor analysis. Acad Emerg Med 2010;17(6):e33–9.
106. White A, Afsari M, Balakrishnan H, et al. Implementation of universal screening for substance use in pregnancy in a public healthcare system. AJOG Glob Rep 2024;4(3):100384.
107. Stewart SH, Doscher A, Miles S, et al. Identification and risk-stratification of problem alcohol drinkers with minor trauma in the emergency department. West J Emerg Med 2010;11(2):133–7.
108. Ungar OJ, Cavel O, Oron Y, et al. A subjective rating scale for initial assessment of sudden unilateral sensorineural hearing loss. Audiology and Neurotology 2017;22(3):154–159.
109. Al-Kalaldeh M, Al-Zaidaneen H. Congruency of nurse–patient pain scoring in the emergency department: a cross-sectional observational study. Pain Management 2022;12(5):635–643.
110. Bijur PE, Latimer CT, Gallagher EJ. Validation of a verbally administered numerical rating scale of acute pain for use in the emergency department. Academic emergency medicine 2003;10(4):390–392.
111. Hsu HP, Cheng MT, Lu TC, et al. Pain Assessment in the Emergency Department: A Prospective Videotaped Study. West J Emerg Med 2022;23(5):716–723.
112. Ku NW, Cheng MT, Liew CQ, et al. Prospective study of pain and patient outcomes in the emergency department: a tale of two pain assessment methods. Scand J Trauma Resusc Emerg Med 2023;31(1):56.

113. Medeiros FC, Costa LOP, Oliveira IS, et al. The use of STarT BACK Screening Tool in emergency departments for patients with acute low back pain: a prospective inception cohort study. Eur Spine J 2018;27(11):2823–2830.
114. Boucher V, Lamontagne ME, Lee J, et al. Self-assessment of functional status in older emergency department patients: a cross-over randomized pilot trial. CJEM 2021;23(3):337–341.
115. Ernst AA, Weiss SJ, Cham E, et al. Detecting ongoing intimate partner violence in the emergency department using a simple 4-question screen: the OVAT. Violence Vict 2004;19(3):375–84.
116. Halpern LR, Susarla SM, Dodson TB. Injury location and screening questionnaires as markers for intimate partner violence. J Oral Maxillofac Surg 2005;63(9):1255–61.
117. Houry D, Feldhaus K, Peery B, et al. A positive domestic violence screen predicts future domestic violence. Journal of Interpersonal Violence 2004;19(9):955–966.
118. Flynn SB, Gordee A, Kuchibhatla M, et al. Moving toward patient-centered care in the emergency department: Patient-reported expectations, definitions of success, and importance of improvement in pain-related outcomes. Acad Emerg Med 2021;28(11):1286–1298.
119. Hyphantis T, Kotsis K, Kroenke K, et al. Lower PHQ-9 cutpoint accurately diagnosed depression in people with long-term conditions attending the Accident and Emergency Department. J Affect Disord 2015;176:155–63.
120. Hyphantis T, Kroenke K, Papatheodorou E, et al. Validity of the Greek version of the PHQ 15-item Somatic Symptom Severity Scale in patients with chronic medical conditions and correlations with emergency department use and illness perceptions. Compr Psychiatry 2014;55(8):1950–9.
121. Sung SC, Ma J, Earnest A, et al. Screening for panic-related anxiety in emergency department patients with cardiopulmonary complaints: A comparison of two self-report instruments. Psychiatry Res 2018;263:7–14.
122. Fox GWC, Rodriguez S, Rivera-Reyes L, et al. PROMIS Physical Function 10-Item Short Form for Older Adults in an Emergency Setting. J Gerontol A Biol Sci Med Sci 2020;75(7):1418–1423.
123. Boudreaux ED, Jaques ML, Brady KM, et al. The patient safety screener: validation of a brief suicide risk screener for emergency department settings. Archives of suicide research 2015;19(2):151–160.
124. Nock MK, Millner AJ, Ross EL, et al. Prediction of Suicide Attempts Using Clinician Assessment, Patient Self-report, and Electronic Health Records. JAMA Netw Open 2022;5(1):e2144373.
125. Kumodzi T, Kassam-Adams N, Vargas L, et al. Comparison of two screeners predicting the future development of depression and posttraumatic stress disorder in Black men after serious injury. Injury 2022;53(5):1678–1683.
126. Birmes P, Carreras D, Charlet J-P, et al. Peritraumatic dissociation and posttraumatic stress disorder in victims of violent assault. The Journal of nervous and mental disease 2001;189(11):796–798.
127. Ernst AA, Weiss SJ, Morgan-Edwards S, et al. Derivation and validation of a short emergency department screening tool for perpetrators of intimate partner violence: the PErpetrator RaPid Scale (PERPS). The Journal of emergency medicine 2012;42(2):206–217.

128. Jaramillo S, Suffoletto B, Callaway C, et al. Early Screening for Posttraumatic Stress Disorder and Depression Among Injured Emergency Department Patients: A Feasibility Study. Acad Emerg Med 2019;26(11):1232–1244.
129. Boyer L, Baumstarck K, Iordanova T, et al. A poverty-related quality of life questionnaire can help to detect health inequalities in emergency departments. J Clin Epidemiol 2014;67(3):285–95.
130. Schriger DL, Gibbons PS, Langone CA, et al. Enabling the diagnosis of occult psychiatric illness in the emergency department: a randomized, controlled trial of the computerized, self-administered PRIME-MD diagnostic system. Annals of emergency medicine 2001;37(2):132–140.
131. Beauchet O, Galery K, Vilcocq C, et al. PRISMA-7 and risk for short-term adverse events in older patients visiting the emergency department: results of a large observational and prospective cohort study. The Journal of nutrition, health and aging 2021;25(1):94–99.
132. Jarman H, Crouch R, Baxter M, et al. Feasibility and accuracy of ED frailty identification in older trauma patients: a prospective multi-centre study. Scand J Trauma Resusc Emerg Med 2021;29(1):54.
133. Allen MH, Abar BW, McCormick M, et al. Screening for suicidal ideation and attempts among emergency department medical patients: instrument and results from the Psychiatric Emergency Research Collaboration. Suicide Life Threat Behav 2013;43(3):313–23.
134. Cherpitel CJ. A brief screening instrument for problem drinking in the emergency room: the RAPS4. Rapid Alcohol Problems Screen. Journal of Studies on Alcohol 2000;61(3):447–449.
135. Cherpitel CJ, Ye Y, Bond J, et al. Cross-national performance of the RAPS4/RAPS4-QF for tolerance and heavy drinking: data from 13 countries. J Stud Alcohol 2005;66(3):428–32.
136. Cherpitel CJ, Borges G. Screening for drug use disorders in the emergency department: performance of the rapid drug problems screen (RDPS). Drug Alcohol Depend 2004;74(2):171–5.
137. Richards DK, Morera OF, Cabriales JA, et al. Factor, Concurrent and Predictive Validity of the Readiness to Change Questionnaire [Treatment Version] Among Non-Treatment-Seeking Individuals. Alcohol Alcohol 2020;55(4):409–415.
138. McLean SA, Kirsch NL, Tan-Schriner CU, et al. Health status, not head injury, predicts concussion symptoms after minor injury. Am J Emerg Med 2009;27(2):182–90.
139. Friedman BW, Schechter CB, Mulvey L, et al. Derivation of an abbreviated instrument for use in emergency department low back pain research: the five-item Roland Morris Questionnaire. Acad Emerg Med 2013;20(10):1013–21.
140. Poudel M, Bhandari R, Malla G, et al. Rose Angina Questionnaire: Validation in emergency department to detect myocardial infarction in a tertiary hospital of eastern Nepal. Hong Kong Journal of Emergency Medicine 2019;27(5):257–261.
141. Fulfer JL, Tyler JJ, Choi NJ, et al. Using indirect questions to detect intimate partner violence: the SAFE-T questionnaire. Journal of interpersonal violence 2007;22(2):238–249.
142. Chong E, Bao M, Goh EF, et al. SARC-F at the Emergency Department: Diagnostic Performance for Frailty and Predictive Performance for Reattendances and Acute Hospitalizations. J Nutr Health Aging 2021;25(9):1084–1089.
143. Weiner SG, Horton LC, Green TC, et al. A comparison of an opioid abuse screening tool and prescription drug monitoring data in the emergency department. Drug Alcohol Depend 2016;159:152–7.

144. Guo JW, Wallace AS, Luther BL, et al. Psychometric Evaluation of the Screener for Intensifying Community Referrals for Health. Eval Health Prof 2022;45(3):270–276.
145. Richards CT, Gisondi MA, Chang C-H, et al. Palliative care symptom assessment for patients with cancer in the emergency department: validation of the screen for palliative and end-of-life care needs in the emergency department instrument. Journal of palliative medicine 2011;14(6):757–764.
146. Mumma BE, Scofield ME, Mendoza LP, et al. Screening for Victims of Sex Trafficking in the Emergency Department: A Pilot Program. West J Emerg Med 2017;18(4):616–620.
147. Bae SJ, Kim E, Lee JH. Validation of the screening test for at-risk drinking in an emergency department using a tablet computer. Drug Alcohol Depend 2022;230:109181.
148. Byrne T, Hoang M, Montgomery AE, et al. Performance of 2 Single-Item Screening Questions to Identify Future Homelessness Among Emergency Department Patients. JAMA Netw Open 2022;5(8):e2226691.
149. Cherpitel CJ, Ye Y, Bond J, et al. Validity of self-reported drinking before injury compared with a physiological measure: cross-national analysis of emergency-department data from 16 countries. J Stud Alcohol Drugs 2007;68(2):296–302.
150. Ng M, Liu Z, Tan JSL, et al. Comparison of self-reported EDACS versus physician-reported EDACS for the triage of chest pain patients in the emergency department. J Am Coll Emerg Physicians Open 2021;2(2):e12393.
151. van der Westhuizen C, Wyatt G, Williams JK, et al. Validation of the Self Reporting Questionnaire 20-Item (SRQ-20) for Use in a Low- and Middle-Income Country Emergency Centre Setting. Int J Ment Health Addict 2016;14(1):37–48.
152. Haughey MT, Calderon Y, Torres S, et al. Identification of depression in an inner-city population using a simple screen. Acad Emerg Med 2005;12(12):1221–6.
153. Hustey FM. The use of a brief depression screen in older emergency department patients. Acad Emerg Med 2005;12(9):905–8.
154. Seale JP, Johnson JA, Cline N, et al. Drug screening and changing marijuana policy: Validation of new single question drug screening tools. Drug Alcohol Depend 2018;193:104–109.
155. Bonaccorsi G, Grazzini M, Pieri L, et al. Assessment of Health Literacy and validation of single-item literacy screener (SILS) in a sample of Italian people. Ann Ist Super Sanita 2017;53(3):205–212.
156. Dasgupta P, Frisch A, Huber J, et al. Predicting falls within 3 months of emergency department discharge among community-dwelling older adults using self-report tools versus a brief functional assessment. Am J Emerg Med 2022;53:245–249.
157. Williams R, Vinson DC. Validation of a single screening question for problem drinking. J Fam Pract 2001;50(4):307–12.
158. Paranjape A, Liebschutz J. STaT: a three-question screen for intimate partner violence. Journal of Women's Health 2003;12(3):233–239.
159. Ciccolo G, Curt A, Camargo CA, Jr., et al. Improving Understanding of Screening Questions for Social Risk and Social Need Among Emergency Department Patients. West J Emerg Med 2020;21(5):1170–1174.
160. Sen P, Barnicot K, Podder P, et al. Exploring the prevalence of personality disorder and the feasibility of using the SAPAS as a screening tool for personality disorder in an emergency department in India. Medicine, Science and the Law 2022;62(1):8–16.

161. Tan CIC, Liaw JSC, Jiang B, et al. Predicting outcomes of acute low back pain patients in emergency department: A prospective observational cohort study. Medicine (Baltimore) 2018;97(26):e11247.
162. Desjardins I, Cats-Baril W, Maruti S, et al. Suicide Risk Assessment in Hospitals: An Expert System-Based Triage Tool. J Clin Psychiatry 2016;77(7):e874–82.
163. Solie CJ, Swanson MB, Harland K, et al. Two-Item Fall Screening Tool Identifies Older Adults at Increased Risk of Falling after Emergency Department Visit. West J Emerg Med 2020;21(5):1275–1282.
164. Tiedemann A, Sherrington C, Orr T, et al. Identifying older people at high risk of future falls: development and validation of a screening tool for use in emergency departments. Emerg Med J 2013;30(11):918–22.
165. Schuijt H, Oud F, Bruns E, et al. Does the Dutch Safety Management Program predict adverse outcomes for older patients in the emergency department? Neth J Med 2020;78(5):244–50.
166. Snijders BMG, Emmelot-Vonk MH, Souwer ETD, et al. Prognostic value of screening instrument based on the Dutch national VMS guidelines for older patients in the emergency department. Eur Geriatr Med 2021;12(1):143–150.
167. Saracino A, Weiland T, Dent A, et al. Validation of a verbal dyspnoea rating scale in the emergency department. Emerg Med Australas 2008;20(6):475–81.
168. Saracino A, Weiland TJ, Jolly B, et al. Verbal dyspnoea score predicts emergency department departure status in patients with shortness of breath. Emerg Med Australas 2010;22(1):21–9.
169. Holdgate A, Asha S, Craig J, et al. Comparison of a verbal numeric rating scale with the visual analogue scale for the measurement of acute pain. Emerg Med (Fremantle) 2003;15(5-6):441–6.
170. Masoudi Alavi N, Aboutalebi MS, Sadat Z. Verbal and Non-verbal Rating Scales in the Determination of Pain Severity in Trauma Patients in the Emergency Department. Trauma Monthly 2017;22(1).
171. Svavarsdottir EK. Detecting intimate partner abuse within clinical settings: self-report or an interview. Scand J Caring Sci 2010;24(2):224–32.
