# Supplementary material for: Patient-Reported Outcome Measures in the Emergency Department: A Scoping Review
Source: J Am Coll Emerg Physicians Open. 2026 Aug 17;7(5):100486. doi: 10.1016/j.acepjo.2026.100486 (PMC13520099; doi:10.1016/j.acepjo.2026.100486)
Supplement: Supplementary file 6 [file mmc6.docx]

**Appendix index**

1. Table A1: Search strategy
   Detailed description of the search strategy in Embase (Ovid).
2. Table A2: Data extraction instrument
   Detailed description of the data extraction instrument used for all the included studies and included PROMs.
3. A3: all included studies
   A full list of all the studies included in the scoping review.
4. Table A4: Description of identified PROM by category, topic, subtopic, PROM name and patient group
5. Table A5: Characteristics of the included PROM
   A detailed description of all the included PROM by topic, subtopic, content, purpose and number of items (in alphabetical order).
